# Assessing collaborative efforts of making care fit for each patient: A systematic review

**DOI:** 10.1111/hex.13759

**Published:** 2023-04-16

**Authors:** Marleen Kunneman, Derek Gravholt, Sandra A. Hartasanchez, Michael R. Gionfriddo, Zoe Paskins, Larry J. Prokop, Anne M. Stiggelbout, Victor M. Montori

**Affiliations:** ^1^ Department of Biomedical Data Sciences Leiden University Medical Center Leiden The Netherlands; ^2^ Knowledge and Evaluation Research Unit Mayo Clinic Rochester Minnesota USA; ^3^ Division of Pharmaceutical, Social and Administrative Sciences, School of Pharmacy Duquesne University Pittsburgh Pennsylvania USA; ^4^ School of Medicine Keele University Keele UK; ^5^ Haywood Academic Rheumatology Centre Midlands Partnership NHS Foundation Trust Stoke‐on‐Trent UK; ^6^ Mayo Clinic Libraries Rochester Minnesota USA; ^7^ Erasmus School of Health Policy and Management Erasmus University Rotterdam Rotterdam The Netherlands

**Keywords:** making care fit, medical decision making, patient involvement, patient–clinician communication, shared decision making

## Abstract

**Introduction:**

For too many people, their care plans are designed without fully accounting for who they are, the lives they live, what matters to them or what they aspire to achieve. We aimed to summarize instruments capable of measuring dimensions of patient–clinician collaboration to make care fit.

**Methods:**

We systematically searched several databases (Medline, Embase, Cochrane, Scopus and Web of Science) from inception to September 2021 for studies using quantitative measures to assess, evaluate or rate the work of making care fit by any participant in real‐life clinical encounters. Eligibility was assessed in duplicate. After extracting all items from relevant instruments, we coded them deductively on dimensions relevant to making care fit (as presented in a recent Making Care Fit Manifesto), and inductively on the main action described.

**Results:**

We included 189 papers, mostly from North America (*N* = 83, 44%) and in the context of primary care (*N* = 54, 29%). Half of the papers (*N* = 88, 47%) were published in the last 5 years. We found 1243 relevant items to assess efforts of making care fit, included within 151 instruments. Most items related to the dimensions ‘Patient‐clinician collaboration: content’ (*N* = 396, 32%) and ‘Patient‐clinician collaboration: manner’ (*N* = 382, 31%) and the least related to ‘Ongoing and iterative process’ (*N* = 22, 2%) and in ‘Minimally disruptive of patient lives’ (*N* = 29, 2%). The items referred to 27 specific actions. Most items referred to ‘Informing’ (*N* = 308, 25%) and ‘Exploring’ (*N* = 93, 8%), the fewest items referred to ‘Following up’, ‘Comforting’ and ‘Praising’ (each *N* = 3, 0.2%).

**Discussion:**

Measures of the work that patients and clinicians do together to make care fit focus heavily on the content of their collaborations, particularly on exchanging information. Other dimensions and actions previously identified as crucial to making care fit are assessed infrequently or not at all. The breadth of extant measures of making care fit and the lack of appropriate measures of this key construct limit both the assessment and the successful implementation of efforts to improve patient care.

**Patient Contribution:**

Patients and caregivers from the ‘Making care fit Collaborative’ were involved in drafting the dimensions relevant to patient–clinician collaboration.

## 1 INTRODUCTION

The design of care plans often ignores who the patient is, the life they live, what matters to them or what they aspire to achieve. In other words, these care plans are generic, that is, for ‘patients like this’ rather than for ‘this patient’. When patients cannot accommodate the demands of living with illness and care, and of navigating the healthcare system, they may be unable to access and use healthcare services as configured and may not implement complicated treatments with sufficient fidelity.^1^ These effects will result in unfavourable biomedical and psychosocial outcomes, particularly amongst patients rendered vulnerable by unfair societal structures such as race or class discrimination and patients in challenging phases of life.^2^, ^3^, ^4^, ^5^ In the United States, for example, the high price of insulin to patients with diabetes forces one in four of them to ration this life‐saving medication.^6^ Similarly, a recent survey found that one‐third of adults report foregoing, and 4 in 10 delaying, recommended medical treatment due to cost.^7^ At the same time, patients with seemingly excellent ‘disease control’ and biomedical outcomes may reach these outcomes only at the expense of those aspects of life that make life worth living in the first place.^8^ An older man on a diuretic for cardiac insufficiency may stop taking his grandchild fishing because of urinary incontinence. A patient on a complex insulin regimen with frequent episodes of hypoglycemia may forego social outings to avoid ‘embarrassing disruptions’. These instances represent inadequate care as it fails to respond to the personal, medical and psychosocial needs of patients and to effectively weave care activities with daily life demands. Care is rendered inadequate when generic plans are offered, drawn from what is recommended for ‘patients like this’. Failure to carefully design care for ‘this patient’ is wasteful and harmful as care that does not fit is care patients do not need, want or cannot implement well.^9^, ^10^

Making care fit can take place at the ‘point of life’, a practice of self‐management mostly in the patient's personal environment which can be assisted, when pertinent, by family, friends and colleagues. In this personal setting, patients usually (re)consider the rational, emotional and practical sense of their care. The patient is usually the only person able to link up and coordinate these efforts with efforts to make care fit that take place at the ‘point of care’. To this end, patients and clinicians collaborate during clinical encounters to co‐create plans of care and treatment.^11^ Unless raised during these encounters, patients' trials and successes in making care fit at the point of life will remain largely invisible to clinicians at the point of care and, thus, will be left unconsidered when designing plans of care to address the patient's situation.^9^

Recently, an international and interdisciplinary group of patients, caregivers, clinicians, researchers, healthcare designers and policymakers published the ‘Making Care Fit Manifesto’.^12^ They describe that to make care fit, patients and clinicians work together in designing care, making sure their plans maximally respond to the patient's unique situation and priorities and minimally disrupt the patient's lives and social networks. This is an ongoing and iterative process, where patients and clinicians continuously re‐evaluate whether care still fits in people's lives and whether people's lives can still be lived alongside these plans of care.^8^, ^12^

To evaluate the extent to which making care fit takes place in clinical encounters and the efficacy of interventions to improve these efforts, we need reliable and valid instruments that measure all key dimensions of making care fit. While many measurement instruments may be available to assess specific components of patient–clinician collaboration,^13^, ^14^ it is unclear whether and how they measure the full breadth of efforts to make care fit.^12^, ^15^ We set out to inventory available items and instruments capable of measuring all key dimensions of making care fit.

## 2 METHODS

The conduct of this review followed a registered protocol (PROSPERO CRD42021236192) and the review report adheres to the Preferred Reporting Items for Systematic reviews and Meta‐Analyses statement.^16^

### 2.1 Eligibility criteria

We sought to include any study (or protocol of study) evaluating the occurrence, quality or satisfaction with behaviours potentially related to making care fit during a clinical encounter between patients and clinicians (any health professional in direct care interaction with patients) from any perspective (i.e., patient, caregiver, clinician, third‐party observer) using any measurement instrument. We did not institute language restrictions. We excluded encounters with simulated participants or participation. Table 1 summarizes our eligibility criteria (see also Supporting Information: Appendix A for a detailed description).

**Table 1:** Paper inclusion and exclusion criteria (in hierarchical order).

Criteria	Include	Exclude
A. (A protocol of) a research study	(Protocols of) original studies	Viewpoint papers and literature reviews
B. With real patients and clinicians	Any patient (in‐/out‐) and any clinician	Studies with simulated participants or decisions
C. Evaluating a specific encounter	Any (in‐person or virtual) meeting	Studies evaluating the quality of care (trajectories)
D. Evaluating behaviour	Occurrence of or satisfaction with communicative or collaborative behaviour	Objective/subjective medical outcomes, general satisfaction with hospital services, or preferences for behaviour
E. (At least) quantitative	Quantitative or mixed methods studies	Qualitative studies or case studies

### 2.2 Study identification

We used database subject headings supplemented with keywords to conduct a comprehensive search for eligible reports in the following databases from their inception until 21 September 2021: Ovid MEDLINE(R) and Epub Ahead of Print, In‐Process & Other Non‐Indexed Citations, and Daily, Ovid EMBASE, Ovid Database of Systematic Reviews, Scopus and Web of Science. An experienced librarian (L. J. P.) designed and conducted the search strategy with input from the study's principal investigator (M. K.). Supporting Information: Appendix B describes the search strategy.

### 2.3 Selection of studies

Pairs of reviewers assessed the eligibility of papers independently and in duplicate (M. K. or D. G. and S. A. H., M. R. G. or Z. P.). In case of disagreement, papers were screened by a third reviewer (M. K. or D. G.). We only excluded papers if two reviewers agreed to exclude them. Full texts of papers in a language other than English were reviewed by one reviewer only (from our team or our network, see Acknowledgements) unless that reviewer suggested a second opinion.

### 2.4 Data extraction

From each paper, one reviewer (M. K., D. G., S. A. H., M. R. G. or Z. P.) extracted descriptive data (location of the study, clinical context, year of publication) and data on the instruments used to evaluate behaviours during clinical care encounters (name of the instrument [or concept aiming to measure], reference to the publications describing the instrument's development [or ‘self‐developed’], if relevant any changes made to the instrument, items of the instrument, response options, target respondents), always erring on inclusion. If the reviewer found no instrument relevant to our objectives, or if the reviewer was unable to find all data of interest, a second reviewer reviewed the paper.

We extracted data on each relevant instrument only once. We extracted items from the first identified paper that used the instrument and recorded which other papers also described its use. Unless the authors stated they adapted items, we did not compare the wording of the items between the papers or with the original development or validation paper of that instrument.

In case of missing data, we checked whether the authors referred to another paper on the development, validation or use of the instrument of interest. If this approach did not lead to the data, we contacted the authors. If the corresponding author could not be reached, we contacted the first and/or last author. We sent a reminder after 2 weeks and reported data as missing if we received no response after 4 weeks. Papers and instruments were excluded only if the actual measurement items were missing.

### 2.5 Patient involvement

This systematic review builds on the ‘Making Care Fit Manifesto’.^12^ This Manifesto was written by an international and interdisciplinary Collaborative of patients, caregivers, clinicians, researchers, designers and policymakers. The Collaborative consisted of 25 people from seven countries, each with a unique life or work experience. Over the course of 2 days, they used several co‐creation methods to formulate dimensions relevant to patient–clinician collaboration to make care fit well, and to prioritize future work in this area, including work on relevant outcomes and evaluation of effects, and supporting patient–clinician collaboration.^12^ Details on the characteristics of the Collaborative and the methods used are reported in the Manifesto and its online supplement.^12^ The Collaborative's dimensions and priorities are at the core of this review's focus.

### 2.6 Analysis

We used a deductive and inductive framework method to analyze our data,^17^ the unit of our analyses being individual items from all extracted instruments. This approach was chosen to inventory available items capable of measuring the key dimensions of making care fit as identified by the Collaborative.^12^ In addition, this method leaves space to discover other unexpected dimensions that add to the experts' opinions and experiences. The two reviewers leading the analysis (M. K. and D. G.) are part of the ‘Making care fit Collaborative’, as is a third author (V. M. M.).

#### 2.6.1 Eligible items

First, two reviewers (M. K. and D. G.) independently and in duplicate reviewed all items and excluded items not assessing (1) encounters between (real‐life) patients and clinicians (e.g., ‘Length of time spent waiting at the office’), (2) a specific clinical care encounter (e.g., ‘I intend to follow the doctor's instructions’) or (3) specific behaviours (e.g., ‘Your confidence in this care provider’). The exclusion process was hierarchical in that items were checked against criterion 1 first, then 2, then 3. We excluded items only if both reviewers agreed to exclude them.

#### 2.6.2 Dimensions of making care fit

Second, the two reviewers coded in consensus all items into dimensions previously identified as relevant to making care fit by the international and interdisciplinary making care fit working group (deductive coding, see Box 1).^12^ Since our search and selection focused on behaviours during specific care encounters, we expected to find no items in dimension 8 (consequences). In addition, we inductively coded where items did not fit one of the priori‐defined dimensions and created new dimensions if relevant.

BOX 1. **Dimensions relevant to making care fit, as presented in the ‘Making Care Fit Manifesto’^12^**

**Table:** 

For care to fit, care should be: *Maximally responsive to patients' unique situations* (*dimension 1*). It should reflect each patient's personal and medical backstory, and life circumstances. *Maximally supportive of patient priorities* (*dimension 2*). It places patients’ needs and wishes in the foreground, accounting for and supporting their capacity to cope, adapt and thrive. It is congruent with each patient's values and their goals for life, well‐being and healthcare. It does not do harm. It draws from research evidence and guidelines for ‘patients like this’ to flexibly form care for ‘this patient’. It knows that people vary in their valuation of life and care. *Minimally disruptive patient lives* (*dimension 3*). Through conversations, it understands that care contributes to how life is lived or aimed to be lived. It understands that patients have a finite and varying capacity to prevent disruption, cope and adapt. *Minimally disruptive of patients' loved ones and social networks* (*dimension 4*). It is inclusive of and flexibly supports each patient's community of care, including their loved ones. It is not bound by the healthcare setting, but instead respectfully enters the patient's life space to support the work that patients do both in and with their community to make care fit. Making care fit: *Requires patients (and their loved ones) and clinicians to collaborate (for the purpose of this review, split in ‘content’* (*dimension 5*) *and ‘manner’* (*dimension 6*)). They use person‐sensitive communication, tailoring both the content and the manner of their conversation to their needs, abilities and situation. This conversation is potentially supported by tools. Care is built through equal patient–clinician relationships, mutual respect, willingness to accept each other's contributions, empathy, humanity and dignity. *Is an ongoing and iterative process* (*dimension 7*). People's needs, desires, capacities, capabilities and personal or medical situations may change. Care plans should therefore be flexible and continuously modified. Although the object of making care fit is to advance the situation of patients, the *consequences* of caring impact positively on patients, their loved ones, clinicians, and healthcare systems (*dimension 8*).

#### 2.6.3 Actions relevant to making care fit

Third, the two reviewers coded in consensus the main action described in each item (e.g., informing, exploring). Action terms were created inductively, based on item wording. Whenever a new action term was discovered, all previously coded items were checked again.

#### 2.6.4 Final check

Finally, we sorted all items according to their coded combination of dimension and action term. The two reviewers double‐checked the codes and in consensus changed any inconsistencies. All codings were then discussed amongst the research team.

## 3 RESULTS

### 3.1 Identified papers

**Figure 1:**
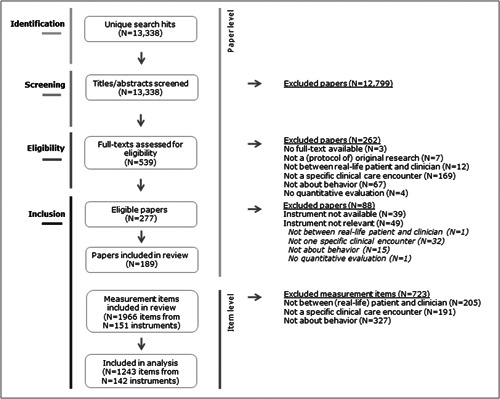
Study selection process.

Figure 1 depicts the study selection process. Our search yielded over 13,000 unique hits. Disagreements about inclusion occurred in 647 of 13,338 papers (5%) at the title and abstract screening phase, and in 156 of 539 papers (29%) at the full‐text screening phase. In the full‐text screening phase, we screened papers in 16 languages; 24 papers were in a language other than English.

We contacted the authors of 87 eligible papers for missing measurement items. The authors of 54 papers did not respond (62% of those contacted, 19% of eligible papers). Of these, 39 papers were excluded, and 15 were included as they described at least one other potentially relevant instrument in their paper. In total, 61 seemingly unique instruments remained unavailable for analyses (listed in Supporting Information: Appendix C).

We included 189 papers, mostly from North America (*N* = 83, 44%) and in the context of primary care (*N* = 54, 29%). Almost half of the papers (47%) were published in the last 5 years (see Table 2, detailed paper characteristics in Supporting Information: Appendix D).

**Table 2:** Study characteristics.

Study characteristics	*N* = 189
Location	
North America	83 (43.9%)
Europe	61 (32.3%)
Asia	30 (15.9%)
Africa	8 (4.2%)
Oceania	6 (3.1%)
Latin America	1 (0.5%)
Medical setting	
Primary care	52 (27.5%)
Multiple settings	22 (11.6%)
Oncology	16 (8.5%)
Musculoskeletal	11 (5.8%)
Endocrinology	10 (5.3%)
Psychiatry	8 (4.2%)
Gynecology and obstetrics	8 (4.2%)
Paediatrics	7 (3.7%)
Surgery	7 (3.7%)
Cardiovascular	7 (3.7%)
Other^a^	41 (21.6%)
Publication date	
1992–2010	56 (29.6%)
2011–2015	45 (23.8%)
2016≥	88 (46.5%)

^a^ All settings present in <3% of studies, including pulmonology, palliative care, dentistry, emergency, neurology, pharmacy, dermatology, immunization, nephrology, gastroenterology, geriatrics, haematology, intensive care, anaesthetics, ophthalmology, otorhinolaryngology, internal medicine, imaging, interventional radiology, urology.

### 3.2 Identified instruments and items

The included papers reported the use of 151 unique instruments of possible interest (median = 1, range 1–5 measures per paper). Of these, nine instruments were excluded at the analysis phase as none of their items met our eligibility criteria. We most often identified the SDMQ9 (*N* = 9 papers, nine‐item Shared Decision‐Making Questionnaire), COMRADE (*N* = 8, Combined Outcome Measure for Risk Communication and Treatment Decision making Effectiveness), CAHPS (*N* = 7, Consumer Assessment of Healthcare Providers and Systems), CARE (*N* = 7, Consultation and Relational Empathy) and DISQ (*N* = 7, Doctors' Interpersonal Skills Questionnaire) (see Supporting Information: Appendix E, for all identified instruments).

The 142 included instruments contained a total of 1243 items that could be used to evaluate efforts to make care fit (median = 7, range 1–56 relevant items per measure). The target respondents to these items were patients (*N* = 1001, 81%), proxies/caregivers (*N* = 74, 6%), clinicians (*N* = 74, 6%) and third‐party observers (*N* = 175, 14%).

### 3.3 Items to evaluate dimensions of making care fit

**Figure 2:**
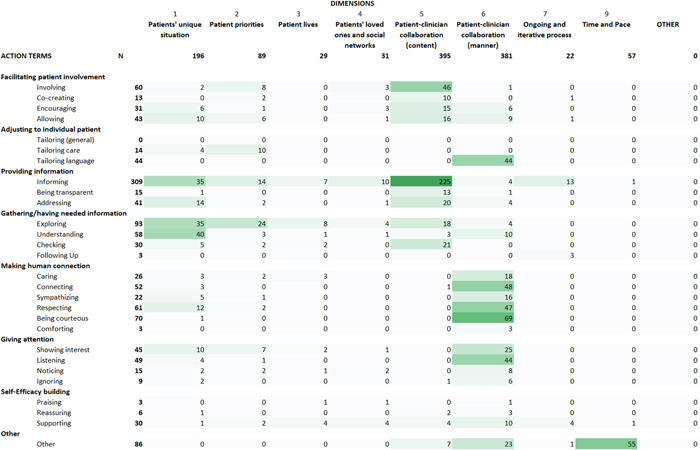
Heat map distribution of included measurement items across themes and action terms relevant to making care fit.

Figure 2 shows a heat map of the distribution of all included items across the dimensions and action terms. We identified the most items in dimension 5: patient–clinician collaboration ‘content’ (*N* = 396, 32%) and in dimension 6: patient–clinician collaboration ‘manner’ (*N* = 382, 31%). We identified the least items in dimension 7: ongoing and iterative process (*N* = 22, 2%) and in dimension 3: minimally disruptive of patient lives (*N* = 29, 2%).

In addition to our predefined dimensions from the Manifesto, we inductively created a dimension on ‘Time and Pace’ (*N* = 55, 4%), including for example ‘My doctor seemed to be in a hurry’ and ‘Did you have trouble understanding your doctors because they spoke too fast?’. Additionally, there were nine items (0.7%) that we categorized into the ‘Other’ dimension, for example, ‘The doctor gave me a chance to say what was really on my mind’ and ‘Helping you understand the importance of following his or her advice?’.

### 3.4 Items to evaluate actions of making care fit

Inductively, we identified 27 action terms used in the included items, which we grouped into seven overarching actions (see Figure 2). The action terms could relate to any party involved. For example, the term ‘Informing’ in dimension 5: patient–clinician collaboration ‘content’ included patient‐reported items like ‘I gave my opinion about the types of treatment or procedures the doctor was recommending’ (from measure #11, see Supporting Information: Appendix E and F), and ‘The doctor gave me enough information about the treatment choices available’ (from measure #2).

The most often identified action terms used in the items were ‘Informing’ (*N* = 309, 25%) and ‘Exploring’ (*N* = 93, 8%). The least often identified action terms were ‘Following up’, ‘Comforting’, and ‘Praising’ (each *N* = 3, 0.2%). There were 86 items that we were unable to categorize into action, for example, ‘She/he was available for me’, ‘Doctor frustrated with patient communication’ and ‘My doctor seemed to be in a hurry’.

As an illustration, Table 3 displays examples of items per unique dimension and action term combination. All included items are listed according to their coded dimension and action term in Supporting Information: Appendix F.

**Table 3:** Examples of items relevant to evaluating efforts of making care fit during clinical encounters are sorted by themes and action terms.

Action terms	Dimensions						
	Patients' unique situation	Patient priorities	Patient lives	Patients' loved ones and social networks	Patient–clinician collaboration (content)	Patient–clinician collaboration (manner)	Ongoing and iterative process
*Facilitating patient involvement*							
Involving	Discussed and agreed together what the problem was (19)	Did you and your doctor decide together which of your concerns were most important to you? (52)		Including your loved ones in decisions about your illness and treatment (15)	My doctor and I thoroughly weighed the different treatment options (1)	Patient communication categories—Active involvement: 1a Asking questions; 1b Concern; 1c Assertive responses; 1d Positive affect (22)	
Co‐creating		I set clear goals for my care together with the staff (44)			Discussed and reached an agreement with me on the plan of treatment (19)		The programme staff and I discussed my progress together and made changes as necessary (32)
Encouraging	My doctor encouraged me to talk about my concerns related to my condition (11)	My doctor discourages me from expressing my personal opinion about my medical condition (40)		Encouraged to go to a specific group or class to help me cope with my chronic illness (14)	My doctor encouraged me to give my opinion about treatment (11)	My doctor strongly encourages me to express all of my concerns about the prescribed treatment (40)	
Allowing	The doctor gave me enough chance to talk about all my problems (66)	The doctor gave me a chance to decide which treatment I thought was best for me (2)		Provide opportunities for the entire family to obtain information (25)	The doctor gave you the appropriate opportunity to ask questions about your treatment (89)	The doctor sometimes interrupted me (113)	Willing to let me ask questions via email/phone (107)
*Adjusting to the individual patient*							
Tailoring (general)	Tailoring information to the patient's situation (16)			Adapting to the needs and wishes of significant others (16)	The staff relied on my own assessment of how I felt (44)	The practitioner demonstrates sensitivity to talking about other issues (31)	
Tailoring care	Taking all your medical history into account when considering your current problem or treatment (96)	The care professional considered my preferences (20)					
Tailoring language						Did the doctors use medical words you did not understand? (7)	
*Providing information*							
Informing	Did you discuss any personal problems that may be related to your illness? (70)	Gave me as much information as I wanted (6)	Discussing how your problem or treatment impacts your daily life (96)	Give you information about the types of services offered at the organization or in your community (25)	The clinician explains the pros and cons of options to the patient (taking ‘no action’ is an option) (8)	During this visit, how often did the physician explain things in a way that could be easily understood? (120)	Discussed next steps including any follow‐up plans (47)
Being transparent	Your physician always told you everything about your illness, even if it is unpleasant (118)				The doctor spoke honestly about my illness and its treatment (113)	My doctor tells me everything; is truthful, up‐front and frank and does not keep things from me (54)	
Addressing	The doctor has relieved my worries about my illness (10)	My needs were addressed (59)		Answered family's questions about illness/treatment (67)	My doctor answered all my questions (92)	The doctor was willing to discuss my worries and fears (113)	
*Gathering/having needed information*							
Exploring	The doctor asked me for my ideas about my health problem (57)	Asked to talk about my goals in caring for my illness (14)	The doctor asked about how my illness affects my everyday life (66)	Exploring support needs of significant others (16)	The healthcare provider has asked me if I have questions and concerns about the procedure (76)	Exploring the patients’ feelings about treatment (16)	
Understanding	The care professional understood my problems and complaints (20)	This doctor clearly understands my health needs (21)	Did the doctors understand the kinds of problems you might have in doing the recommended treatment? (7)	This doctor knows a lot about the rest of my family (21)	Understood what I had to say (47)	The doctor understood what was on my mind (61)	
Checking	Did the doctors make sure you understand your health problems? (7)	Checking patients' preferences for treatment (16)	Checked to see if the treatment plan(s) was acceptable to me (47)		Checked to be sure I understood everything (6)		
Following up							Information exchange: The doctor told me to call back if I had any questions or problems (62)
*Making human connection*							
Caring	Concern the care provider showed for your questions or worries (17)	My doctor is usually considerate of my needs and puts them first (110)	The degree to which the medical staff cared about the medication's effects after I took the medication (87)			Healthcare provider really cares about me as a person (76)	
Connecting	I could tell this doctor about very personal problems (91)				I felt comfortable asking questions about my treatment and medications (45)	The doctor made me feel I could ask or say anything (113)	
Sympathizing	How often did your physician have empathy for your emotions and your current situation? (58)	When I receive prescriptions from my pharmacist, HCP shows concerns and attention to my medication needs (75)				Can view things from my perspective (see things as I see them) (117)	
Respecting	The doctor seemed to take my problems seriously (10)	Respecting the things in your life that are important to you (15)				The healthcare provider didn't show respect to what I have to say (76)	
Being courteous	My emotional needs (worries, fears, anxieties) were recognized and taken seriously by the programme staff (32)					The physician was polite (73)	
Comforting						The doctor put me at ease (91)	
*Giving attention*							
Showing interest	How often did your physician show an interest in your personal situation? (58)	He/she was interested in what I want from care (64)	Was interested in the effect of the problem on everyday activities (19)	Was interested in the effect of the problem on my family or personal life (19)		Showing interest in you as a person; not acting bored or ignoring what you have to say (18)	
Listening	Asking you questions about the reasons for your visit and listening carefully to your responses (53)	How much effort was made to listen to the things that matter most to you about your health issues? (33)				Listened carefully to what I had to say (47)	
Noticing	My pain was noticed and taken seriously (44)	She/he was attentive towards my needs (108)	Did staff pay attention to any possible emotional impact of fertility problems? (58)	My family was given enough attention (44)		I thought this doctor took notice of me as a person (35)	
Ignoring	The medical problems I had in the past were ignored during my visit (52)				My doctor ignores my opinion about treatment options (40)	Interpersonal skills: The doctor seemed to brush off my questions (62)	
*Self‐efficacy building*							
Praising			How much did your doctor let you know if he/she was pleased with your efforts to cope with your health problems? (52)	The doctor made me feel I have done a good job caring for my child		The nurse encourages the patient when s/he sees positive points (personal hygiene, taking medicine, compliance with the regulations of the ward) in the patient (85)	
Reassuring	She/he reassured me concerning my worries (108)				Made me feel my eye condition can be correctly treated (112)	The extent to which I felt reassured by this doctor was (24)	
Supporting	The AP helped me understand my condition (59)	I received help when I needed it (44)	Helping you to feel well so that you can perform your normal daily activities? (23)	Discussing how patient and significant others can cope with treatment together (16)	The physician helped me understand the results (13)	He/she gave me encouragement and transmitted optimism (64)	Helped to plan ahead so I could take care of my illness even in hard times (14)

*Note*: Numbers in brackets following the items refer to the instrument identification number as used in Supporting Information: Appendix D.

## 4 DISCUSSION

In this systematic review, we aimed to identify, characterize and summarize extant measures of patients and clinicians working together in designing care plans that fit. We found items that capture the content of patient–clinician collaboration (i.e., the characteristics of the care plans available) and the act of (multidirectional) informing. Only a handful of items assessed other dimensions relevant to making care fit, such as the extent to which patients and clinicians use an ongoing and iterative process and consider patients' lives, their loved ones and social networks in designing well‐fitted care plans. This paucity of adequate measures impairs medical research about the extent and quality of the work patients and clinicians do together to make care fit, as well as the effects of interventions to improve patient care. To our knowledge, this is the first comprehensive and systematic collection and characterization of measurement items likely to capture making care fit in clinical encounters.

The core of our review's focus was the ‘Making Care Fit Manifesto’, published by a diverse international Collaborative.^12^ In addition to the previously identified dimensions relevant to make care fit, our review showed the importance of appropriate time and pace in patient–clinician conversations, which we now included as the ninth dimension. Time has indeed often been identified as a main barrier to implementing patient–clinician collaboration, or shared decision‐making, in practice.^18^, ^19^, ^20^ Our review supports the notion that both the quantity (length) and quality (depth) of time matter in the care of patients and that making care fit for each patient requires unhurried conversations.^21^, ^22^

Our review additionally complements the *Manifesto* by identifying actions relevant to trying to make care fit during clinical encounters. One of every four included items assesses the act of informing. This is consistent with prior research and implementation efforts in patient involvement in decision‐making, where there also is a strong focus on exchanging information, discussing evidence, knowledge and rational decision‐making.^23^, ^24^, ^25^ Common identified actions such as informing, exploring, involving, and understanding are already included in some models of patient involvement in shared decision‐making.^26^, ^27^ However, our review brings attention to the breadth of actions that comprise patient–clinician collaboration and warrant support in daily practice such as noticing, supporting, tailoring, listening and respecting. We can only speculate as to why the latter actions, despite their rather obvious importance, are mostly absent from extant measures. Explanations may include but are not limited to the absence of a ‘making care fit’ domain in conceptual frameworks underpinning the instruments; instruments too narrow in scope that focus on clinical situations in need of acute care, that demand little self‐care, and which require minimal if any ongoing navigation through the healthcare system; lack of patient and caregiver involvement in the development of these measures and how, only recently, have academics become interested in the work of being a patient.^28^ Considering these additional actions advances the thinking and conceptualization of making care fit for each patient, contributing to progress from *whether* or *why* to *how*. These actions can now form a foundation for educators, clinicians and researchers to teach, develop instruments and assess the occurrence, extent and quality of the collaboration between patient and clinician to make care fit in daily practice.

Our seemingly comprehensive list of dimensions and actions may not fully represent the range and complexity of patient–clinician collaboration in designing care plans that fit. And yet, it represents, for now, a starting point for the development of an instrument to assess efforts to make care fit in practice. This instrument may need to include subscales to assess specific dimensions or actions, particularly to orient this assessment towards practice improvement. In doing so, we need to avoid improving processes without improving care.^29^ It is, therefore, crucial to focus also on the extent to which collaborative processes to make care fit contribute to care that actually fits.^25^, ^30^, ^31^ This can be achieved by assessing how responsive is the plan of care to the patient's unique situation and priorities (dimensions 1 and 2), and how disruptive it is to patients' lives, loves, and communities (dimensions 3 and 4) and vice versa.

Researchers interested in the field of patient–clinician collaboration can use this review and the supplemental repository to identify and select appropriate instruments or items for their studies. We designed our report to allow researchers to select items based on (i) a dimension relevant to making care fit and some or all its related actions, (ii) a specific action across all dimensions or (iii) a unique dimension‐action combination. Although it would be necessary to evaluate the measurement properties of instruments assembled de novo by combining items identified in this review, this indexed and referenced collection can contribute to improving efficiency and avoiding waste in research.

Our study has some limitations. Despite our comprehensive search and inclusive approach, we may have missed relevant instruments and items within instruments. We have identified at least 61 potentially relevant instruments which we could not access even after contacting the authors. We do not know if our pragmatic decision to focus on instruments that capture what happens during one specific encounter may have inadvertently excluded instruments designed to assess fit efforts that take place over time. We are keeping our inventory open and inviting the research community to submit other instruments or items for analysis and inclusion. Our analysis disconnected items from their parent measurement instruments. Therefore, we make no statements about the psychometric properties of the instruments in our review. Investigators would need to decide how important it is for the purposes of their studies to use either the pertinent items alone or as part of their parent instrument.

Strengths of our study include the comprehensive search, selection and data extraction without language limitations, and the duplicate, independent and reproducible judgements about the inclusion and classification of measures and items across themes and actions. Also, we transparently report all item classifications to improve their accuracy through peer revisions. Finally, our work benefited from the active involvement of patients, informal caregivers, and clinicians in composing the dimensions relevant to making care fit, ensuring the focus of our review is practice‐relevant and reflective of the real‐life variability of patient‐centred care.^12^

## 5 CONCLUSION

Research is not assessing the full breadth of patient and clinician efforts to make care fit and design care plans that reflect and respect who patients are, what lives they live, what matters to them or what they aspire to achieve. We found no instruments that could fully capture this work, only some items that appear to capture some aspects focused on the content of the consultation, particularly on providing information. This review not only documents this measurement gap but inaugurates an effort to close it to advance the science and practice of patient‐centred care.

## AUTHOR CONTRIBUTIONS

Marleen Kunneman secured funding, designed and conducted the study, analyzed the data and drafted the manuscript. Derek Gravholt designed and conducted the study, analyzed the data and provided input to the manuscript. Sandra A. Hartasanchez, Michael R. Gionfriddo and Zoe Paskins conducted the study and provided input to the manuscript. Larry J. Prokop designed the study (search strategy) and provided input to the manuscript. Anne M. Stiggelbout and Victor M. Montori provided input to the design of the study and to the manuscript. All authors approved the final manuscript. The corresponding author attests that all listed authors meet authorship criteria and that no others meeting the criteria have been omitted.

## CONFLICTS OF INTEREST STATEMENT

Marleen Kunneman had financial support from the Dutch Research Council (NWO) and The Netherlands Organisation for Health Research and Development (ZonMw) (#016.196.138) for the submitted work. Zoe Paskins is funded by the National Institute for Health and Care Research (NIHR) (Clinician Scientist Award (CS‐2018‐18‐ST2‐010)/NIHR Academy). All other authors received no financial support for the submitted work; all authors declare no financial relationships with any organizations that might have an interest in the submitted work in the previous 3 years; no other relationships or activities that could appear to have influenced the submitted work.

## Supporting information

Supporting information.

Supporting information.

## Data Availability

Datasets for this research are included in the Supporting Information: Appendix.
