# Supplementary material for: Assessing collaborative efforts of making care fit for each patient: A systematic review
Source: Health Expect. 2023 Apr 16;26(4):1391–403. doi: 10.1111/hex.13759 (PMC10349223; doi:10.1111/hex.13759)
Supplement: Supplementary file 2 — Supporting information. [file HEX-26--s001.docx]

**APPENDIX A.** Detailed paper inclusion – and exclusion criteria (in hierarchical order)

| **Criteria** | **Include** | **Exclude** |
| --- | --- | --- |
| 1. (a protocol of) a research study | - Original studies - Protocol papers of original studies | - Editorials - Viewpoints - (Systematic) reviews - Conference abstracts |
| 1. with real patients and clinicians | - Any patient (in-/out-) - Any clinician (including therapists) | - Vignette studies - Simulated patients/ clinicians - Student clinicians - Simulated decisions - Healthy people (lifestyle only) |
| 1. evaluating a specific encounter | - In-person or telemedicine meetings (irrespective of how long one visit lasted) | - Evaluating medical treatments - Evaluating care trajectories - What people think they generally do |
| 1. evaluating behaviour | (Occurrence of or satisfaction with: )   - Behaviours - Communication - Collaboration - PREMS | - Medical outcomes - PROMS - QoL - General satisfaction with hospital - Preferences for behaviour/ communication |
| 1. (at least) quantitative *note: even if scale/items are not explicitly mentioned*. | - Quantitative studies - Mixed methods studies | - Qualitative evaluation only - Case studies |

**APPENDIX B.** Search strategy

**Ovid**

Database(s): EBM Reviews - Cochrane Database of Systematic Reviews 2005 to September 15, 2021, Embase 1974 to 2021 September 20, Ovid MEDLINE(R) and Epub Ahead of Print, In-Process, In-Data-Review & Other Non-Indexed Citations and Daily 1946 to September 20, 2021
Search Strategy:

| **#** | **Searches** | **Results** |
| --- | --- | --- |
| 1 | exp patient satisfaction/ or exp patient preference/ or "treatment adherence and compliance"/ or treatment refusal/ or exp Patient Compliance/ | 533191 |
| 2 | (patient* adj3 (experience* or adherence or compliance or concordance or satisfaction or preference* or refusal or involvement or rating*)).ti,ab,kf. | 701082 |
| 3 | 1 or 2 | 1076359 |
| 4 | (((care or "shared decision*" or therap* or treatment* or communication) adj3 (appropriat* or effective or effectiveness or feasibility or feasible or fit or fitted or fitting or practical or reflect* or "high quality")) or "interpersonal skills" or "interpersonal communication").ti,ab,kf. | 1035253 |
| 5 | 3 and 4 | 63390 |
| 6 | exp "Appointments and Schedules"/ | 943829 |
| 7 | exp Physician-Patient Relations/ | 80279 |
| 8 | (appointment* or checkup* or "check-up*" or communication or consultation* or convers* or conversation* or deliberation* or dialog or dialogs or dialogue* or discourse* or discussion* or encounter or encounters or examination* or exchange or exchanges or interaction* or meeting* or rapport or relationship* or session* or "shared decision*" or visit or visits).ti,ab,kf. | 11659320 |
| 9 | 6 or 7 or 8 | 12431563 |
| 10 | 5 and 9 | 24959 |
| 11 | limit 10 to (conference abstract or editorial or erratum or note or addresses or autobiography or bibliography or biography or blogs or comment or dictionary or directory or interactive tutorial or interview or lectures or legal cases or legislation or news or newspaper article or overall or patient education handout or periodical index or portraits or published erratum or video-audio media or webcasts) [Limit not valid in CDSR,Embase,Ovid MEDLINE(R),Ovid MEDLINE(R) Daily Update,Ovid MEDLINE(R) PubMed not MEDLINE,Ovid MEDLINE(R) In-Process,Ovid MEDLINE(R) Publisher; records were retained] | 5460 |
| 12 | from 11 keep 1-17 | 17 |
| 13 | (10 not 11) or 12 | 19516 |
| 14 | limit 13 to yr="2018 -Current" | 5440 |
| 15 | remove duplicates from 14 | 3521 |
| 16 | limit 13 to yr="2013 -2017" | 5465 |
| 17 | remove duplicates from 16 | 3523 |
| 18 | limit 13 to yr="2008 -2012" | 3395 |
| 19 | remove duplicates from 18 | 2235 |
| 20 | 13 not (14 or 16 or 18) | 5216 |
| 21 | remove duplicates from 20 | 3527 |
| 22 | 15 or 17 or 19 or 21 | 12806 |

**Scopus**

1 TITLE-ABS-KEY(patient* W/3 (experience* or adherence or compliance or concordance or satisfaction or preference* or refusal or involvement or rating*))

2 TITLE-ABS-KEY(((care or "shared decision*" or therap* or treatment* or communication) W/3 (appropriat* or effective or effectiveness or feasibility or feasible or fit or fitted or fitting or practical or reflect* or "high quality")) or "interpersonal skills" or "interpersonal communication")

3 TITLE-ABS-KEY(appointment* or checkup* or "check-up*" or communication or consultation* or convers* or conversation* or deliberation* or dialog or dialogs or dialogue* or discourse* or discussion* or encounter or encounters or examination* or exchange or exchanges or interaction* or meeting* or rapport or relationship* or session* or "shared decision*" or visit or visits)

4 1 and 2 and 3

5 DOCTYPE(ab) OR DOCTYPE(ed) OR DOCTYPE(bk) OR DOCTYPE(er) OR DOCTYPE(no) OR DOCTYPE(sh)

6 4 and not 5

7 INDEX(embase) OR INDEX(medline) OR PMID(0* OR 1* OR 2* OR 3* OR 4* OR 5* OR 6* OR 7* OR 8* OR 9*)

8 6 and not 7

**Web of Science**

1. **TOPIC:** ((patient* NEAR/3 (experience* or adherence or compliance or concordance or satisfaction or preference* or refusal or involvement or rating*))) *AND* **TOPIC:** ((((care or "shared decision*" or therap* or treatment* or communication) NEAR/3 (appropriat* or effective or effectiveness or feasibility or feasible or fit or fitted or fitting or practical or reflect* or "high quality")) or "interpersonal skills" or "interpersonal communication")) *AND* **TOPIC:** ((appointment* or checkup* or "check-up*" or communication or consultation* or convers* or conversation* or deliberation* or dialog or dialogs or dialogue* or discourse* or discussion* or encounter or encounters or examination* or exchange or exchanges or interaction* or meeting* or rapport or relationship* or session* or "shared decision*" or visit or visits)) *AND* **DOCUMENT TYPES:** (Article  OR  Data Paper  OR  Proceedings Paper  OR  Review) Indexes=SCI-EXPANDED, ESCI Timespan=All years
2. PMID=(0* or 1* or 2* or 3* or 4* or 5* or 6* or 7* or 8* or 9*)
3. 1 not 2

**APPENDIX C.** Unavailable instruments and characteristics of excluded instruments (no response from the authors)

| **Instrument or Concept (if unnamed)** | **Reference ID** | **Full Reference** | **Location** | **Setting** |
| --- | --- | --- | --- | --- |
| - Patient satisfaction | 26 | Sultan AA, Acuna AJ, Samuel LT, Rabin JM, Grits D, Gurd DP, et al. (2020). "Utilization of Telemedicine Virtual Visits in Pediatric Spinal Deformity Patients: A Comparison of Feasibility and Patient Satisfaction at a Large Academic Center". J Pediatr Orthop.31:31. | United States | Paediatrics |
| - Health Literacy Assessment Questions (HLAQs) | 326 | Tavakoly Sany SB, Behzhad F, Ferns G, Peyman N. (2020). "Communication skills training for physicians improves health literacy and medical outcomes among patients with hypertension: a randomized controlled trial". BMC Health Serv Res.20(1):60. | Iran | Cardiovascular |
| - EORTC OUT-PATSAT-35 | 576 | Vardanega J, Henriques J, Pourcelot C, Dirand C, Nallet G, Bredart A, et al. (2019). "[Adjuvant hormonal therapy for early breast cancer: Assessment of patients' satisfaction]". Bull Cancer.106(12):1104-14. | France | Oncology |
| - Clinician’s communication skills | 629 | Hardee JT, Rehring TF, Cassara JE, Weiss K, Perrine N. (2019). "Effect and Durability of an In-depth Training Course on Physician Communication Skills". Perm.23. | United States | Multiple settings |
| - Patient’s satisfaction with consultation | 1906 | Mahmood LA, Casey D, Dolan JG, Dozier AM, Korones DN. (2016). "Feasibility of Early Palliative Care Consultation for Children With High-Risk Malignancies". Pediatr Blood Cancer.63(8):1419-22. | United States | Multiple settings |
| - Patient Satisfaction of Medical Interview Questionnaire | 2388 | Zamani AR, Motamedi N, Farajzadegan Z. (2015). "Routine programs of health care systems as an opportunity toward communication skills training for family physicians: A randomized field trial". J.4:71. | Iran | Primary care |
| - Patient - Doctor Communication Scale | 2445 | Martinez Y, Campbell SM, Hann M, Bower P. (2014). "The relationship between quality of care and self-management in patients with type 2 diabetes: a cross-sectional survey in primary care in Mexico". Qual Prim Care.22(6):262-9. | Mexico | Endocrinology |
| - Physician Communication Skill checklist - Patient Satisfaction | 2460 | Rezaei F, Askari HA. (2014). "Checking the relationship between physicians' communication skills and outpatients' satisfaction in the clinics of Isfahan Al-Zahra(S) Hospital in 2011". J.3:105. | Iran | Multiple settings |
| - Satisfaction with care | 2628 | Takacs L, Seidlerova JM, Smolik F, Hoskovcova S, Antonin P, Varakova J, et al. (2015). "[Satisfaction with perinatal care in Vysocina region in the period between October 2013 and September 2014]". Ceska Gynekol.80(6):426-35. | Czech Republic | Gynecology and obstetrics |
| - RIAS | 2927 | Grenness C, Hickson L, Laplante-Levesque A, Meyer C, Davidson B. (2015). "The nature of communication throughout diagnosis and management planning in initial audiologic rehabilitation consultations". J Am Acad Audiol.26(1):36-50. | Australia | Audiology |
| - Practitioner Satisfaction Survey - Patient Evaluation of the Quality of Diabetes Care Survey | 3437 | Leach MJ, Segal L, Esterman A, Armour C, McDermott R, Fountaine T. (2013). "The Diabetes Care Project: an Australian multicentre, cluster randomised controlled trial [study protocol]". BMC Public Health.13:1212. | Australia | Endocrinology |
| - Quality of Care | 3466 | Ejigu T, Woldie M, Kifle Y. (2013). "Quality of antenatal care services at public health facilities of Bahir-Dar special zone, Northwest Ethiopia". BMC Health Serv Res.13:443. | Ethiopia | Gynecology and obstetrics |
| - Patient Satisfaction with Care | 3469 | Szarka N, Nagykaldi Z, Vegh M, Oberling J. (2013). "[Patient satisfaction with care in gastrooesophageal reflux disease]". Orv Hetil.154(43):1713-8. | Hungary | Gastroenterology |
| - Social Competence Questionnaire (Polish) | 3882 | Bryl N, Horst-Sikorska W, Ignaszak-Szczepaniak M, Marcinkowska M, Michalak M, Sewerynek E. (2012). "Influence of social competence of physicians on patient compliance with osteoporosis medications--a study on Polish postmenopausal women". Ginekol Pol.83(7):511-6. | Poland | Gynecology and obstetrics |
| - Helping Alliance Questionnaire (HAQ) | 4112 | Weck F, Weigel M, Hautzinger M, Barocka A, Schlosser RGM, Stangier U. (2012). "Relapses in recurrent depression 1 year after psychoeducational treatment: the role of therapist adherence and competence, and the therapeutic alliance". Psychiatry Res.195(1-2):51-5. | Germany | Psychiatry |
| - PPAI Physician-Patient Alliance Inventory | 4406 | Bennett JK, Fuertes JN, Keitel M, Phillips R. (2011). "The role of patient attachment and working alliance on patient adherence, satisfaction, and health-related quality of life in lupus treatment". Patient Educ Couns.85(1):53-9. | United States | Rheumatology |
| - Measure of patient-centered communication (MPCC) | 5035 | Chapman BP, Duberstein PR, Epstein RM, Fiscella K, Kravitz RL. (2008). "Patient-centered communication during primary care visits for depressive symptoms: what is the role of physician personality?". Med Care.46(8):806-12. | United States | Primary care |
| - self developed based on SPIKES | 5876 | McCluskey L, Casarett D, Siderowf A. (2004). "Breaking the news: a survey of ALS patients and their caregivers". Amyotroph Lateral Scler Other Motor Neuron Disord.5(3):131-5. | United States | Neurology |
| - Patient satisfaction scale | 5889 | Garroutte EM, Kunovich RM, Jacobsen C, Goldberg J. (2004). "Patient satisfaction and ethnic identity among American Indian older adults". Soc Sci Med.59(11):2233-44. | United States | Multiple settings |
| - RIAS - Satisfaction | 6471 | Brown LD, de Negri B, Hernandez O, Dominguez L, Sanchack JH, Roter D. (2000). "An evaluation of the impact of training Honduran health care providers in interpersonal communication". Int J Qual Health Care.12(6):495-501. | Honduras | Multiple settings |
| - Doctor-Patient Communication | 6555 | Gopinath B, Radhakrishnan K, Sarma PS, Jayachandran D, Alexander A. (2000). "A questionnaire survey about doctor-patient communication, compliance and locus of control among south Indian people with epilepsy". Epilepsy Res.39(1):73-82. | India | Neurology |
| - RIAS   Patient Satisfaction | 6700 | Roter D, Rosenbaum J, de Negri B, Renaud D, DiPrete-Brown L, Hernandez O. (1998). "The effects of a continuing medical education programme in interpersonal communication skills on doctor practice and patient satisfaction in Trinidad and Tobago". Med Educ.32(2):181-9. | Trinidad and Tobago | Multiple settings |
| - Survey of Health and Medical Care for Veterans in Ambulatory Care - PACE Medical Visit Evaluation | 6855 | Rubenstein LV, Yano EM, Fink A, Lanto AB, Simon B, Graham M, et al. (1996). "Evaluation of the VA's Pilot Program in Institutional Reorganization toward Primary and Ambulatory Care: Part I, Changes in process and outcomes of care". Acad Med.71(7):772-83. | United States | Primary care |
| - Patient Satisfaction | 6887 | Buck D, Jacoby A, Baker GA, Graham-Jones S, Chadwick DW. (1996). "Patients' experiences of and satisfaction with care for their epilepsy". Epilepsia.37(9):841-9. | UK | Primary care |
| - Satisfaction | 7036 | Cockburn J, Hill D, De Luise T, Flint-Richter D. (1993). "Satisfaction of attenders during the establishment of an Australian mammography screening program". Aust J Public Health.17(2):103-8. | Australia | Oncology |
| - Child Satisfaction Questionnaire - Parent Medical Interview Satisfaction Scale - Physician Satisfaction with Medical Visit | 7105 | Lewis CC, Pantell RH, Sharp L. (1991). "Increasing patient knowledge, satisfaction, and involvement: randomized trial of a communication intervention". Pediatrics.88(2):351-8. | United States | Pediatrics |
| - Physician Dominance | 7119 | Oleinik A. (1990). "[Influence of changes in physicians' patterns of explanation on patients' compliance]". Harefuah.118(9):514-7. | Israel | Primary care |
| - Physician Interpersonal Skills | 7249 | Bartlett EE, Grayson M, Barker R, Levine DM, Golden A, Libber S. (1984). "The effects of physician communications skills on patient satisfaction; recall, and adherence". J Chronic Dis.37(9-10):755-64. | United States | Primary care |
| - Physician Behaviors | 7283 | DiMatteo MR, Hays R. (1980). "The significance of patients' perceptions of physician conduct: a study of patient satisfaction in a family practice center". J Community Health.6(1):18-34. | United States | Primary care |
| - Patient-Centeredness Questionnaire-Infertility | 7311 | Harzif AK, Shafira N, Mariana A, Lovita BT, Mutia HD, Maidarti M, et al. (2020). "Communication and respect for patient value as significant factors in patient-centered infertility care: A survey of patients' experiences in two infertility centers". Journal of Human Reproductive Sciences.13(1):22-5. | Indonesia | Gynecology and obstetrics |
| - Patient Satisfaction | 7515 | Pribadi P, Kristina SA, Syahlani SP, Satibi. (2019). "The empirical test of pharmacist-patient relationship model in hospital pharmacy practice: indonesia context". Research Journal of Pharmacy and Technology.12(10):4623-7. | Indonesia | Pharmacy |
| - Patient Satisfaction | 8701 | Roche TE, Gardner G, Lewis PA. (2015). "Effectiveness of an emergency nurse practitioner service for adults presenting to rural hospitals with chest pain: Protocol for a multicentre, longitudinal nested cohort study". BMJ Open.5 (2) (no pagination)(e006997). | Australia | Emergency |
| - Satisfaction and Experience with Communication with Medical Personnel | 8815 | Stilinovic M, Pipinic IS, Antabak A, Cavar S, Bogovic M, Medancic SS, et al. (2014). "Quality of communication at the Division of Pediatric Surgery, Zagreb University Hospital Center. [Croatian]". Paediatria Croatica.58(3):203-7. | Croatia | Pediatrics |
| - Cancer Diagnostic Interview Scale - Satisfaction with Diagnostic Consultation | 10622 | Mager WM, Andrykowski MA. (2002). "Communication in the cancer 'bad news' consultation: Patient perceptions and psychological adjustment". Psychooncology.11(1):35-46. | United States | Oncology |
| - Satisfaction with Medical Care | 11065 | Wasserman RC, Inui TS, Barriatua RD. (1984). "Pediatric clinicians' support for parents makes a difference: An outcome-based analysis of clinician-parent interaction". Pediatrics.74(6):1047-53. | United States | Multiple settings |
| - Patient-centeredness questionnaire - Client Satisfaction Questionnaire (CSQ-8) - Health Services Outpatient Experience Questionnaire | 11403 | John Thomas B, JayaKumar DS, Suganya G. (2017). "Effect of patient centredness on health service experience: Mediation of patient satisfaction". Br J Health Care Manage.23(12):591-600. | India | Ophthalmology |
| - OUT-PATSAT35 | 11763 | Thanh VFN, Bosset JF, Monnier A, Fournier J, Perrin V, Baumann C, et al. (2011). "Determinants of patient satisfaction in ambulatory oncology: a cross sectional study based on the OUT-PATSAT35 questionnaire". BMC Cancer.11. | France | Oncology |
| - CSS: Consultation Satisfaction Scale | 12309 | Juraskova I, Laidsaar-Powell R, Keast R, Schofield P, Costa DS, Kay J, et al. (2021). "eTRIO trial: study protocol of a randomised controlled trial of online education modules to facilitate effective family caregiver involvement in oncology". BMJ Open.11(5):e043224. | Australia | Oncology |
| - Patient Satisfaction (KPNC items, used the 6 questions related to patient-physician interactions) | 13219 | Glanternik JR, McDonald JC, Yee AH, Howell BA, Saba KN, Mellor RG, et al. (2020). "Evaluation of a Vaccine-Communication Tool for Physicians". J Pediatr.224:72-8.e1. | United States | Pediatrics |
| - Patient Satisfaction Questionnaire | 1820* | Plewnia A, Bengel J, Korner M. (2016). "Patient-centeredness and its impact on patient satisfaction and treatment outcomes in medical rehabilitation". Patient Educ Couns.99(12):2063-70. | Germany | Multiple settings |
| - Satisfaction with interpersonal care | 2023* | Ruberton PM, Huynh HP, Miller TA, Kruse E, Chancellor J, Lyubomirsky S. (2016). "The relationship between physician humility, physician-patient communication, and patient health". Patient Educ Couns.99(7):1138-45. | United States | Primary Care |
| - RIAS (Observer Checklist) | 2056* | Lussier M-T, Richard C, Glaser E, Roberge D. (2016). “The impact of a primary care e-communication intervention on the participation of chronic disease patients who had not reached guideline suggested treatment goals”. Patient Educ Couns.99(4):530-41. | Canada | Primary Care |
| - Satisfaction | 2386* | Warmington K, Kennedy CA, Lundon K, Soever LJ, Brooks SC, Passalent LA, et al. (2015). "The patient perspective: arthritis care provided by Advanced Clinician Practitioner in Arthritis Care program-trained clinicians". Open access rheumatol.7:45-53. | Canada | Rheumatology |
| - Client Satisfaction Questionnaire | 3484* | van der Krieke L, Emerencia AC, Boonstra N, Wunderink L, de Jonge P, Sytema S. (2013). "A web-based tool to support shared decision making for people with a psychotic disorder: randomized controlled trial and process evaluation". J Med Internet Res.15(10):e216. | Netherlands | Psychiatry |
| - Team scale - Questionnaire on Patient Satisfaction (ZUF-8) | 3711* | Quaschning K, Korner M, Wirtz M. (2013). "Analyzing the effects of shared decision-making, empathy and team interaction on patient satisfaction and treatment acceptance in medical rehabilitation using a structural equation modeling approach". Patient Educ Couns.91(2):167-75. | Germany | Musculoskeletal |
| - PEPPI Patient-Physician communication effectiveness in decision making - Index of the tangible informational support items | 3921* | Yanez B, Stanton AL, Maly RC. (2012). "Breast cancer treatment decision making among Latinas and non-Latina Whites: a communication model predicting decisional outcomes and quality of life". Health Psychol.31(5):552-61. | United States | Oncology |
| - PEECH instrument - UK NHS national patient survey | 3972* | Maben J, Adams M, Peccei R, Murrells T, Robert G. (2012). “’Poppets and parcels’: the links between staff experience of work and acutely ill older peoples’ experience of hospital care”. Int J Older People Nurs.7(2):83-94. | UK | Geriatrics |
| - DDPRQ-10 Difficult Doctor-Patient Relationship Questionnaire | 4626* | Bieber C, Muller KG, Nicolai J, Hartmann M, Eich W. (2010). "How does your doctor talk with you? Preliminary validation of a brief patient self-report questionnaire on the quality of physician-patient interaction". J Clin Psychol Med Settings.17(2):125-36. | Germany | Multiple settings |
| - RIAS | 5817* | Kindler CH, Szirt L, Sommer D, Hausler R, Langewitz W. (2005). "A quantitative analysis of anaesthetist-patient communication during the pre-operative visit". Anaesthesia.60(1):53-9. | Switzerland | Anesthetics |
| - RIAS | 6028* | Cooper LA, Roter DL, Johnson RL, Ford DE, Steinwachs DM, Powe NR. (2003). "Patient-centered communication, ratings of care, and concordance of patient and physician race". Ann Intern Med.139(11):907-15. | United States | Primary care |
| - RIAS | 6944* | Butow PN, Dunn SM, Tattersall MH, Jones QJ. (1995). "Computer-based interaction analysis of the cancer consultation". Br J Cancer.71(5):1115-21. | Australia | Oncology |
| - Client Satisfaction Questionnaire | 7368* | Shiozawa T, Yamaguchi S, Matsunaga A, Sawada U, Fujii C. (2020). "Development of the Interpersonal Processes of Care Survey-Japanese version". Neuropsychopharmacology Reports.40(1):107-12. | Japan | Psychiatry |
| - Parents’ Perceptions of Primary Care measure (P3C) | 10176* | Hart CN, Drotar D, Gori A, Lewin L. (2006). “Enhancing parent-provider communication in ambulatory pediatric practice”. Patient Education and Counseling.63(1-2):38-46. | United States | Pediatrics |
| - Diabetes-specific communication | 10470* | Piette JD, Schillinger D, Potter MB, Heisler M. (2003). "Dimensions of patient-provider communication and diabetes self-care in an ethnically diverse population". J Gen Intern Med.18(8):624-33. | United States | Endocrinology |

Note: * Papers included as they described at least one other potentially relevant instrument

**APPENDIX D.** Characteristics of included papers

| **Reference ID** | **Full Reference** | **Location** | **Setting** | **Instrument ID # (see appendix E)** |
| --- | --- | --- | --- | --- |
| 73 | An AW, Ladwig S, Epstein RM, Prigerson HG, Duberstein PR. (2020). "The impact of the caregiver-oncologist relationship on caregiver experiences of end-of-life care and bereavement outcomes". Support Care Cancer.03:03. | United States | Palliative care | 43 |
| 196 | Kunneman M, LaVecchia CM, Singh Ospina N, Abu Dabrh AM, Behnken EM, Wilson P, et al. (2019). "Reflecting on shared decision making: A reflection-quantification study". Health Expect.22(5):1165-72. | United States | Multiple settings | 33 |
| 311 | Vo MT, Uratsu CS, Estacio KR, Altschuler A, Kim E, Alexeeff SE, et al. (2019). “Prompting Patients with Poorly Controlled Diabetes to Identify Visit Priorities Before Primary Care Visits: a Pragmatic Cluster Randomized Trial”. J Gen Intern Med.34(6):831-8. | United States | Endocrinology | 70, 3, 14, 11, 68 |
| 559 | Eksteen LB, Mash RJ. (2019). "Evaluating the validity and reliability of the Medical Interview Satisfaction Scale in South African primary care consultations". Fam Pract.36(3):310-6. | South Africa | Primary Care | 10 |
| 649 | Maurici M, Arigliani M, Dugo V, Leo C, Pettinicchio V, Arigliani R, et al. (2019). "Empathy in vaccination counselling: a survey on the impact of a three-day residential course". Hum Vaccin Immunother.15(3):631-6. | Italy | Immunization | 4 |
| 679 | Burt J, Newbould J, Abel G, Elliott MN, Beckwith J, Llanwarne N, et al. (2017). "Investigating the meaning of 'good' or 'very good' patient evaluations of care in English general practice: a mixed methods study". BMJ Open.7(3):e014718. | UK | Primary Care | 50 |
| 710 | Community Pharmacy Medicines Management Project Evaluation T. (2007). "The MEDMAN study: a randomized controlled trial of community pharmacy-led medicines management for patients with coronary heart disease". Fam Pract.24(2):189-200. | UK | Cardiovascular | 109 |
| 767 | Denisov IN, Reze AG, Volnuhin AV, Azizova DI. (2019). "[The patients' evaluation of medical service at the out-patient level]". Probl Sotsialnoi Gig Istor Med.27(3):243-7. | Russia | Multiple settings | 5 |
| 847 | Aelbrecht K, Hanssens L, Detollenaere J, Willems S, Deveugele M, Pype P. (2019). "Determinants of physician-patient communication: The role of language, education and ethnicity". Patient Educ Couns.102(4):776-81. | Multiple Countries (Europe) | Primary Care | 73 |
| 875 | Casu G, Gremigni P, Sommaruga M. (2019). "The Patient-Professional Interaction Questionnaire (PPIQ) to assess patient centered care from the patient's perspective". Patient Educ Couns.102(1):126-33. | Italy | Multiple settings | 64 |
| 882 | Neri L, Peris K, Longo C, Calvieri S, Frascione P, Parodi A, et al. (2019). "Physician-patient communication and patient-reported outcomes in the actinic keratosis treatment adherence initiative (AK-TRAIN): a multicenter, prospective, real-life study of treatment satisfaction, quality of life and adherence to topical field-directed therapy for the treatment of actinic keratosis in Italy". J Eur Acad Dermatol Venereol.33(1):93-107. | Italy | Dermatology | 74 |
| 958 | Drewelow E, Santos S, Hornung A, Altiner A, Loffler C, Pentzek M, et al. (2018). "[Does place of residence have an influence on shared decision making for patients with type 2 diabetes in general practice?]". Z.137-138:36-41. | Germany | Endocrinology | 1 |
| 1081 | Leydon GM, Stuart B, Summers RH, Little P, Ekberg S, Stevenson F, et al. (2018). "Findings from a feasibility study to improve GP elicitation of patient concerns in UK general practice consultations". Patient Educ Couns.101(8):1394-402. | UK | Primary care | 10 |
| 1088 | Perez-Revuelta J, Villagran-Moreno JM, Moreno-Sanchez L, Pascual-Pano JM, Gonzalez-Saiz F. (2018). "Patient perceived participation in decision making on their antipsychotic treatment: Evidence of validity and reliability of the COMRADE scale in a sample of schizophrenia spectrum disorders". Patient Educ Couns.101(8):1477-82. | Spain | Psychiatry | 2 |
| 1090 | Wang D, Liu C, Zhang Z, Ye L, Zhang X. (2018). "Testing a healthcare provider-patient communicative relationship quality model of pharmaceutical care in hospitals". Int J Clin Pharm.40(3):617-26. | China | Pharmacy | 75, 3 |
| 1169 | Jones G, Brennan V, Jacques R, Wood H, Dixon S, Radley S. (2018). "Evaluating the impact of a 'virtual clinic' on patient experience, personal and provider costs of care in urinary incontinence: A randomised controlled trial". PLoS ONE.13(1):e0189174. | UK | Gynecology and obstetrics | 61 |
| 1204 | Mueck KM, Leal IM, Wan CC, Goldberg BF, Saunders TE, Millas SG, et al. (2018). "Shared decision-making during surgical consultation for gallstones at a safety-net hospital". Surgery.163(4):680-6. | United States | Surgery | 1 |
| 1217 | Hedberg B, Malm D, Karlsson J-E, Arestedt K, Brostrom A. (2018). "Factors associated with confidence in decision making and satisfaction with risk communication among patients with atrial fibrillation". Eur J Cardiovasc Nurs.17(5):446-55. | Sweden | Cardiovascular | 2 |
| 1254 | Homma M, Ishikawa H, Kiuchi T. (2018). "Illness perceptions and negative responses from medical professionals in patients with fibromyalgia: Association with patient satisfaction and number of hospital visits". Patient Educ Couns.101(3):532-40. | Japan | Musculoskeletal | 65 |
| 1274 | Hatton J, Chandra R, Lucius D, Ciuchta E. (2018). "Patient Satisfaction of Pharmacist-Provided Care via Clinical Video Teleconferencing". J Pharm Pract.31(5):429-33. | United States | Pharmacy | 56 |
| 1606 | Flower KB, Skinner AC, Yin HS, Rothman RL, Sanders LM, Delamater A, et al. (2017). "Satisfaction With Communication in Primary Care for Spanish-Speaking and English-Speaking Parents". Acad Pediatr.17(4):416-23. | United States | Pediatrics | 6 |
| 1746 | Mossie Chekol B, Abera Abdi D, Andualem Adal T. (2016). "Dimensions of patient satisfaction with comprehensive abortion care in Addis Ababa, Ethiopia". Reprod Health.13(1):144. | Ethiopia | Gynecology and obstetrics | 76 |
| 1747 | Hess EP, Hollander JE, Schaffer JT, Kline JA, Torres CA, Diercks DB, et al. (2016). "Shared decision making in patients with low risk chest pain: prospective randomized pragmatic trial". Bmj.355:i6165. | United States | Emergency | 8, 72 |
| 1750 | Jayachandran V, Chapotera G, Stones W. (2016). "Quality of facility-based family planning services for adolescents in Malawi: Findings from a national census of health facilities". Malawi Med J.28(2):48-52. | Malawi | Multiple settings | 39 |
| 1760 | Elmore N, Burt J, Abel G, Maratos FA, Montague J, Campbell J, et al. (2016). "Investigating the relationship between consultation length and patient experience: a cross-sectional study in primary care". Br J Gen Pract.66(653):e896-e903. | UK | Primary Care | 41 |
| 1797 | Slatore CG, Wiener RS, Golden SE, Au DH, Ganzini L. (2016). "Longitudinal Assessment of Distress among Veterans with Incidental Pulmonary Nodules". Ann Am Thorac Soc.13(11):1983-91. | United States | Pulmonology | 19 |
| 1820 | Plewnia A, Bengel J, Korner M. (2016). "Patient-centeredness and its impact on patient satisfaction and treatment outcomes in medical rehabilitation". Patient Educ Couns.99(12):2063-70. | Germany | Multiple settings | 32* |
| 1827 | Almario CV, Chey WD, Khanna D, Mosadeghi S, Ahmed S, Afghani E, et al. (2016). “Impact of National Institutes of Health Gastrointestinal PROMIS Measures in Clinical Practice: Results of a Multicenter Controlled Trial”. Am J Gastroenterol.111(11):1546-56. | United States | Gastroenterology | 3, 1, 5 |
| 1846 | Goh ML, Ang ENK, Chan Y-H, He H-G, Vehvilainen-Julkunen K. (2016). "A descriptive quantitative study on multi-ethnic patient satisfaction with nursing care measured by the Revised Humane Caring Scale". Appl Nurs Res.31:126-31. | Singapore | Multiple settings | 44 |
| 1909 | Moseson EM, Wiener RS, Golden SE, Au DH, Gorman JD, Laing AD, et al. (2016). "Patient and Clinician Characteristics Associated with Adherence. A Cohort Study of Veterans with Incidental Pulmonary Nodules". Ann Am Thorac Soc.13(5):651-9. | United States | Pulmonology | 19 |
| 1928 | van Bruinessen IR, van der Hout LE, van Weel-Baumgarten EM, Gouw H, Zijlstra JM, van Dulmen S. (2016). "Communication during haematological consultations; patients' preferences and professionals' performances". Ann Hematol.95(7):1177-83. | Netherlands | Oncology | 16 |
| 1946 | De Salins CA, Brenaut E, Misery L, Roguedas-Contios AM. (2016). "Factors influencing patient satisfaction: assessment in outpatients in dermatology department". J Eur Acad Dermatol Venereol.30(10):1823-8. | France | Dermatology | 81 |
| 1975 | Etingen B, Miskevics S, LaVela SL. (2016). "Assessing the Associations of Patient-Reported Perceptions of Patient-Centered Care as Supplemental Measures of Health Care Quality in VA". J Gen Intern Med.31 Suppl 1:10-20. | United States | Multiple settings | 2, 4, 17 |
| 1998 | Bredart A, Untas A, Copel L, Leufroy M, Mino J-C, Boiron C, et al. (2016). "Breast Cancer Survivors' Supportive Care Needs, Posttraumatic Growth and Satisfaction with Doctors' Interpersonal Skills in Relation to Physical Activity 8 Months after the End of Treatment: A Prospective Exploratory Study". Oncology.90(3):151-9. | France | Oncology | 38 |
| 2023 | Ruberton PM, Huynh HP, Miller TA, Kruse E, Chancellor J, Lyubomirsky S. (2016). "The relationship between physician humility, physician-patient communication, and patient health". Patient Educ Couns.99(7):1138-45. | United States | Primary Care | 9 |
| 2051 | Ramos KJ, Downey L, Nielsen EL, Treece PD, Shannon SE, Curtis JR, et al. (2016). "Using Nurse Ratings of Physician Communication in the ICU To Identify Potential Targets for Interventions To Improve End-of-Life Care". J Palliat Med.19(3):292-9. | United States | Palliative care | 67† |
| 2056 | Lussier M-T, Richard C, Glaser E, Roberge D. (2016). “The impact of a primary care e-communication intervention on the participation of chronic disease patients who had not reached guideline suggested treatment goals”. Patient Educ Couns.99(4):530-41. | Canada | Primary Care | 14* |
| 2093 | Lafata JE, Shay LA, Brown R, Street RL. (2016). "Office-Based Tools and Primary Care Visit Communication, Length, and Preventive Service Delivery". Health Serv Res.51(2):728-45. | United States | Primary Care | 71 |
| 2109 | Alcantara J, Ohm J, Alcantara J. (2016). "The use of PROMIS and the RAND VSQ9 in chiropractic patients receiving care with the Webster Technique". Complement Ther Clin Pract.23:110-6. | United States | Musculoskeletal | 28 |
| 2128 | Mattarozzi K, Fino E, Panni V, Agostini A, Morganti AG, Russo PM. (2019). "The Role Of Effective Radiation Therapist-Patient Communication In Alleviating Treatment-Related Pain And Procedural Discomfort During Radiotherapy". Patient Prefer Adherence.13:1861-5. | Italy | Oncology | 34 |
| 2143 | Mathur R, de Korne DF, Wong TY, Hwee DTT, Chiang PP, Wong E, et al. (2019). "Shared Care for Patients with Diabetes at Risk of Retinopathy: A Feasibility Trial". Int J Integr Care.19(3):18. | Singapore | Ophthalmology | 27 |
| 2163 | Chandra S, Ward P, Mohammadnezhad M. (2019). "Factors Associated With Patient Satisfaction in Outpatient Department of Suva Sub-divisional Health Center, Fiji, 2018: A Mixed Method Study". Front.7:183. | Fiji | Primary care | 110 |
| 2235 | van Beusekom M, Cameron J, Bedi C, Banks E, Kelsey T, Humphris G. (2018). “Development, acceptability and feasibility of a communication skills training package for therapeutic radiographers to reduce fear of recurrence development in breast cancer patients (FORECAST2)”. Pilot feasibility stud.4:148. | Scotland | Oncology | 4, 10 |
| 2343 | Morrell J, Stratman EJ. (2016). "Relationship Between Physicians' Active Participation in Maintenance of Certification and Patients' Perspective of Care Surveys". J Patient Exp.3(2):43-7. | United States | Primary care | 17 |
| 2379 | Durand M-A, Bekker HL, Casula A, Elias R, Ferraro A, Lloyd A, et al. (2016). "Can we routinely measure patient involvement in treatment decision-making in chronic kidney care? A service evaluation in 27 renal units in the UK". Clin Kidney J.9(2):252-9. | UK | Nephrology | 69 |
| 2386 | Warmington K, Kennedy CA, Lundon K, Soever LJ, Brooks SC, Passalent LA, et al. (2015). "The patient perspective: arthritis care provided by Advanced Clinician Practitioner in Arthritis Care program-trained clinicians". Open access rheumatol.7:45-53. | Canada | Musculoskeletal | 59 |
| 2620 | Plentara R, Knyszynska A, Bazydlo M, Zabielska P, Kim A, Kotwas A, et al. (2015). "[Patient satisfaction measure of the quality of primary health care]". Pomeranian J Life Sci.61(3):335-40. | Poland | Primary care | 23 |
| 2680 | Bernacki R, Hutchings M, Vick J, Smith G, Paladino J, Lipsitz S, et al. (2015). "Development of the Serious Illness Care Program: a randomised controlled trial of a palliative care communication intervention". BMJ Open.5(10):e009032. | United States | Palliative care | 15 |
| 2709 | Kambala C, Lohmann J, Mazalale J, Brenner S, De Allegri M, Muula AS, et al. (2015). "How do Malawian women rate the quality of maternal and newborn care? Experiences and perceptions of women in the central and southern regions". BMC Pregnancy Childbirth.15:169. | Malawi | Gynecology and obstetrics | 108 |
| 2754 | Aung K-K, Wu WK, Tokumi A, Kuo P, Day CS. (2015). "Does a Directive to an Internet Site Enhance the Doctor-Patient Interaction? A Prospective Randomized Study for Patients with Carpal Tunnel Syndrome". J Bone Joint Surg Am.97(13):1112-8. | Israel | Surgery | 17 |
| 2793 | LeBlanc A, Wang AT, Wyatt K, Branda ME, Shah ND, Van Houten H, et al. (2015). "Encounter Decision Aid vs. Clinical Decision Support or Usual Care to Support Patient-Centered Treatment Decisions in Osteoporosis: The Osteoporosis Choice Randomized Trial II". PLoS ONE.10(5):e0128063. | United States | Primary care | 8 |
| 2799 | Waylen A, Makoul G, Albeyatti Y. (2015). "Patient-clinician communication in a dental setting: a pilot study". Br Dent J.218(10):585-8; discussion 8. | UK | Dentistry | 6 |
| 2815 | Perez-Salgado D, Compean-Dardon MS, Staines-Orozco MG, Ortiz-Hernandez L. (2015). "Satisfaction with Healthcare Services and Adherence to Antiretroviral Therapy among Patients with HIV Attending Two Public Institutions". Rev Invest Clin.67(2):80-8. | Mexico | Immunization | 111 |
| 2830 | den Ouden H, Vos RC, Reidsma C, Rutten GEHM. (2015). "Shared decision making in type 2 diabetes with a support decision tool that takes into account clinical factors, the intensity of treatment and patient preferences: design of a cluster randomised (OPTIMAL) trial". BMC Fam Pract.16:27. | Netherlands | Endocrinology | 1 |
| 2928 | Goldzweig G, Abramovitch A, Brenner B, Perry S, Peretz T, Baider L. (2015). "Expectations and Level of Satisfaction of Patients and Their Physicians: Concordance and Discrepancies". Psychosomatics.56(5):521-9. | Israel | Oncology | 105 |
| 2951 | White RO, Eden S, Wallston KA, Kripalani S, Barto S, Shintani A, et al. (2015). "Health communication, self-care, and treatment satisfaction among low-income diabetes patients in a public health setting". Patient Educ Couns.98(2):144-9. | United States | Endocrinology | 7, 6 |
| 3013 | Dahlem CHY, Villarruel AM, Ronis DL. (2015). "African American women and prenatal care: perceptions of patient-provider interaction". West J Nurs Res.37(2):217-35. | United States | Gynecology and obstetrics | 47, 101, 106 |
| 3037 | Nadkarni GN, Sabharwal MS, Ammakkanavar NR, Annapureddy N, Malhan R, Mehta B, et al. (2014). "Patient satisfaction and resident postgraduate year status". Int J Health Care Qual Assur.27(3):182-9. | United States | Primary Care | 54 |
| 3096 | Saleh M, Almasri NA. (2014). "Use of the Measure of Processes of Care (MPOC-20) to evaluate health service delivery for children with cerebral palsy and their families in Jordan: validation of Arabic-translated version (AR-MPOC-20)". Child Care Health Dev.40(5):680-8. | Jordan | Pediatrics | 25 |
| 3183 | Street RL, Jr., Liu L, Farber NJ, Chen Y, Calvitti A, Zuest D, et al. (2014). “Provider interaction with the electronic health record: the effects on patient-centered communication in medical encounters”. Patient Educ Couns.96(3):315-9. | United States | Primary Care | 57, 11 |
| 3286 | Slatore CG, Feemster LC, Au DH, Engelberg RA, Curtis JR, Uman J, et al. (2014). "Which patient and clinician characteristics are associated with high-quality communication among veterans with chronic obstructive pulmonary disease?". J Health Commun.19(8):907-21. | United States | Pulmonology | 15 |
| 3374 | Tan ECK, Stewart K, Elliott RA, George J. (2014). "Pharmacist consultations in general practice clinics: the Pharmacists in Practice Study (PIPS)". Res Social Adm Pharm.10(4):623-32. | Australia | Primary Care | 35 |
| 3417 | Kemicer-Chmielewska E, Rotter I, Kotwas A, Koziarska D, Karakiewicz B. (2013). "[Evaluation of the quality of medical services by patients including selected sociodemographic variables]". Ann Acad Med Stetin.59(2):143-6. | Poland | Multiple settings | 131 |
| 3484 | van der Krieke L, Emerencia AC, Boonstra N, Wunderink L, de Jonge P, Sytema S. (2013). "A web-based tool to support shared decision making for people with a psychotic disorder: randomized controlled trial and process evaluation". J Med Internet Res.15(10):e216. | Netherlands | Psychiatry | 2* |
| 3534 | Sepucha K, Feibelmann S, Chang Y, Clay CF, Kearing SA, Tomek I, et al. (2013). "Factors associated with the quality of patients' surgical decisions for treatment of hip and knee osteoarthritis". J Am Coll Surg.217(4):694-701. | United States | Musculoskeletal | 77 |
| 3593 | Robyn PJ, Barnighausen T, Souares A, Savadogo G, Bicaba B, Sie A, et al. (2013). "Does enrollment status in community-based insurance lead to poorer quality of care? Evidence from Burkina Faso". Intern.12:31. | Burkina Faso | Primary Care | 60 |
| 3603 | Liang C-Y, Wang K-Y, Hwang S-J, Lin K-C, Pan H-H. (2013). "Factors affecting the physician-patient relationship of older veterans with inadequate health literacy: an observational study". Br J Gen Pract.63(610):e354-60. | Taiwan | Primary Care | 26, 11 |
| 3614 | van Weert JCM, Bolle S, van Dulmen S, Jansen J. (2013). "Older cancer patients' information and communication needs: what they want is what they get?". Patient Educ Couns.92(3):388-97. | Netherlands | Oncology | 16 |
| 3617 | Flickinger TE, Saha S, Moore RD, Beach MC. (2013). "Higher quality communication and relationships are associated with improved patient engagement in HIV care". J Acquir Immune Defic Syndr.63(3):362-6. | United States | Immunization | 100 |
| 3711 | Quaschning K, Korner M, Wirtz M. (2013). "Analyzing the effects of shared decision-making, empathy and team interaction on patient satisfaction and treatment acceptance in medical rehabilitation using a structural equation modeling approach". Patient Educ Couns.91(2):167-75. | Germany | Musculoskeletal | 1, 4, 13* |
| 3813 | Houle J, Beaulieu M-D, Lussier M-T, Del Grande C, Pellerin J-P, Authier M, et al. (2012). "Patients' experience of chronic illness care in a network of teaching settings". Can Fam Physician.58(12):1366-73. | Canada | Primary care | 14 |
| 3842 | Nembhard IM, Northrup V, Shaller D, Cleary PD. (2012). "Improving organizational climate for quality and quality of care: does membership in a collaborative help?". Med Care.50 Suppl:S74-82. | United States | Primary care | 3 |
| 3843 | Scholle SH, Vuong O, Ding L, Fry S, Gallagher P, Brown JA, et al. (2012). "Development of and field test results for the CAHPS PCMH Survey". Med Care.50 Suppl:S2-10. | United States | Primary care | 3, 104 |
| 3904 | Ortiz G, Schacht L. (2012). "Psychometric evaluation of an inpatient consumer survey measuring satisfaction with psychiatric care". Patient.5(3):163-73. | United States | Psychiatry | 45 |
| 3921 | Yanez B, Stanton AL, Maly RC. (2012). "Breast cancer treatment decision making among Latinas and non-Latina Whites: a communication model predicting decisional outcomes and quality of life". Health Psychol.31(5):552-61. | United States | Oncology | 3, 94* |
| 3956 | Mocherla S, Raman U, Holden B. (2012). "Expressions of equity: imbalances in the patient-clinician interaction". Indian J Med Ethics.9(2):87-93. | India | Ophthalmology | 112 |
| 3972 | Maben J, Adams M, Peccei R, Murrells T, Robert G. (2012). “’Poppets and parcels’: the links between staff experience of work and acutely ill older peoples’ experience of hospital care”. Int J Older People Nurs.7(2):83-94. | UK | Geriatrics | 27* |
| 4066 | Evangelista J-AK, Connor JA, Pintz C, Saia T, O'Connell C, Fulton DR, et al. (2012). "Paediatric nurse practitioner managed cardiology clinics: patient satisfaction and appointment access". J Adv Nurs.68(10):2165-74. | United States | Cardiovascular | 30 |
| 4077 | Tsui P, Day M, Thorn B, Rubin N, Alexander C, Jones R. (2012). "The communal coping model of catastrophizing: patient-health provider interactions". Pain Med.13(1):66-79. | United States | Multiple settings | 132 |
| 4082 | Phillips LA, Leventhal H, Leventhal EA. (2012). "Physicians' communication of the common-sense self-regulation model results in greater reported adherence than physicians' use of interpersonal skills". Br J Health Psychol.17(2):244-57. | United States | Primary care | 36 |
| 4122 | Sabesan S, Simcox K, Marr I. (2012). "Medical oncology clinics through videoconferencing: an acceptable telehealth model for rural patients and health workers". Intern Med J.42(7):780-5. | Australia | Oncology | 55 |
| 4144 | Hamasaki T, Soh I, Takehara T, Hagihara A. (2011). "Applicability of both dentist and patient perceptions of dentists' explanations to the evaluation of dentist-patient communication". Community Dent Health.28(4):274-9. | Japan | Dentistry | 78 |
| 4257 | Mercer SW, Fung CSC, Chan FWK, Wong FYY, Wong SYS, Murphy D. (2011). "The Chinese-version of the CARE measure reliably differentiates between doctors in primary care: a cross-sectional study in Hong Kong". BMC Fam Pract.12:43. | Hong Kong | Primary care | 4 |
| 4266 | Montori VM, Shah ND, Pencille LJ, Branda ME, Van Houten HK, Swiglo BA, et al. (2011). "Use of a decision aid to improve treatment decisions in osteoporosis: the osteoporosis choice randomized trial". Am J Med.124(6):549-56. | United States | Primary care | 8 |
| 4400 | Burford B, Greco M, Bedi A, Kergon C, Morrow G, Livingston M, et al. (2011). "Does questionnaire-based patient feedback reflect the important qualities of clinical consultations? Context, benefits and risks". Patient Educ Couns.84(2):e28-36. | UK | Primary care | 5 |
| 4470 | Busato A, Kunzi B. (2010). "Differences in the quality of interpersonal care in complementary and conventional medicine". BMC Altern Med.10:63. | Switzerland | Primary care | 23 |
| 4512 | van Empel IWH, Aarts JWM, Cohlen BJ, Huppelschoten DA, Laven JSE, Nelen WLDM, et al. (2010). "Measuring patient-centredness, the neglected outcome in fertility care: a random multicentre validation study". Hum Reprod.25(10):2516-26. | Netherlands | Gynecology and obstetrics | 58 |
| 4553 | Kripalani S, Jacobson TA, Mugalla IC, Cawthon CR, Niesner KJ, Vaccarino V. (2010). "Health literacy and the quality of physician-patient communication during hospitalization". J Hosp Med.5(5):269-75. | United States | Cardiovascular | 7 |
| 4614 | Slatore CG, Cecere LM, Reinke LF, Ganzini L, Udris EM, Moss BR, et al. (2010). "Patient-clinician communication: associations with important health outcomes among veterans with COPD". Chest.138(3):628-34. | United States | Palliative care | 15 |
| 4626 | Bieber C, Muller KG, Nicolai J, Hartmann M, Eich W. (2010). "How does your doctor talk with you? Preliminary validation of a brief patient self-report questionnaire on the quality of physician-patient interaction". J Clin Psychol Med Settings.17(2):125-36. | Germany | Multiple settings | 66* |
| 4672 | Stewart RF, Kroth PJ, Schuyler M, Bailey R. (2010). "Do electronic health records affect the patient-psychiatrist relationship? A before & after study of psychiatric outpatients". BMC Psychiatry.10:3. | United States | Psychiatry | 9 |
| 4703 | Halpert A, Dalton CB, Palsson O, Morris C, Hu Y, Bangdiwala S, et al. (2010). "Irritable bowel syndrome patients' ideal expectations and recent experiences with healthcare providers: a national survey". Dig Dis Sci.55(2):375-83. | United States | Multiple settings | 107 |
| 4733 | Agha Z, Schapira RM, Laud PW, McNutt G, Roter DL. (2009). "Patient satisfaction with physician-patient communication during telemedicine". Telemed J E Health.15(9):830-9. | United States | Internal medicine | 52 |
| 4783 | Zantinge EM, Verhaak PFM, de Bakker DH, van der Meer K, Bensing JM. (2009). "Does burnout among doctors affect their involvement in patients' mental health problems? A study of videotaped consultations". BMC Fam Pract.10:60. | Netherlands | Primary care | 95 |
| 4802 | van Weert JCM, Jansen J, de Bruijn G-J, Noordman J, van Dulmen S, Bensing JM. (2009). "QUOTEchemo: a patient-centred instrument to measure quality of communication preceding chemotherapy treatment through the patient's eyes". Eur J Cancer.45(17):2967-76. | Netherlands | Oncology | 16 |
| 4836 | Moseley KL, Hudson EJ. (2009). "Steroid inhaler adherence, flu vaccine receipt, and race: associations with the quality of the parent-physician relationship for asthmatic children". J Natl Med Assoc.101(5):407-13. | United States | Pediatrics | 133 |
| 4891 | Tung Y-C, Chang G-M. (2009). "Patient satisfaction with and recommendation of a primary care provider: associations of perceived quality and patient education". Int J Qual Health Care.21(3):206-13. | Taiwan | Primary care | 134 |
| 4905 | Vodermaier A, Caspari C, Koehm J, Kahlert S, Ditsch N, Untch M. (2009). "Contextual factors in shared decision making: a randomised controlled trial in women with a strong suspicion of breast cancer". Br J Cancer.100(4):590-7. | Germany | Gynecology and obstetrics | 11, 13 |
| 4910 | Dierick-van Daele ATM, Metsemakers JFM, Derckx EWCC, Spreeuwenberg C, Vrijhoef HJM. (2009). "Nurse practitioners substituting for general practitioners: randomized controlled trial". J Adv Nurs.65(2):391-401. | Netherlands | Primary care | 199 |
| 5001 | Jabaaij L, Fassaert T, van Dulmen S, Timmermans A, van Essen GA, Schellevis F. (2008). "Familiarity between patient and general practitioner does not influence the content of the consultation". BMC Fam Pract.9:51. | Netherlands | Primary care | 82 |
| 5028 | Moran J, Bekker H, Latchford G. (2008). “Everyday use of patient-centred, motivational techniques in routine consultations between doctors and patients with diabetes”. Patient Educ Couns.73(2):224-31. | UK | Endocrinology | 37, 22, 31 |
| 5089 | Krones T, Keller H, Sonnichsen A, Sadowski E-M, Baum E, Wegscheider K, et al. (2008). "Absolute cardiovascular disease risk and shared decision making in primary care: a randomized controlled trial". Ann Fam Med.6(3):218-27. | Germany | Primary care | 13, 1 |
| 5115 | Levinson W, Hudak PL, Feldman JJ, Frankel RM, Kuby A, Bereknyei S, et al. (2008). ""It's not what you say ...": racial disparities in communication between orthopedic surgeons and patients". Med Care.46(4):410-6. | Canada | Musculoskeletal | 102, 135, 96 |
| 5495 | Bergenmar M, Nylen U, Lidbrink E, Bergh J, Brandberg Y. (2006). "Improvements in patient satisfaction at an outpatient clinic for patients with breast cancer". Acta Oncol.45(5):550-8. | Sweden | Oncology | 80 |
| 5505 | Smith MY, Winkel G, Egert J, Diaz-Wionczek M, DuHamel KN. (2006). "Patient-physician communication in the context of persistent pain: validation of a modified version of the patients' Perceived Involvement in Care Scale". J Pain Symptom Manage.32(1):71-81. | United States | Oncology | 49, 9 |
| 5715 | Nayak S, Pradhan JPB, Reddy S, Palmer JL, Zhang T, Bruera E. (2005). "Cancer patients' perception of the quality of communication before and after the implementation of a communication strategy in a regional cancer center in India". J Clin Oncol.23(21):4771-5. | India | Oncology | 83 |
| 5817 | Kindler CH, Szirt L, Sommer D, Hausler R, Langewitz W. (2005). "A quantitative analysis of anaesthetist-patient communication during the pre-operative visit". Anaesthesia.60(1):53-9. | Switzerland | Anesthetics | 8* |
| 5864 | Hurst YK, Prescott-Clements LE, Rennie JS. (2004). "The patient assessment questionnaire: a new instrument for evaluating the interpersonal skills of vocational dental practitioners". Br Dent J.197(8):497-500. | UK | Dentistry | 53 |
| 5917 | Wood J, Collins J, Burnside ES, Albanese MA, Propeck PA, Kelcz F, et al. (2004). "Patient, faculty, and self-assessment of radiology resident performance: a 360-degree method of measuring professionalism and interpersonal/communication skills". Acad Radiol.11(8):931-9. | United States | Interventional radiology | 93 |
| 5988 | Alkazaleh F, Thomas M, Grebenyuk J, Glaude L, Savage D, Johannesen J, et al. (2004). "What women want: women's preferences of caregiver behavior when prenatal sonography findings are abnormal". Ultrasound Obstet Gynecol.23(1):56-62. | Canada | Gynecology and obstetrics | 84 |
| 6028 | Cooper LA, Roter DL, Johnson RL, Ford DE, Steinwachs DM, Powe NR. (2003). "Patient-centered communication, ratings of care, and concordance of patient and physician race". Ann Intern Med.139(11):907-15. | United States | Primary care | 103* |
| 6084 | Street RL, Jr., Krupat E, Bell RA, Kravitz RL, Haidet P. (2003). "Beliefs about control in the physician-patient relationship: effect on communication in medical encounters". J Gen Intern Med.18(8):609-16. | United States | Multiple settings | 22 |
| 6086 | Edwards A, Elwyn G, Hood K, Robling M, Atwell C, Holmes-Rovner M, et al. (2003). "The development of COMRADE--a patient-based outcome measure to evaluate the effectiveness of risk communication and treatment decision making in consultations". Patient Educ Couns.50(3):311-22. | UK | Primary care | 2 |
| 6159 | Pinnock H, Bawden R, Proctor S, Wolfe S, Scullion J, Price D, et al. (2003). "Accessibility, acceptability, and effectiveness in primary care of routine telephone review of asthma: pragmatic, randomised controlled trial". Bmj.326(7387):477-9. | UK | Primary care | 51 |
| 6187 | Ibrahim EM, Al-Saad R, Wishi AL, Khafaga YM, El Hussainy G, Nabhan A, et al. (2002). "Appraisal of communication skills and patients' satisfaction in cross-language encounters in oncology practice". J Cancer Educ.17(4):216-21. | Saudi Arabia | Oncology | 97 |
| 6212 | Golin C, DiMatteo MR, Duan N, Leake B, Gelberg L. (2002). "Impoverished diabetic patients whose doctors facilitate their participation in medical decision making are more satisfied with their care". J Gen Intern Med.17(11):857-66. | United States | Endocrinology | 114, 40 |
| 6294 | Heisler M, Bouknight RR, Hayward RA, Smith DM, Kerr EA. (2002). "The relative importance of physician communication, participatory decision making, and patient understanding in diabetes self-management". J Gen Intern Med.17(4):243-52. | United States | Endocrinology | 136 |
| 6299 | Greco M, Spike N, Powell R, Brownlea A. (2002). "Assessing communication skills of GP registrars: a comparison of patient and GP examiner ratings". Med Educ.36(4):366-76. | UK | Primary care | 5 |
| 6357 | Renzi C, Abeni D, Picardi A, Agostini E, Melchi CF, Pasquini P, et al. (2001). "Factors associated with patient satisfaction with care among dermatological outpatients". Br J Dermatol.145(4):617-23. | Italy | Dermatology | 98 |
| 6389 | Greco M, Brownlea A, McGovern J. (2001). "Impact of patient feedback on the interpersonal skills of general practice registrars: results of a longitudinal study". Med Educ.35(8):748-56. | UK | Primary care | 5 |
| 6449 | Peck BM, Asch DA, Goold SD, Roter DL, Ubel PA, McIntyre LM, et al. (2001). "Measuring patient expectations: does the instrument affect satisfaction or expectations?". Med Care.39(1):100-8. | United States | Primary care | 18, 28 |
| 6548 | Medalie JH, Zyzanski SJ, Goodwin MA, Stange KC. (2000). "Two physician styles of focusing on the family". Journal of Family Practice.49(3):209-15. | United States | Primary care | 139 |
| 6560 | Haddad S, Potvin L, Roberge D, Pineault R, Remondin M. (2000). "Patient perception of quality following a visit to a doctor in a primary care unit". Fam Pract.17(1):21-9. | Canada | Primary care | 79 |
| 6601 | Loblaw DA, Bezjak A, Bunston T. (1999). "Development and testing of a visit-specific patient satisfaction questionnaire: the Princess Margaret Hospital Satisfaction With Doctor Questionnaire". J Clin Oncol.17(6):1931-8. | Canada | Oncology | 62 |
| 6715 | Flocke SA, Stange KC, Zyzanski SJ. (1998). "The association of attributes of primary care with the delivery of clinical preventive services". Med Care.36(8 Suppl):AS21-30. | United States | Primary care | 21 |
| 6747 | Greco M, Francis W, Buckley J, Brownlea A, McGovern J. (1998). "Real-patient evaluation of communication skills teaching for GP registrars". Fam Pract.15(1):51-7. | UK | Primary care | 5 |
| 6802 | Flocke SA, Stange KC, Zyzanski SJ. (1997). "The impact of insurance type and forced discontinuity on the delivery of primary care". Journal of Family Practice.45(2):129-35. | United States | Primary care | 21 |
| 6944 | Butow PN, Dunn SM, Tattersall MH, Jones QJ. (1995). "Computer-based interaction analysis of the cancer consultation". Br J Cancer.71(5):1115-21. | Australia | Oncology | 113 |
| 7368 | Shiozawa T, Yamaguchi S, Matsunaga A, Sawada U, Fujii C. (2020). "Development of the Interpersonal Processes of Care Survey-Japanese version". Neuropsychopharmacology Reports.40(1):107-12. | Japan | Psychiatry | 7*† |
| 7425 | Radder DLM, Lennaerts HH, Vermeulen H, Van Asseldonk T, Delnooz CCS, Hagen RH, et al. (2020). "The cost-effectiveness of specialized nursing interventions for people with Parkinson's disease: The NICE-PD study protocol for a randomized controlled clinical trial". Trials.21 (1) (no pagination)(88). | Netherlands | Neurology | 20 |
| 7788 | Kakouei M, Mohtashami J, Tafreshi MZ, Qoli MAP. (2018). "Auditing the compliance with the principles of nurse - Patient communication at psychiatric wards of Shahid Beheshti University of medical sciences in 2014". Annals of Tropical Medicine and Public Health.5(Special Issue):S201. | Iran | Psychiatry | 85 |
| 7834 | Amin ZA, Kabir MI, Karami JH, Nahar N. (2018). "Doctor-patient communication to improve adherence to anti-hypertensive treatment". Bangladesh Med Res Counc Bull.44(3):145-51. | Bangladesh | Cardiovascular | 6 |
| 7905 | Bove R, Garcha P, Bevan CJ, Crabtree-Hartman E, Green AJ, Gelfand JM. (2018). "Clinic to in-home telemedicine reduces barriers to care for patients with MS or other neuroimmunologic conditions". Neurology: Neuroimmunology and NeuroInflammation.5 (6) (no pagination)(e505). | United States | Neurology | 86 |
| 8119 | Alanazi MR, Alamry A, Al-Surimi K. (2017). "Validation and adaptation of the hospital consumer assessment of healthcare providers and systems in Arabic context: Evidence from Saudi Arabia". Journal of Infection and Public Health.10(6):861-5. | Saudi Arabia | Multiple settings | 12 |
| 8126 | Eton DT, Ridgeway JL, Linzer M, Boehm DH, Rogers EA, Yost KJ, et al. (2017). “Healthcare provider relational quality is associated with better self-management and less treatment burden in people with multiple chronic conditions”. Patient Preference and Adherence.11:1635-46. | United States | Primary Care | 42† |
| 8227 | Korn RE, Shukla AW, Katz M, Keenan HT, Goldenthal S, Auinger P, et al. (2017). "Virtual visits for Parkinson disease: A multicenter noncontrolled cohort". Neurology: Clinical Practice.7(4):283-95. | United States | Neurology | † |
| 8266 | Jager M, Reijneveld SA, Almansa J, Metselaar J, Knorth EJ, De Winter AF. (2017). "Less reduction of psychosocial problems among adolescents with unmet communication needs". European Child and Adolescent Psychiatry.26(4):403-12. | Netherlands | Psychiatry | 20 |
| 8471 | Hu W, Song Y, Zhong X, Feng J, Wang P, Huang C. (2016). "Improving doctor-patient communication: Content validity examination of a novel urinary system-simulating physical model". Patient Preference and Adherence.10:2519-29. | China | Urology | 10 |
| 8764 | Harmsen CG, Kristiansen IS, Larsen PV, Nexoe J, Stovring H, Gyrd-Hansen D, et al. (2014). "Communicating risk using absolute risk reduction or prolongation of life formats: Cluster-randomisedtrial in general practice". Br J Gen Pract.64(621):e199-e207. | Denmark | Primary Care | 2 |
| 8802 | Li X, Zhang H, Wang J, Li F, Chen J. (2014). "Assessing patient satisfaction with medication-related services in hospital settings: A cross-sectional questionnaire survey in China". International Journal of Clinical Pharmacology and Therapeutics.52(7):587-97. | China | Multiple settings | 87 |
| 8960 | So S, Rogers A, Patterson C, Drew W, Maxwell J, Darch J, et al. (2014). "Parental experiences of a developmentally focused care program for infants and children during prolonged hospitalization". Journal of child health care : for professionals working with children in the hospital and community.18(2):156-67. | Canada | Pediatrics | 25 |
| 9061 | Braeken APBM, Lechner L, Eekers DBP, Houben RMA, van Gils FCJM, Ambergen T, et al. (2013). "Does routine psychosocial screening improve referral to psychosocial care providers and patient-radiotherapist communication? A cluster randomized controlled trial". Patient Education and Counseling.93(2):289-97. | Netherlands | Oncology | 88 |
| 9306 | Kumar S, Haque A, Tehrani HY. (2012). "High satisfaction rating by users of private-for-profit healthcare providers-evidence from a cross-sectional survey among inpatients of a private tertiary level hospital of north India". North American Journal of Medical Sciences.4(9):405-10. | India | Multiple settings | 89 |
| 9520 | Abioye Kuteyi EA, Bello IS, Olaleye TM, Ayeni IO, Amedi MI. (2010). "Determinants of patient satisfaction with physician interaction: A cross-sectional survey at the Obafemi Awolowo University Health Centre, Ile-Ife, Nigeria". South African Family Practice.52(6):557-62. | Nigeria | Multiple settings | 48 |
| 9903 | Mercer SW, Murphy DJ. (2008). "Validity and reliability of the CARE Measure in secondary care". Clinical Governance.13(4):269-83. | UK | Multiple settings | 4 |
| 10094 | Matlow AG, Wishen A, Read SE, Raboud JM. (2006). "A study of provider-caregiver communication in paediatric ambulatory care". Paediatrics and Child Health.11(4):217-21. | Canada | Pediatrics | † |
| 10095 | Stapleton RD, Engelberg RA, Wenrich MD, Goss CH, Curtis JR. (2006). "Clinician statements and family satisfaction with family conferences in the intensive care unit". Crit Care Med.34(6):1679-85. | United States | Intensive Care | 90 |
| 10176 | Hart CN, Drotar D, Gori A, Lewin L. (2006). “Enhancing parent-provider communication in ambulatory pediatric practice”. Patient Education and Counseling.63(1-2):38-46. | United States | Pediatrics | 139 |
| 10360 | Greco M, Carter M, Powell R, Sweeney K, Stead J. (2004). "Does a patient survey make a difference?". Educ.15(2):183-9. | UK | Primary Care | 24 |
| 10463 | Greco M, Powell R, Sweeney K. (2003). "The Improving Practice Questionnaire (IPQ): A practical tool for general practices seeking patient views". Educ.14(4):440-8. | UK | Primary Care | 24 |
| 10470 | Piette JD, Schillinger D, Potter MB, Heisler M. (2003). "Dimensions of patient-provider communication and diabetes self-care in an ethnically diverse population". J Gen Intern Med.18(8):624-33. | United States | Endocrinology | 7 |
| 10774 | Greco M, Cavanagh M, Brownlea A, McGovern J. (1999). "The Doctors' Interpersonal Skills Questionnaire (DISQ): A validated instrument for use in GP training". Education for General Practice.10(3):256-64. | UK | Primary Care | 5 |
| 11006 | Jarman H, Sellick K. (1992). "Interpersonal communication in family planning: A comparison of client and nurse satisfaction". British Journal of Family Planning.18(1):6-8. | Australia | Gynecology and obstetrics | 48 |
| 11115 | Kenny DT. (1995). "Determinants of patient satisfaction with the medical consultation". Psychol Health.10(5):427-37. | Australia | Multiple settings | 91 |
| 11184 | Li HZ, Lundgren J. (2005). "Training patients to ask information verifying questions in medical interviews". Health Educ.105(6):451-66. | Canada | Primary Care | 92 |
| 11227 | Finderup J, Jensen JKD, Lomborg K. (2018). "Developing and pilot testing a shared decision-making intervention for dialysis choice". J.44(3):152-61. | Denmark | Nephrology | 1† |
| 11255 | Basile M, Andrews J, Jacome S, Zhang M, Kozikowski A, Hajizadeh N. (2018). "A decision aid to support shared decision making about mechanical ventilation in severe chronic obstructive pulmonary disease patients (InformedTogether): Feasibility study". J Med Internet Res.20(5). | United States | Pulmonology | 2, 46, 8† |
| 11414 | Samohýl M, Nádaždyová A, Hirjak M, Hirošová K, Vondrová D, Krajčová D, et al. (2016). "The satisfaction with health care quality in dental clinics in the Slovak Republic". Kontakt.18(1):e49-e54. | Slovak Republic | Dentistry | 132 |
| 11583 | Othman Z, Abas MA, Latif RA, Ya'acob A, Mohammad WNW, Nor'azman N. (2019). "Patients-next of kins' perceptions on information clarity and interpersonal relations in communication in a medical practice". Geografia-Malays.15(1):98-112. | Malaysia | Primary care | 63 |
| 11846 | Zakaria M, Karim R, Rahman M, Cheng F, Xu J. (2021). "Disparity in physician-patient communication by ethnicity: evidence from Bangladesh". Intern.20(1):65. | China | Primary care | 119 |
| 11850 | Yuan N, Boscardin C, Lisha NE, Dudley RA, Lin GA. (2021). "Is Better Patient Knowledge Associated with Different Treatment Preferences? A Survey of Patients with Stable Coronary Artery Disease". Patient Prefer Adherence.15:119-26. | United States | Cardiovascular | 115 |
| 11915 | Versluijs Y, Moore MG, Ring D, Jayakumar P. (2021). "Clinician Facial Expression of Emotion Corresponds with Patient Mindset". Clin Orthop.479(9):1914-23. | United States | Musculoskeletal | 26 |
| 11916 | Versluijs Y, Lemmers M, Brown LE, Gonzalez AI, Kortlever JTP, Ring D. (2021). "The Correlation of Communication Effectiveness and Patient Satisfaction". J Patient Exp.8:2374373521998839. | United States | Musculoskeletal | 117 |
| 11918 | van Hoorn BT, van Rossenberg LX, Jacobs X, Sulkers GSI, van Heijl M, Ring D. (2021). "Clinician Factors Rather Than Patient Factors Affect Discussion of Treatment Options". Clin Orthop.479(7):1506-16. | Netherlands | Surgery | 116 |
| 11931 | Tran TB, Raoof M, Melstrom L, Kyulo N, Shaikh Z, Jones VC, et al. (2021). “Racial and Ethnic Bias Impact Perceptions of Surgeon Communication”. Ann Surg.274(4):597-604. | United States | Surgery | † |
| 11932 | Torrecilla-Olavarrieta R, Perez-Revuelta J, Garcia-Spinola E, Lopez Martin A, Mongil-SanJuan JM, Rodriguez-Gomez C, et al. (2021). “Satisfaction with antipsychotics as a medication: the role of therapeutic alliance and patient-perceived participation in decision making in patients with schizophrenia spectrum disorder”. Int.25(3):268-76. | Spain | Psychiatry | 2 |
| 12024 | Rottele N, Schlett C, Korner M, Farin-Glattacker E, Schopf-Lazzarino AC, Voigt-Radloff S, et al. (2021). “Variance components of ratings of physician-patient communication: A generalizability theory analysis”. PLoS ONE.16(6):e0252968. | Germany | Primary care | 1, 118 |
| 12063 | Raina R, Nair N, Yap HK, Filler G, Sethi SK, Bagga A, et al. (2021). "Survey of Telemedicine by Pediatric Nephrologists During the COVID-19 Pandemic". KI Rep.6(9):2316-22. | United States and Canada | Pediatrics | 12 |
| 12064 | Rabah NM, Khan HA, Winkelman RD, Levin JM, Mroz TE, Steinmetz MP. (2021). "Key drivers of patient satisfaction with spine surgeons in the outpatient setting". J Neurosurg Spine.1-8. | United States | Surgery | † |
| 12106 | Parast L, Mathews M, Martino S, Lehrman WG, Stark D, Elliott MN. (2021). "Racial/Ethnic Differences in Emergency Department Utilization and Experience". J Gen Intern Med.05:05. | United States | Emergency | 3 |
| 12183 | Miller PA, Burgoon ML, Hoover-Hankerson B, Strand N, Ross H. (2021). "Utilizing Oral Surveys to Better Understand Patient Satisfaction in a Low-Income, Urban Surgical Clinic". Am Surg.87(8):1267-74. | United States | Surgery | 120 |
| 12203 | Marzo RR, Bhattacharya S, Ujang NB, Naing TW, Huong Fei AT, Chun CK, et al. (2021). "The impact of service quality provided by health-care centers and physicians on patient satisfaction". J.10:160. | Malaysia | Multiple settings | 121 |
| 12233 | Lin OM, Reid HW, Fabbro RL, Johnson KS, Batch BC, Olsen MK, et al. (2021). "Association of Provider Perspectives on Race and Racial Health Care Disparities with Patient Perceptions of Care and Health Outcomes". Health Equity.5(1):466-75. | United States | Endocrinology | 7 |
| 12246 | Lewis JJ, Balaji L, Grossestreuer AV, Ullman E, Rosen C, Dubosh NM. (2021). "Correlation of attending and patient assessment of resident communication skills in the emergency department". AEM educ.5(4):e10629. | United States | Emergency | 6 |
| 12273 | Kunneman M, Branda ME, Ridgeway JL, Tiedje K, May CR, Linzer M, et al. (2021). "Making sense of diabetes medication decisions: a mixed methods cluster randomized trial using a conversation aid intervention". Endocrine.09:09. | United States | Endocrinology | 8 |
| 12303 | Kennedy DL, Olsen MK, Yang H, Gao X, Alkon A, Prose NS, et al. (2021). "Communication Coaching in Cardiology (CCC): A study protocol and methodological challenges and solutions of a randomized controlled trial in outpatient cardiology clinics". Contemp Clin Trials.105:106389. | United States | Cardiovascular | 7, 4 |
| 12341 | Itamura K, Tang DM, Higgins TS, Rimell FL, Illing EA, Ting JY, et al. (2021). "Comparison of Patient Satisfaction Between Virtual Visits During the COVID-19 Pandemic and In-person Visits Pre-pandemic". Ann Otol Rhinol Laryngol.130(7):810-7. | United States | Otorhinolaryngology | † |
| 12363 | Hossain S, Sripad P, Zieman B, Roy S, Kennedy S, Hossain I, et al. (2021). "Measuring quality of care at the community level using the contraceptive method information index plus and client reported experience metrics in Bangladesh". J.11:07007. | Bangladesh | Multiple settings | 122, 123 |
| 12365 | Hompashe DM, Gerdtham U-G, Christian CS, Smith A, Burger R. (2021). "'The nurse did not even greet me': how informed versus non-informed patients evaluate health systems responsiveness in South Africa". BMJ Glob Health.6(4):04. | South Africa | Primary care | 124 |
| 12383 | Haskard-Zolnierek K, Martin LR, Bueno EH, Kruglikova-Sanchez Y. (2021). "Physician-Patient Communication and Satisfaction in Spanish-Language Primary Care Visits". Health Commun.1-7. | United States | Primary care | 125, 140, 141, 142, 29 |
| 12453 | Ferro-Lopez L, Barnett N, Minshull J. (2021). "Improving shared decision-making in pharmacist-led haematology clinics: a 'Plan Do Study Act' approach". Eur.26:26. | UK | Hematology | 126 |
| 12573 | Chen C-H, Kang Y-N, Chiu P-Y, Huang Y-J, Elwyn G, Wu M-H, et al. (2021). “Effectiveness of shared decision-making intervention in patients with lumbar degenerative diseases: A randomized controlled trial”. Patient Educ Couns.10:10. | Taiwan | Musculoskeletal | 1 |
| 12575 | Chang M, Russo GS, Canseco JA, Nicholson K, Sharma R, Koomson J, et al. (2021). “Variations in Patient Satisfaction Scores Between HCAHPS and a Novel Orthopedic Practice-Specific Survey”. Am J Med Qual.36(2):103-9. | United States | Musculoskeletal | 12, 127 |
| 12789 | Valdes K, Kannas S, Kakar S, Veneziano J, Dake T, Sierra F. (2020). "Patient satisfaction of hand therapy services". J Hand Ther.01:01. | United States | Musculoskeletal | 9 |
| 13097 | Kim MG, Lee NE, Sohn HS. (2020). "Gap between patient expectation and perception during pharmacist-patient communication at community pharmacy". Int J Clin Pharm.42(2):677-84. | South Korea | Pharmacy | 128 |
| 13134 | Johnson W, Ngo N-A, Elrod M. (2020). "Communication Skills of Grandview/Southview Medical Center General Surgery Residents". J Am Osteopath Assoc.120(12):865-70. | United States | Surgery | 6 |
| 13233 | Gadeka DD, Esena RK. (2020). "Quality of Care of Medical Imaging Services at a Teaching Hospital in Ghana: Clients' Perspective". J Med Imaging Radiat Sci.51(1):154-64. | Ghana | Imaging | 129, 130 |
| 13446 | Al-Hemiary NJ, Cucchi A, Al-Nuaimi AS, Al-Saffar H, Al-Ani K. (2020). "Inter-personal versus content: assessment of communication skills in Iraqi physicians". Heliyon.6(10):e05145. | Iraq | Multiple settings | 18 |
| 13451 | Ahmad E, Itrat M. (2020). "Patient Satisfaction With Medical Services Provided at Unani Medicine Hospital, Bengaluru: A Cross-Sectional Study". J Patient Exp.7(6):1432-7. | India | Multiple settings | 9 |

Notes: *included at least one unavailable instrument (see also Appendix C) ; † included at least one instrument of which all items were excluded at the ‘eligible item’ phase in the analysis.

**Appendix E.** Identified instruments

| **Instrument ID** | **Instrument Abbreviation** | **Instrument**  **Name or construct** | **Explicitly mentioned: self-developed** | **# Items included in review** | **Total N identified** | **Total Ref IDs in which instrument was identified** (bold=reference from which instrument was extracted) |
| --- | --- | --- | --- | --- | --- | --- |
| 1 | SDM-Q-9 | 9-item Shared Decision Making Questionnaire |  | 9 | 9 | 958, 1204, 1827, 2830, **3711**, 5089, 11227, 12024, 12573 |
| 2 | COMRADE | Combined Outcome Measure for Risk Communication and Treatment Decision Making Effectiveness |  | 8 | 8 | 1088, 1217, **1975**, 3484, 6086, 8764, 11255, 11932 |
| 3 | CAHPS | Consumer Assessment of Healthcare Providers and Systems |  | 7 | 7 | 311, 1090, 1827, **3842**, 3843, 3921, 12106 |
| 4 | CARE | Consultation and Relational Empathy |  | 10 | 7 | 649, **1975**, 2235, 3711, 4257, 9903, 12303 |
| 5 | DISQ | Doctors’ Interpersonal Skills Questionnaire |  | 8 | 7 | 767, 1827, 4400, **6299**, 6389, 6747, 10774 |
| 6 | CAT | Communication Assessment Tool |  | 14 | 6 | **1606**, 2799, 2951, 7834, 12246, 13134 |
| 7 | IPC | Interpersonal Processes of Care |  | 26 | 6 | 2951, **4553**, 7368, 10470, 12233, 12303 |
| 8 | OPTION-12 | Observing patient involvement in decision making |  | 12 | 6 | **1747**, 2793, 4266, 5817, 11255, 12273 |
| 9 | PSQ | Patient Satisfaction Questionnaire |  | 9 | 5 | **4672**, 5505, 11414, 12789, 13451 |
| 10 | MISS-21 | Medical Interview Satisfaction Scale |  | 11 | 4 | **559**, 1081, 2235, 11006 |
| 11 | PICS | Perceived Involvement in Care Scale |  | 11 | 4 | 311, 3603, **3183**, 4905, |
| 12 | HCAHPS | Hospital Consumer Assessment of Healthcare Providers and Systems |  | 9 | 3 | **8119**, 12063, 12575 |
| 13 |  | Man-Son-Hing Patient Satisfaction Scale |  | 5 | 3 | **3711**, 4905, 5089 |
| 14 | PACIC | Patient Assessment of Chronic Illness Care |  | 17 | 3 | 311, **2056**, 3813 |
| 15 |  | Quality of Communication with clinician |  | 17 | 3 | **2680**, 3286, 4614 |
| 16 | QUOTE chemo performance | Quality Of care Through the patients’ Eyes |  | 56 | 3 | 1928, 3614, **4802** |
| 17 |  | Press Ganey Care Provider Patient |  | 8 | 3 | **2343**, 2754, 1975 |
| 18 | ABIM | American Board of Internal Medicine |  | 9 | 2 | **6449**, 13446 |
| 19 | CCM | Consultation Care Measure |  | 16 | 2 | 1797, **1909** |
| 20 |  | Consumer Quality Index |  | 6 | 2 | 7425, **8266** |
| 21 | CPCI | Components of Primary Care Instrument |  | 6 | 2 | **6715**, 6802 |
| 22 | DCIC | Diabetes Consultation Interaction Coding (adapted from Street) |  | 8 | 2 | **5028**, 6084 |
| 23 | EUROPEP | European Task Force on Patient Evaluations of General Practice Care |  | 13 | 2 | 2620, **4470** |
| 24 | IPQ | Improving Practice Questionnaire |  | 10 | 2 | **10360**, 10463 |
| 25 | MPOC-20 | Measure of Processes of Care |  | 18 | 2 | **3096**, 8960 |
| 26 | PDRQ | Patient-Doctor Relationship Questionnaire |  | 7 | 2 | **3603**, 11915 |
| 27 |  | Picker survey (selected items) |  | 1 | 2 | **2143**, 3972 |
| 28 | RAND VSQ9 | Research and Development Visit-specific Satisfaction questionnaire |  | 3 | 2 | **2109**, 6449 |
| 29 |  | Physician Satisfaction Questionnaire |  | 8 |  | **2023**, 12383 |
| 30 |  | Ambulatory Patient Satisfaction Survey |  | 4 | 1 | **4066** |
| 31 | BECCI | Behaviour Change Counselling Index |  | 11 | 1 | **5028** |
| 32 | CCRQ-15 | Client-Centered Rehabilitation Questionnaire 15 |  | 9 | 1 | **1820** |
| 33 |  | CollaboRATE |  | 3 | 1 | **196** |
| 34 |  | Communication with Radiation Therapist - Shortened |  | 6 | 1 | **2128** |
| 35 |  | Consultation Satisfaction Questionnaire |  | 7 | 1 | **3374** |
| 36 | CS-SRM | Common-Sense Self-Regulation Model |  | 6 | 1 | **4082** |
| 37 | DISS | Diabetes interview satisfaction scale |  | 10 | 1 | **5028** |
| 38 | EORTC in-patient satisfaction questionnaire | European Organisation for Research and Treatment of Cancer (doctor's interpersonal skills subscale) |  | 3 | 1 | **1998** |
| 39 |  | Experience of Care |  | 2 | 1 | **1750** |
| 40 |  | Facilitation of patient involvement in care scale |  | 8 | 1 | **6212** |
| 41 |  | Communication (Based on General Practice Patient Survey) | Yes | 7 | 1 | **1760** |
| 42 |  | Healthcare Provider Relational Quality |  | 5 | 1 | **8126** |
| 43 |  | Human Connection Scale |  | 4 | 1 | **73** |
| 44 |  | Humane Caring Scale (Revised) |  | 21 | 1 | **1846** |
| 45 | ICS | Inpatient Consumer Survey |  | 9 | 1 | **3904** |
| 46 | MCCS | Medical Communication Competency Scale |  | 14 | 1 | **11255** |
| 47 | MCI | Matched-Pair Communication Instrument |  | 18 | 1 | **3013** |
| 48 | MISS | Medical Interview Satisfaction Scale (Adapted for British General Practice) |  | 14 | 1 | **9520** |
| 49 | M-PICS | Modified Version of the Patients’ Perceived Involvement in Care Scale |  | 19 | 1 | **5505** |
| 50 |  | National GP Patient Survey |  | 6 | 1 | **679** |
| 51 |  | Nursing care satisfaction questionnaire |  | 10 | 1 | **6159** |
| 52 | PACT | Patient Assessment of Communication during Telemedicine |  | 27 | 1 | **4733** |
| 53 | PAQ | Patient assessment questionnaire |  | 13 | 1 | **5864** |
| 54 |  | Patient Satisfaction |  | 10 | 1 | **3037** |
| 55 |  | Patient satisfaction survey |  | 7 | 1 | **4122** |
| 56 |  | Patient Satisfaction with Physician-Patient Communication During Telemedicine (abbreviated 10 item) |  | 7 | 1 | **1274** |
| 57 | PCC | Patient Centered Communication |  | 9 | 1 | **3183** |
| 58 | PCQ-infertility | Patient-centredness questionnaire-infertility |  | 20 | 1 | **4512** |
| 59 | PDIS Adapted | Patient–Doctor Interaction Scale |  | 8 | 1 | **2386** |
| 60 |  | Perceived quality of health care provider conduct |  | 3 | 1 | **3593** |
| 61 | PEQ | Patient Experience Questionnaire |  | 7 | 1 | **1169** |
| 62 | PMH/PSQ-MD | Princess-Margaret Hospital Patient Satisfaction with Doctor Questionnaire |  | 20 | 1 | **6601** |
| 63 | PPE-15 | Picker Patient Experience |  | 20 | 1 | **11583** |
| 64 | PPIQ | Patient-Professional Interaction Questionnaire |  | 16 | 1 | **875** |
| 65 | PSCQ-7 | Patient Satisfaction Consultation Questionnaire |  | 2 | 1 | **1254** |
| 66 | QQPPI | Quality of Of Physician-Patient Interaction |  | 12 | 1 | **4626** |
| 67 |  | Quality of Communication questionnaire ("Physician-family communication" subscale) |  | 4 | 1 | **2051** |
| 68 | RAND PSQ-III | Research and Development Patient Satisfaction Questionnaire |  | 4 | 1 | **311** |
| 69 | SHARED | Patients' experience of SDM during a consultation |  | 10 | 1 | **2379** |
| 70 |  | Stanford Communication with Physicians Scale |  | 2 | 1 | **311** |
| 71 |  | Street Patient Activation Coding System |  | 5 | 1 | **2093** |
| 72 |  | Trust in Physician Scale |  | 1 | 1 | **1747** |
| 73 |  | Patient–family physician interaction | Yes | 7 | 1 | **847** |
| 74 |  | Physician–patient communication in dermatology | Yes | 9 | 1 | **882** |
| 75 |  | Patient participative behavior | Yes | 13 | 1 | **1090** |
| 76 |  | Satisfaction with comprehensive abortion care | Yes | 10 | 1 | **1746** |
| 77 |  | Decision process score | Yes | 4 | 1 | **3534** |
| 78 |  | Dentist-patient communication | Yes | 6 | 1 | **4144** |
| 79 |  | Patient perception of quality of care following a visit to a doctor | Yes | 12 | 1 | **6560** |
| 80 |  | Patient satisfaction | Yes | 2 | 1 | **5495** |
| 81 |  | Satisfaction | Yes | 8 | 1 | **1946** |
| 82 |  | Communication | Yes | 6 | 1 | **5001** |
| 83 |  | Quality of communication | Yes | 5 | 1 | **5715** |
| 84 |  | Bad news transmission | Yes | 9 | 1 | **5988** |
| 85 |  | Nurse-patient communication | Yes | 21 | 1 | **7788** |
| 86 |  | Patient satisfaction | Yes | 8 | 1 | **7905** |
| 87 |  | Patient satisfaction | Yes | 16 | 1 | **8802** |
| 88 |  | Communication | Yes | 4 | 1 | **9061** |
| 89 |  | Satisfaction rating | Yes | 7 | 1 | **9306** |
| 90 |  | Satisfaction with communication | Yes | 5 | 1 | **10095** |
| 91 |  | Patient satisfaction with doctor-patient interaction | Yes | 13 | 1 | **11115** |
| 92 |  | Patient satisfaction | Yes | 11 | 1 | **11184** |
| 93 |  | 360 tool | Yes | 8 | 1 | **5917** |
| 94 |  | Physician inquiry into treatment preferences | Yes | 1 | 1 | **3921** |
| 95 |  | GPs' patient-centeredness | Yes | 2 | 1 | **4783** |
| 96 |  | Relationship-building behaviors | Yes | 11 | 1 | **5115** |
| 97 |  | Patients' satisfaction with clinicians' communication  behaviors (informed by 'Art of Medicine') | Yes | 6 | 1 | **6187** |
| 98 |  | Patient satisfaction | Yes | 4 | 1 | **6357** |
| 99 |  | Patient perceptions of quality of care | Yes | 4 | 1 | **4910** |
| 100 |  | Communication and Relationship factors | Yes | 5 | 1 | **3617** |
| 101 |  | Discrimination | Yes | 6 | 1 | **3013** |
| 102 |  | Informed Decision Making | Yes | 9 | 1 | **5115** |
| 103 |  | Participatory decision-making style of physician | Yes | 3 | 1 | **6028** |
| 104 | PCMH | Patient Centered Medical Home | Yes | 10 | 1 | **3843** |
| 105 | PDDM | Patient-Doctor Discrepancy Model | Yes | 6 | 1 | **2928** |
| 106 |  | Patient prenatal care satisfaction | Yes | 1 | 1 | **3013** |
| 107 |  | Patient satisfaction with last healthcare provider | Yes | 18 | 1 | **4703** |
| 108 |  | Quality of Care | Yes | 19 | 1 | **2709** |
| 109 |  | Experience of and satisfaction with the community pharmacy service | Yes | 8 | 1 | **710** |
| 110 |  | Doctor's communication | Yes | 11 | 1 | **2163** |
| 111 |  | Patient satisfaction | Yes | 5 | 1 | **2815** |
| 112 |  | Patient-clinician interaction | Yes | 14 | 1 | **3956** |
| 113 |  | Communication | Yes | 19 | 1 | **6944** |
| 114 | CSQ | Chiropractic satisfaction questionnaire (adapted version) |  | 9 | 1 | **6212** |
| 115 |  | Communication | Yes | 6 | 1 | **11850** |
| 116 | OPTION-5 | Observing patient involvement in decision making |  | 5 | 1 | **11918** |
| 117 | JSPPPE | Jefferson Scale of Patient Perceptions of Physician Empathy |  | 4 | 1 | **11916** |
| 118 | KOVA | ‘Kommunikationsverhalten’ [communication behavior] (Effective and open communication subscales) |  | 10 | 1 | **12024** |
| 119 | PPCB | Physician-patient communication behavior |  | 17 | 1 | **11846** |
| 120 |  | Patient satisfaction | Yes | 3 | 1 | **12183** |
| 121 |  | Physician–patient interaction | Yes | 3 | 1 | **12203** |
| 122 | MII+ | Method information index |  | 6 | 1 | **12363** |
| 123 |  | Communication Quality | Yes | 5 | 1 | **12363** |
| 124 |  | Patient satisfaction | Yes | 2 | 1 | **12365** |
| 125 | SP-PCRS | Spanish physician-patient communication rating scale | Yes | 14 | 1 | **12383** |
| 126 |  | Satisfaction on shared decision making | Yes | 4 | 1 | **12453** |
| 127 | IPSQ | Internal Patient Satisfaction Questionnaire |  | 1 | 1 | **12575** |
| 128 |  | Communication skills | Yes | 5 | 1 | **13097** |
| 129 |  | Effective communication | Yes | 1 | 1 | **13233** |
| 130 |  | Patient-centeredness (Responsiveness) | Yes | 4 | 1 | **13233** |
| 131 |  | Functional quality of the visit | Yes | 6 | 1 | **3417** |
| 132 | PSQ-III | Patient Satisfaction Questionnaire |  | 2 | 1 | **4077** |
| 133 | PCAS | Primary Care Assessment Survey |  | 5 | 1 | **4836** |
| 134 |  | Satisfaction with clinic quality | Yes | 4 | 1 | **4891** |
| 135 |  | Global relationship-building behaviors | Yes | 4 | 1 | **5115** |
| 136 | PDMstyle | Provider participatory decision-making | Yes | 4 | 1 | **6294** |
| 137 | PCOM | Provider Communication | Yes | 4 | 1 | **6294** |
| 138 | MOS | Medical Outcome Study Visit Rating Scale |  | 2 | 1 | **6548** |
| 139 | P-MISS | Parent Medical Interview Satisfaction Scale |  | 9 | 1 | **10176** |
| 140 |  | Physician Information-Giving Scale |  | 6 | 1 | **12383** |
| 141 |  | Patient Perceived-Decision Making Scale |  | 3 | 1 | **12383** |
| 142 |  | Patient Choice in Medical Care/Treatment Decisions Scale |  | 4 | 1 | **12383** |

**APPENDIX F. OVERVIEW TABLES OF ALL ITEMS, SORTED BY DIMENSION AND ACTION TERMS.**

**F.1 DIMENSION 1: Patients’ unique situation**

|  |  | **Item** | **Responses** | **Respondent** | **Instrument ID#** |
| --- | --- | --- | --- | --- | --- |
| **Facilitating patient involvement** | |  |  |  |  |
|  | **Involving** | Discussed and agreed together what the problem was | **7-point Likert scale**  Very strongly disagree  Very strongly agree | Patient | 19 |
|  |  | Did you and your doctor decide together which problems and concerns you would talk about today? | **5-point Likert scale**  Lowest satisfaction  Highest satisfaction | Patient | 52 |
|  | **Encouraging** | Practitioner encourages patient to talk about current behavior or status quo | **5-point Likert scale**  0 = Not at all  1 = Minimally  2 = To some extent  3 = A good deal  4 = A great extent | Observer | 31 |
|  |  | Encouraged me to express my thoughts concerning my health problems | **5-point Likert scale**  Strongly disagree  Strongly agree | Patient and Clinician | 47 |
|  |  | My HCP encourages me to talk about personal concerns related to my symptoms | **5-point Likert scale**  1 = All the time  5 = Never | Patient | 49 |
|  |  | My doctor encouraged me to talk about my concerns related to my condition | **5-point Likert scale**  Very strong disagreement with the statement  Very strong agreement with the statement | Patient | 11 |
|  |  | The health care provider didn’t encourage me to talk about all my problems and concerns | **4-point Likert scale**  1 = Strongly disagree  4 = Strongly agree | Patient | 76 |
|  |  | Encouraged you to express your thoughts concerning health problems | **5-point Likert scale**  1 = Strongly disagree  2 = Disagree  3 = Neither agree nor disagree  4 = Agree  5 = Strongly agree | Patient | 119 |
|  | **Allowing** | Letting you tell your story; listening carefully; asking thoughtful questions; not interrupting you while you’re talking | **5-point Likert scale**  Poor  Excellent | patient | 18 |
|  |  | Letting you tell your "story" (giving you time to fully describe your illness in your own words; not interrupting or diverting you) | **5-point Likert scale**  Poor  Excellent | Patient | 4 |
|  |  | Opportunity for patient to express concerns and fears | **5-point Likert scale**  1 = Poor  5 = Excellent | Patient | 5 |
|  |  | The opportunity the doctor gave me to express my concerns or fears was | **5-point Likert scale**  Poor  Excellent | Patient | 24 |
|  |  | The doctor did not allow me to say everything I had wanted about my problems | **7-point Likert scale**  1 = Very strongly disagree  2 = Strongly disagree  3 = Disagree  4 = Unsure  5 = Agree  6 = Strongly agree  7 = Very strongly agree | Patient | 10 |
|  |  | How much did your doctor wait to talk about all your problems before starting to talk about one in particular? | **5-point Likert scale**  Lowest satisfaction  Highest satisfaction | Patient | 52 |
|  |  | The doctor gave me enough chance to talk about all my problems | **5-point Likert scale**  I do not agree  I fully agree | Patient | 66 |
|  |  | Providing space for feelings and emotions | **4-point Likert scale**  1 = Not at all  2 = Not really  3 = On the whole, yes  4 = Yes | Observer | 16 |
|  |  | Were you able to speak about your problems and receive the appropriate attention? | **6-point Likert scale**  1 = Poor  6 = Excellent | Patient | 99 |
|  |  | Opportunities for me to express thoughts/concerns | **7-point Likert scale**  Strongly disagree  Strongly agree | Patient | 107 |
| **Adjusting to individual patient** | |  |  |  |  |
|  | **Tailoring (general)** | Tailoring the information to the patients’ personal circumstances | **4-point Likert scale**  1 = Not at all  2 = Not really  3 = On the whole, yes  4 = Yes | Observer | 16 |
|  |  | Tailoring information to the patient’s situation | **4-point Likert scale**  1 = Not at all  2 = Not really  3 = On the whole, yes  4 = Yes | Observer | 16 |
|  | **Tailoring care** | Consideration of patient's personal situation in treatment or advice | **5-point Likert scale**  1 = Poor  5 = Excellent | Patient | 5 |
|  |  | This doctor's consideration of my personal situation in deciding a treatment or advising me was | **5-point Likert scale**  Poor  Excellent | Patient | 24 |
|  |  | Considering the patients’ current capabilities | **4-point Likert scale**  1 = Not at all  2 = Not really  3 = On the whole, yes  4 = Yes | Observer | 16 |
|  |  | Taking all your medical history into account when considering your current problem or treatment | **5-point Likert scale**  Poor  Fair  Good  Very good  Excellent | Patient | 96 |
| **Providing information** | |  |  |  |  |
|  | **Informing** | Explaining what you need to know about your problems, how and why they occurred, and what to expect next | **5-point Likert scale**  Poor  Excellent | Patient | 18 |
|  |  | Did doctor give instructions on taking care of health problems/concerns? | No  Somewhat  Yes  Yes definitely | Patient | 3 |
|  |  | Explained clearly what the problem was | **7-point Likert scale**  Very strongly disagree  Very strongly agree | Patient | 19 |
|  |  | Was definite about what the problem was | **7-point Likert scale**  Very strongly disagree  Very strongly agree | Patient | 19 |
|  |  | How much effort was made to help you understand your health issues? | 0 = No effort was made  9 = Every effort was made | Patient | 33 |
|  |  | The doctor told me how long I could expect to have this problem | No  Yes  N/A | Patient | 36 |
|  |  | I received enough information about my illness | **5-point Likert scale**  Totally disagree  Totally agree | Patient | 44 |
|  |  | Did the doctors give you enough information about your health problems? | **5-point Likert scale** | Patient | 7 |
|  |  | The diagnosis of his or her medical problem | **7-point Likert scale**  Strongly disagree  Strongly agree | Clinician | 46 |
|  |  | The causes of his or her medical problem | **7-point Likert scale**  Strongly disagree  Strongly agree | Clinician | 46 |
|  |  | The long-term consequences of his or her medical problem | **7-point Likert scale**  Strongly disagree  Strongly agree | Clinician | 46 |
|  |  | The doctor told me just what my trouble is | **7-point Likert scale**  1 = Very strongly disagree  2 = Strongly disagree  3 = Disagree  4 = Unsure  5 = Agree  6 = Strongly agree  7 = Very strongly agree | Patient | 10 |
|  |  | The doctor was good at explaining the reason for my ill health | **5-point Likert scale**  Strongly disagree  Strongly agree | Patient | 48 |
|  |  | I talk about pain symptoms regardless of my HCP’s reactions when I do so | 1 = All the time  5 = Never | Patient | 49 |
|  |  | The clinician draws attention to an identified problem as one that requires a decision making process | 0 = The behaviour is not observed  4 = The behaviour is exhibited to a very high standard | Observer | 8 |
|  |  | I talked in great detail about my symptoms and problems with self-care | **5-point Likert scale**  Very strong disagreement with the statement  Very strong agreement with the statement | Patient | 11 |
|  |  | Did you discuss any personal problems that may be related to your illness? | No  Somewhat  Yes  Yes definitely | Patient | 70 |
|  |  | For patients, occurrence of: Expressions of concern | Coding scheme of utterances | Observer | 71 |
|  |  | Clarification of the health problem | **5-point Likert scale**  Negative  Positive | Patient | 79 |
|  |  | Explanation of your problem | **4-point Likert scale**  Very dissatisfied  Very satisfied  (and option “no opinion or not concerned”) | Patient | 81 |
|  |  | Were the topics below discussed with the radiotherapist?: Physical problems | 0 = Not applicable  0 = No  1 = More or less  2 = Yes | Patient | 88 |
|  |  | Were the topics below discussed with the radiotherapist?: Emotional problems | 0 = Not applicable  0 = No  1 = More or less  2 = Yes | Patient | 88 |
|  |  | Were the topics below discussed with the radiotherapist?: Sexual problems | 0 = Not applicable  0 = No  1 = More or less  2 = Yes | Patient | 88 |
|  |  | Explaining what you need to know about your problems, how and why they occurred and what to expect next | **5-point Likert scale**  Poor  Fair  Good  Very good  Excellent | Patient | 96 |
|  |  | Dermatologist's explanations on health problem | **5-point Likert scale**  Poor  Fair  Good  Very good  Excellent | Patient | 98 |
|  |  | Discuss nature of decision: What are the essential clinical issues we are addressing? | Absence  Presence | Observer | 102 |
|  |  | The physician explains the illness to me | **4-point Likert scale**  Not at all  Very much | Patient and Clinician | 105 |
|  |  | My doctor clearly explained me what my medical condition was | **5-point Likert scale**  Strongly disagree  Strongly agree | Patient | 110 |
|  |  | Your physician explained to you exactly what your diagnosis means | **6-point Likert scale**  1 = Strongly disagree  6 = Strongly agree | Patient and Clinician | 118 |
|  |  | The physician was good at explaining the reason for my complaints | **5-point Likert scale**  Strongly disagree  Disagree  Uncertain  Agree  Strongly agree | Patient | 121 |
|  |  | Did the FWA explain the purpose of the visit? | No  Yes | Patient | 123 |
|  |  | How satisfied are you with how well the healthcare worker explained your health condition? | **5-point Likert scale**  Very dissatisfied  Somewhat dissatisfied  Neither satisfied nor dissatisfied  Somewhat satisfied  Very satisfied | Patient | 124 |
|  |  | Physician adequately communicates the disease diagnosis | Missing | Patient | 131 |
|  |  | Doctor's explanations of your health problems or treatments | Missing | Patient | 133 |
|  |  | Explanation of diagnosis and treatment | **5-point Likert scale**  Very dissatisfied/unwilling  Very satisfied/willing | Patient | 134 |
|  | **Being transparent** | Your physician always told you everything about your illness, even if it is unpleasant | **6-point Likert scale**  1 = Strongly disagree  6 = Strongly agree | Patient and Clinician | 118 |
|  | **Addressing** | The doctor/nurse looked into all the problems I mentioned | **5-point Likert scale**  Strongly disagree  Strongly agree | Patient | 37 |
|  |  | Responded to my questions and concerns | **5-point Likert scale**  Strongly disagree  Strongly agree | Patient and Clinician | 47 |
|  |  | The doctor has relieved my worries about my illness | **7-point Likert scale**  1 = Very strongly disagree  2 = Strongly disagree  3 = Disagree  4 = Unsure  5 = Agree  6 = Strongly agree  7 = Very strongly agree | Patient | 10 |
|  |  | The doctor did not relieve my worries about my illness | **5-point Likert scale**  Strongly disagree  Strongly agree | Patient | 48 |
|  |  | I did not feel that anything important was missed during my visit with my doctor | **5-point Likert scale**  1 = Strong disagreement  3 = Agreement  5 = Strong agreement | Patient | 55 |
|  |  | I felt that the doctor and the nurse answered all my questions and concerns | **5-point Likert scale**  1 = Strong disagreement  3 = Agreement  5 = Strong agreement | Patient | 55 |
|  |  | Medical issues received considerable attention during consultation | Received considerable attention  Received hardly/no attention | Observer | 82 |
|  |  | Did the doctor clear your doubts? | No  Yes | Patient | 83 |
|  |  | Addressing physical symptoms | **5-point Likert scale**  Extremely bad  Extremely good | Patient | 86 |
|  |  | I have health problems which should have been discussed today, but were not | Never  Occasionally  Sometimes  Often  Always | Patient | 92 |
|  |  | Cleared the doubts I had about my eye condition | **5-point Likert scale**  Strongly disagree  Strongly agree | Patient | 112 |
|  |  | Helped in reducing your worries about this eye condition | **5-point Likert scale**  Very dissatisfied  Very satisfied | Patient | 112 |
|  |  | Responded to your questions and concerns | **5-point Likert scale**  1 = Strongly disagree  2 = Disagree  3 = Neither agree nor disagree  4 = Agree  5 = Strongly agree | Patient | 119 |
|  |  | Responsiveness (how the surgeon reacted to patients’ concerns, explored patients’ ideas and acknowledged patients’ expectations) | 1-5  (Higher = Better) |  | 135 |
| **Gathering/having needed information** | |  |  |  |  |
|  | **Exploring** | Asking about your symptoms | **5-point Likert scale**  Very poor  Very good | Patient | 41 |
|  |  | Discussed my reason(s) for coming today | **5-point Likert scale**  Strongly disagree  Strongly agree | Patient and Clinician | 47 |
|  |  | My HCP asks me what I believe is causing my medical symptoms | 1 = All the time  5 = Never | Patient | 49 |
|  |  | Asking about your symptoms | Doesn’t apply  Very poor  Poor  Neither good nor poor  Good  Very good | Patient | 50 |
|  |  | Asked questions, either directly or on a survey, about my health habits | **5-point Likert scale**  Almost never  Generally not  Sometimes  Most of the time  Almost always | Patient | 14 |
|  |  | Asked how my visits with other doctors were going | **5-point Likert scale**  Almost never  Generally not  Sometimes  Most of the time  Almost always | Patient | 14 |
|  |  | How much did your doctor ask to hear your ideas and explanations concerning your health problems? | **5-point Likert scale**  Lowest satisfaction  Highest satisfaction | Patient | 52 |
|  |  | How much did your doctor try to find out about all your problems and concerns? | **5-point Likert scale**  Lowest satisfaction  Highest satisfaction | Patient | 52 |
|  |  | How much did your doctor inquire about your emotions or feelings? | **5-point Likert scale**  Lowest satisfaction  Highest satisfaction | Patient | 52 |
|  |  | How much did your provider try to find out about all your problems and concerns? | **5-point Likert scale**  1 = Very little agreement  5 = Very much in agreement | Patient | 56 |
|  |  | The doctor did not ask me about any other health problems | **6-point Likert scale**  Strongly disagree  Strongly agree | Patient or Observer | 57 |
|  |  | The doctor asked me for my ideas about my health problem | **6-point Likert scale**  Strongly disagree  Strongly agree | Patient or Observer | 57 |
|  |  | My doctor asked me what might be causing some of my problems | **5-point Likert scale**  Very strong disagreement with the statement  Very strong agreement with the statement | Patient | 11 |
|  |  | Doctor didn't discuss anxieties or fears | 1 = Yes completely  2 = Yes to some extent  3 = No | Patient and Caregiver | 63 |
|  |  | Nurses didn't discuss anxieties or fears | 1 = Yes completely  2 = Yes to some extent  3 = No | Patient and Caregiver | 63 |
|  |  | Exploring the patients’ worries and anxieties | **4-point Likert scale**  Not at all  Not really  On the whole, yes  Yes | Observer | 16 |
|  |  | The physician asked questions about my health problem | Disagree  Agree | Patient | 73 |
|  |  | I got all the detail I needed regarding the patient's history | **5-point Likert scale**  Strongly disagree  Strongly agree | Clinician | 29 |
|  |  | I didn't get all the detail I wanted on the patient's problem and symptoms | **5-point Likert scale**  Strongly disagree  Strongly agree | Clinician | 29 |
|  |  | I didn't get enough detail from this patient regarding his/her psychosocial condition | **5-point Likert scale**  Strongly disagree  Strongly agree | Clinician | 29 |
|  |  | History of the health problem taken by the MD | **5-point Likert scale**  Negative  Positive | Patient | 79 |
|  |  | Psychological themes (coping, stress, concerns, anxiety, and other emotions) received considerable attention during consultation | Received considerable attention  Received hardly/no attention | Observer | 82 |
|  |  | Social environment of patient (partner, family, work, home situation) received considerable attention during consultation | Received considerable attention  Received hardly/no attention | Observer | 82 |
|  |  | Did the doctor ask about your previous knowledge of the disease? | No  Yes | Patient | 83 |
|  |  | Asking about your symptoms | **5-point Likert scale**  Extremely bad  Extremely good | Patient | 86 |
|  |  | Talked with you about your prescriptions | Not at all  A little  Some  A lot | Patient | 104 |
|  |  | Talked about personal or family problem/alcohol or drug use | No  Yes | Patient | 104 |
|  |  | Talked about worry and stress in your life | No  Yes | Patient | 104 |
|  |  | Talked about feeling sad or depressed | No  Yes | Patient | 104 |
|  |  | The CP asked about any over the counter medicines I may be taking | Strongly disagree  Strongly agree | Patient | 109 |
|  |  | Your physician asked at the beginning of your treatment to explain all your symptoms | **6-point Likert scale**  1 = Strongly disagree  6 = Strongly agree | Patient and Clinician | 118 |
|  |  | Your physician asked about all your symptoms | **6-point Likert scale**  1 = Strongly disagree  6 = Strongly agree | Patient and Clinician | 118 |
|  |  | Your physician asked whether you experience pain during therapy/treatment | **6-point Likert scale**  1 = Strongly disagree  6 = Strongly agree | Patient and Clinician | 118 |
|  |  | Discussed your reason(s) for coming | **5-point Likert scale**  1 = Strongly disagree  2 = Disagree  3 = Neither agree nor disagree  4 = Agree  5 = Strongly agree | Patient | 119 |
|  |  | Thoroughness of doctor's questions about your symptoms and how you are feeling | Missing | Patient | 133 |
|  | **Understanding** | Did doctor seem to know important information about your medical history? | No  Yes, somewhat  Yes, definitely | Patient | 3 |
|  |  | Understood my main health concerns | Poor  Fair  Good  Very good  Excellent | Proxy/Caregiver | 6 |
|  |  | There are some things this doctor does not know about me | **5-point Likert scale**  Strongly disagree  Strongly agree | Patient | 35 |
|  |  | This doctor knows all about me | **5-point Likert scale**  Strongly disagree  Strongly agree | Patient | 35 |
|  |  | The care professional understood my problems and complaints | **4-point Likert scale**  No  Yes | Patient | 20 |
|  |  | This doctor does not know my medical history very well | **5-point Likert scale**  Strongly disagree  Strongly agree | Patient | 21 |
|  |  | My provider(s) is up to date on my medical history | **4-point Likert scale**  Strongly disagree  Strongly agree | Patient | 42 |
|  |  | The doctor did not really understand my main reason for coming | **7-point Likert scale**  1 = Very strongly disagree  2 = Strongly disagree  3 = Disagree  4 = Unsure  5 = Agree  6 = Strongly agree  7 = Very strongly agree | Patient | 10 |
|  |  | This doctor/nurse/health visitor knows all about me | **5-point Likert scale**  Strongly disagree  Strongly agree | Patient | 51 |
|  |  | There are some things this doctor/nurse/ health visitor does not know about me | **5-point Likert scale**  Strongly disagree  Strongly agree | Patient | 51 |
|  |  | How much did your doctor understand what you were going through emotionally? | **5-point Likert scale**  Lowest satisfaction  Highest satisfaction | Patient | 52 |
|  |  | My doctor had a complete understanding of the things that are wrong with me | **5-point Likert scale**  Lowest satisfaction  Highest satisfaction | Patient | 52 |
|  |  | My doctor showed a good understanding of my past health history | **5-point Likert scale**  Lowest satisfaction  Highest satisfaction | Patient | 52 |
|  |  | My doctor missed important information that I gave her/him | **5-point Likert scale**  Lowest satisfaction  Highest satisfaction | Patient | 52 |
|  |  | I felt the specialist was able to understand my situation and provide satisfactory care | **5-point Likert scale**  1 = Strong disagreement  3 = Agreement  5 = Strong agreement | Patient | 55 |
|  |  | How much did your provider understand what you were going through emotionally? | **5-point Likert scale**  1 = Very little agreement  5 = Very much in agreement | Patient | 56 |
|  |  | My provider had a complete understanding of the things that are wrong with me | **5-point Likert scale**  1 = Very little agreement  5 = Very much in agreement | Patient | 56 |
|  |  | The doctor really understood what I was saying about my health | **6-point Likert scale**  Strongly disagree  Strongly agree | Patient or Observer | 57 |
|  |  | Did nurses show understanding for your situation? | No, none at all  Some  Much  Yes, absolutely | Patient | 58 |
|  |  | My doctor and I agree on the nature of my medical symptoms | **5-point Likert scale**  Not at all appropriate  Totally appropriate | Patient | 26 |
|  |  | Quality of time: I felt the doctor diagnosed my condition without enough information | **4-point Likert scale**  Does not apply  Strongly disagree  Disagree  Agree  Strongly agree | Patient | 62 |
|  |  | Did you get the impression that the doctor had an extensive knowledge of your health problem? | **5-point Likert scale**  Not at all  A lot | Patient | 65 |
|  |  | Understanding the patients’ personal circumstances | **4-point Likert scale**  Not at all  Yes | Observer | 16 |
|  |  | During these consultations (medical issues) GP displayed prior knowledge | Missing | Observer | 82 |
|  |  | During these consultations (Psych themes) GP displayed prior knowledge | Missing | Observer | 82 |
|  |  | During these consultations (social environment discussed) GP displayed prior knowledge | Missing | Observer | 82 |
|  |  | The doctor understood my problems | **4-point Likert scale**  Not at all  Very much so | Patient | 91 |
|  |  | The doctor understood how I was feeling | **4-point Likert scale**  Not at all  Very much so | Patient | 91 |
|  |  | My doctor has a reasonable understanding of my life circumstances | Never  Occasionally  Sometimes  Often  Always | Patient | 92 |
|  |  | My doctor has a good understanding of my past health history | Never  Occasionally  Sometimes  Often  Always | Patient | 92 |
|  |  | How well did the doctor understand your problem? | **9-point Likert scale**  Did not understand at all  Understood very well | Patient | 97 |
|  |  | Did he/she show that he/she understood your problem? | **6-point Likert scale**  1 = Poor  6 = Excellent | Patient | 99 |
|  |  | My HIV provider really knows me as a person | Don’t know  No  Yes | Patient | 100 |
|  |  | Provider seemed informed and up-to-date about care you got from specialists | Not at all  A little  Some  A lot | Patient | 104 |
|  |  | She/he had all the information needed to assist me | **10-point Likert scale**  Completely disagree  Completely agree | Patient | 108 |
|  |  | The doctor understood how I was feeling | **5-point Likert scale**  I disagree completely  I agree completely | Patient | 113 |
|  |  | Understanding of your health problem | Very poor  Best | Patient | 114 |
|  |  | Understands my emotions, feelings and concerns | **7-point Likert scale**  1 = Strongly disagree  7 = Strongly agree | Patient | 117 |
|  |  | Did the healthcare worker understand your problem? | **5-point Likert scale**  Very dissatisfied  Somewhat dissatisfied  Neither satisfied nor dissatisfied  Somewhat satisfied  Very satisfied | Patient | 124 |
|  |  | The doctor failed to understand my main reason for coming | **5-point Likert scale** | Caregiver | 139 |
|  | **Checking** | Fully understanding your concerns (communicating that he/she had accurately understood your concerns; not overlooking or dismissing anything) | **5-point Likert scale**  Poor  Excellent | Patient | 4 |
|  |  | Did the doctors make sure you understand your health problems? | **5-point Likert scale** | Patient | 7 |
|  |  | Did the doctors go over all the medicines you are taking? | **5-point Likert scale** | Patient | 7 |
|  |  | This doctor/nurse/health visitor was very careful to check everything when examining me/carrying out my care/ discussing my family's health | **5-point Likert scale**  Strongly disagree  Strongly agree | Patient | 51 |
|  |  | The nurse says his/her impressions of the patient's condition to him/her (Sadness, distress, anxiety, joy) | 0 = Not done  1 = Done incorrectly  2 = Done correctly | Observer | 85 |
| **Making human connection** | |  |  |  |  |
|  | **Caring** | Dermatologist's concern for patient's health | **5-point Likert scale**  Poor  Fair  Good  Very good  Excellent | Patient | 98 |
|  |  | Concern the care provider showed for your questions or worries | **5-point Likert scale** | Patient | 17 |
|  |  | The doctor seemed to think about my child’s problem carefully | **5-point Likert scale** | Caregiver | 139 |
|  | **Connecting** | Making it easy for you to tell him or her about your problem? | **5-point Likert scale**  Poor  Excellent | Patient | 23 |
|  |  | I felt comfortable discussing my problems with the physician today | **5-point Likert scale**  Lowest satisfaction  Highest satisfaction | Patient | 52 |
|  |  | I could tell this doctor about very personal problems | **4-point Likert scale**  Not at all  Very much so | Patient | 91 |
|  | **Sympathizing** | Practitioner acknowledges challenges about behavior change that the patient faces | **5-point Likert scale**  0 = Not at all  1 = Minimally  2 = To some extent  3 = A good deal  4 = A great extent | Observer | 31 |
|  |  | Sufficient concern was shown about my state of health | **5-point Likert scale**  Totally disagree  Totally agree | Patient | 44 |
|  |  | How often did your physician have empathy for your emotions and your current situation? | Never  Sometimes  Usually  Always | Patient | 58 |
|  |  | To believe my symptoms | **7-point Likert scale**  Strongly disagree  Strongly agree | Patient | 107 |
|  |  | The doctor empathized with my situation; showed care and concern | **5-point Likert scale**  Strongly disagree  Strongly agree | Patient | 110 |
|  | **Respecting** | Taking your problems seriously | **5-point Likert scale**  Very poor  Very good | Patient | 41 |
|  |  | Staff were sensitive to my cultural background | **5-point Likert scale**  Strongly disagree  Strongly agree | Patient | 45 |
|  |  | Did the doctors take your concerns seriously? | **5-point Likert scale** | Patient | 7 |
|  |  | The doctor seemed to take my problems seriously | **7-point Likert scale**  1 = Very strongly disagree  2 = Strongly disagree  3 = Disagree  4 = Unsure  5 = Agree  6 = Strongly agree  7 = Very strongly agree | Patient | 10 |
|  |  | Taking your problems seriously | Doesn’t apply  Very poor  Poor  Neither good nor poor  Good  Very good | Patient | 50 |
|  |  | Do you think that your doctor took your health problem seriously? | **5-point Likert scale**  Not at all  A lot | Patient | 65 |
|  |  | The nurse accepts the patient away from the cultural characteristics (Race, ethnicity, religion) | 0 = Not done  1 = Done incorrectly  2 = Done correctly | Observer | 85 |
|  |  | The nurse accepts the patient away from social features (Level of education, marital status, occupation) | 0 = Not done  1 = Done incorrectly  2 = Done correctly | Observer | 85 |
|  |  | The nurse accepts the patient away from economic characteristics (Income, economic level) | 0 = Not done  1 = Done incorrectly  2 = Done correctly | Observer | 85 |
|  |  | Taking your problems seriously | **5-point Likert scale**  Extremely bad  Extremely good | Patient | 86 |
|  |  | The doctor took my problems seriously | **4-point Likert scale**  Not at all  Very much so | Patient | 91 |
|  |  | My concerns were taken seriously | Strongly disagree  Strongly agree | Patient | 109 |
|  | **Being courteous** | My emotional needs (worries, fears, anxieties) were recognized and taken seriously by the program staff | **5-point Likert scale**  Definitely not true  Completely true | Patient | 32 |
| **Giving attention** | |  |  |  |  |
|  | **Showing interest** | Was interested in my worries about the problem | **7-point Likert scale**  Very strongly disagree  Very strongly agree | Patient | 19 |
|  |  | Was interested when I talked about my symptoms | **7-point Likert scale**  Very strongly disagree  Very strongly agree | Patient | 19 |
|  |  | Was interested in what I thought the problem was | **7-point Likert scale**  Very strongly disagree  Very strongly agree | Patient | 19 |
|  |  | Interest in your personal situation? | **5-point Likert scale**  Poor  Excellent | Patient | 23 |
|  |  | How often did your physician show an interest in your personal situation? | Never  Sometimes  Usually  Always | Patient | 58 |
|  |  | He/she was interested in what I feel about my current health status | **5-point Likert scale**  1 = Not at all  5 = Very much | Patient | 64 |
|  |  | He/she was interested in what I know about my disease/prognosis | **5-point Likert scale**  1 = Not at all  5 = Very much | Patient | 64 |
|  |  | The physician seemed to be genuinely interested in my problems | **5-point Likert scale**  I do not agree  I fully agree | Patient | 66 |
|  |  | Interest of the physician for your problem | **4-point Likert scale**  Very dissatisfied  Very satisfied  (and option “no opinion or not concerned”) | Patient | 81 |
|  |  | Physician is interested in the patient’s financial situation | Missing | Patient | 131 |
|  | **Listening** | Their willingness to listen to all of your concerns? | **5-point Likert scale**  Poor  Excellent | Patient | 38 |
|  |  | Asking you questions about the reasons for your visit and listening carefully to your responses | Can’t say  Poor  Fair  Good  Very good  Excellent | Patient | 53 |
|  |  | How well did the doctor listen to your concerns and questions? | **9-point Likert scale**  Did not listen at all  Listened very well | Patient | 97 |
|  |  | Listening (how the surgeon facilitated and responded to patients’ expressions of concerns and questions) | 1-5  (Higher = Better) | Observer | 135 |
|  | **Noticing** | My pain was noticed and taken seriously | **5-point Likert scale**  Totally disagree  Totally agree | Patient | 44 |
|  |  | Being attentive to how the patient is doing | **4-point Likert scale**  Not at all  Yes | Observer | 16 |
|  | **Ignoring** | The medical problems I had in the past were ignored during my visit | **5-point Likert scale**  Lowest satisfaction  Highest satisfaction | Patient | 52 |
|  |  | Sometimes I feel the doctor ignores my concern | **5-point Likert scale**  Strongly disagree  Strongly agree | Patient | 110 |
| **Self-Efficacy building** | |  |  |  |  |
|  | **Reassuring** | She/he reassured me concerning my worries | **10-point Likert scale**  Completely disagree  Completely agree | Patient | 108 |
|  | **Supporting** | The AP helped me understand my condition | **5-point Likert scale**  Strongly disagree  Strongly agree | Patient | 59 |

**F2 Dimension 2: Patient priorities**

|  |  | | **Item** | **Responses** | **Respondent** | **Instrument ID#** |
| --- | --- | --- | --- | --- | --- | --- |
| **Facilitating patient involvement** | | |  |  |  |  |
|  | **Involving** | | Involved me in decisions as much as I wanted | Poor  Fair  Good  Very good  Excellent | Proxy/Caregiver | 6 |
|  |  | | Involved me in decisions as much as I wanted | **5-point Likert scale**  Strongly disagree  Strongly agree | Patient and Clinician | 47 |
|  |  | | Let you choose when to receive information and the type of information you want | **7-point Likert scale**  Not at all perceived  Perceived to a very great extent | Proxy/Caregiver | 25 |
|  |  | | Did you and your doctor decide together which of your concerns were most important to you? | **5-point Likert scale**  Lowest satisfaction  Highest satisfaction | Patient | 52 |
|  |  | | How often was your physician open to your opinion and ideas about treatment? | Never  Sometimes  Usually  Always | Patient | 58 |
|  |  | | Did you want to be more involved in decisions made about your care and treatment? | 1 = Yes completely  2 = Yes to some extent  3 = No | Patient and Caregiver | 63 |
|  |  | | Involving you in the decisions about the treatments that you want if you get too sick to speak for yourself | **11-point Likert scale**  The very worst  The very best | Patient | 15 |
|  |  | | Involved you in decisions about your health as much as you wanted | **5-point Likert scale**  1 = Strongly disagree  2 = Disagree  3 = Neither agree nor disagree  4 = Agree  5 = Strongly agree | Patient | 119 |
|  | **Co-creating** | | I set clear goals for my care together with the staff | **5-point Likert scale**  Totally disagree  Totally agree | Patient | 44 |
|  |  | | Work with you to set specific goals for your health | No  Yes | Patient | 104 |
|  | **Encouraging** | | My doctor discourages me from expressing my personal opinion about my medical condition | **6-point Likert scale**  None  All of the time | Patient | 40 |
|  | **Allowing** | | The doctor gave me the chance to express my opinions about the different treatments available | **5-point Likert scale**  Strongly disagree  Strongly agree | Patient | 2 |
|  |  | | The doctor gave me the chance to ask for as much information as I needed about the different treatment choices | **5-point Likert scale**  Strongly disagree  Strongly agree | Patient | 2 |
|  |  | | The doctor gave me a chance to decide which treatment I thought was best for me | **5-point Likert scale**  Strongly disagree  Strongly agree | Patient | 2 |
|  |  | | My HCP focuses on just one or two topics during the medical appointment so it’s hard for me to bring up other issues or concerns that I may have | 1 = All the time  5 = Never | Patient | 49 |
|  |  | | During my medical visits, I am always allowed to say anything that I think is important | **5-point Likert scale**  Strongly disagree  Strongly agree | Patient | 68 |
|  |  | | According to the needs of the patient, the nurse gives the opportunity to the patient to talk | 0 = Not done  1 = Done incorrectly  2 = Done correctly | Observer | 85 |
| **Adjusting to individual patient** | |  |  |  |  |  |
|  | **Tailoring care** | | The program staff took my individual needs into consideration when planning my care | **5-point Likert scale**  Definitely not true  Completely true | Patient | 32 |
|  |  | | How much effort was made to include what matters most to you in choosing what to do next? | 0 = No effort was made  9 = Every effort was made | Patient | 33 |
|  |  | | The care professional considered my preferences | **4-point Likert scale**  No  Yes | Patient | 20 |
|  |  | | Did the doctors make decision without taking your preferences and opinions into account? | **5-point Likert scale** | Patient | 7 |
|  |  | | Sure that my doctor or nurse thought about my values and my traditions when they recommended treatments to me | **5-point Likert scale**  Almost never  Generally not  Sometimes  Most of the time  Almost always | Patient | 14 |
|  |  | | Empathy: The doctor considered my individual needs when treating my condition | **4-point Likert scale**  Does not apply  Strongly disagree  Disagree  Agree  Strongly agree | Patient | 62 |
|  |  | | I felt the decision made was the best one for me | **5-point Likert scale**  Strongly disagree  Strongly agree | Patient | 69 |
|  |  | | The clinician makes an effort to integrate the patient’s elicited preferences as decisions are made If the patient indicates how best to integrate their preferences as decisions are made, the clinician makes an effort to do so | 0 = No effort  1 = Minimal effort  2 = Moderate effort  3 = Skilled effort  4 = Exemplary effort | Observer | 116 |
|  |  | | Take your preferences into account when making treatment decisions | 1-100  (Higher = Better) | Patient | 136 |
|  |  | | Physician took preferences into account | **5-point Likert scale** | Observer | 142 |
| **Providing information** | |  |  |  |  |  |
|  | **Informing** | | Did you get as much information about your child’s condition and treatment as you wanted? | No  Somewhat  Yes  Yes definitely | Patient or Caregiver | 30 |
|  |  | | Gave me as much information as I wanted | Poor  Fair  Good  Very good  Excellent | Proxy/Caregiver | 6 |
|  |  | | Telling you what you wanted to know about your symptoms and/or illness? | **5-point Likert scale**  Poor  Excellent | Patient | 23 |
|  |  | | My doctor gives me all the information that I need to make decisions that are right for me | **6-point Likert scale**  None  All of the time | Patient | 40 |
|  |  | | Gave me as much information as I wanted | **5-point Likert scale**  Strongly disagree  Strongly agree | Patient and Clinician | 47 |
|  |  | | The doctor told me all I wanted to know about my illness | **7-point Likert scale**  1 = Very strongly disagree  2 = Strongly disagree  3 = Disagree  4 = Unsure  5 = Agree  6 = Strongly agree  7 = Very strongly agree | Patient | 10 |
|  |  | | The advanced practitioner gave me the information I needed | **5-point Likert scale**  Strongly disagree  Strongly agree | Patient | 59 |
|  |  | | I told the doctor what my preferences were for treating the disease | **5-point Likert scale**  Very strong disagreement with the statement  Very strong agreement with the statement | Patient | 11 |
|  |  | | I made specific suggestions for treatment | **5-point Likert scale**  Very strong disagreement with the statement  Very strong agreement with the statement | Patient | 11 |
|  |  | | I talked about what was important to me about this decision | **5-point Likert scale**  Strongly disagree  Strongly agree | Patient | 69 |
|  |  | | I talked about why one option suited me better than another | **5-point Likert scale**  Strongly disagree  Strongly agree | Patient | 69 |
|  |  | | I express a desire to my HCP that I would like him or her to help me deal with health care needs | 1 = Never  5 = Always  (Or “Not applicable”) | Patient | 75 |
|  |  | | My doctor provided me as much information as I wanted | **5-point Likert scale**  Strongly disagree  Strongly agree | Patient | 110 |
|  |  | | Gave you as much information as you wanted | **5-point Likert scale**  1 = Strongly disagree  2 = Disagree  3 = Neither agree nor disagree  4 = Agree  5 = Strongly agree | Patient | 119 |
|  | **Addressing** | | How often did the doctor address your needs as a whole person? | 0 = Never/Not at all  10 = Always/Completely | Proxy/Caregiver | 43 |
|  |  | | My needs were addressed | **5-point Likert scale**  Strongly disagree  Strongly agree | Patient | 59 |
| **Gathering/having needed information** | |  |  |  |  |  |
|  | **Exploring** | | Practitioner asks questions to elicit how patient thinks and feels about the topic | **5-point Likert scale**  0 = Not at all  1 = Minimally  2 = To some extent  3 = A good deal  4 = A great extent | Observer | 31 |
|  |  | | The clinician assesses the patient’s preferred approach to receiving information to assist decision making (eg, discussion, reading printed material, assessing graphical data, using videotapes or other media) | 0 = The behaviour is not observed  4 = The behaviour is exhibited to a very high standard | Observer | 8 |
|  |  | | The clinician explores the patient’s expectations (or ideas) about how the problem(s) are to be managed | 0 = The behaviour is not observed  4 = The behaviour is exhibited to a very high standard | Observer | 8 |
|  |  | | The clinician elicits the patient’s preferred level of involvement in decision-making | 0 = The behaviour is not observed  4 = The behaviour is exhibited to a very high standard | Observer | 8 |
|  |  | | Asked to talk about my goals in caring for my illness | **5-point Likert scale**  Almost never  Generally not  Sometimes  Most of the time  Almost always | Patient | 14 |
|  |  | | How much did your doctor ask you exactly what you expected in this visit? | **5-point Likert scale**  Lowest satisfaction  Highest satisfaction | Patient | 52 |
|  |  | | The doctor asked me what I wanted him to do today | **6-point Likert scale**  Strongly disagree  Strongly agree | Patient or Observer | 57 |
|  |  | | Asked about treatments patient would want | **11-point Likert scale**  The very worst I could imagine  The very best I could imagine  (Or “Doctor didn’t do” or “I don’t know”) | Clinician | 67 |
|  |  | | Asking about the things in life that are important to you | **11-point Likert scale**  The very worst  The very best | Patient | 15 |
|  |  | | Asking about your spiritual or religious beliefs | **11-point Likert scale**  The very worst  The very best | Patient | 15 |
|  |  | | Asking how much information the patient would like to know | **4-point Likert scale**  Not at all  Yes | Observer | 16 |
|  |  | | My doctor wanted to know exactly how I want to be involved in making the decisions | **5-point Likert scale**  Completely disagree  Completely agree | Patient | 1 |
|  |  | | My doctor asked me which option I prefer | **5-point Likert scale**  Completely disagree  Completely agree | Patient | 1 |
|  |  | | The health professionals asked for my views about the options | **5-point Likert scale**  Strongly disagree  Strongly agree | Patient | 69 |
|  |  | | When I receive prescriptions from my pharmacist, HCP asks me my opinion about how I think my medication regimen is working for me | 1 = Never  5 = Always  (Or “Not applicable”) | Patient | 75 |
|  |  | | Did the provider ask the respondent what he/she wanted? | No  Yes | Patient | 77 |
|  |  | | How well did this doctor ask about the kinds of treatments your loved one would want if they could speak for themselves? | **11-point Likert scale**  Very worst  Very best | Proxy/Caregiver | 90 |
|  |  | | How much did your breast cancer doctor ask you for your input or opinion about which treatment you preferred? | **4-point Likert scale**  Not at all  A great deal | Patient | 94 |
|  |  | | Provider asked what you thought was best for you regarding | No  Yes | Patient | 104 |
|  |  | | The doctor talked to me about things that were important to me | **5-point Likert scale**  Strongly disagree  Strongly agree | Patient | 106 |
|  |  | | The clinician makes an effort to elicit the patient's preferences in response to the options that have been described If the patient declares their preference(s), the clinician is supportive | 0 = No effort  1 = Minimal effort  2 = Moderate effort  3 = Skilled effort  4 = Exemplary effort | Observer | 116 |
|  |  | | Your physician asked you what you want to know about your treatment | **6-point Likert scale**  1 = Strongly disagree  6 = Strongly agree | Patient and Clinician | 118 |
|  |  | | Get you to state which choice or option you prefer | 1-100  (Higher = Better) | Patient | 136 |
|  |  | | Physician asked preferred choice | **5-point Likert scale** | Observer | 142 |
|  | **Understanding** | | This doctor clearly understands my health needs | **5-point Likert scale**  Strongly disagree  Strongly agree | Patient | 21 |
|  |  | | Physician understood what is important to me | **5-point Likert scale**  Strongly disagree  Strongly agree | Patient | 13 |
|  |  | | The physician understood my needs and problems, and took them seriously | **5-point Likert scale**  I do not agree  I fully agree | Patient | 66 |
|  | **Checking** | | Checking the patients’ expectations | **4-point Likert scale**  Not at all  Yes | Observer | 16 |
|  |  | | Checking patients’ preferences for treatment | **4-point Likert scale**  Not at all  Yes | Observer | 16 |
| **Making human connection** | |  |  |  |  |  |
|  | **Caring** | | My doctor is usually considerate of my needs and puts them first | **5-point Likert scale**  Strongly disagree  Strongly agree | Patient | 72 |
|  |  | | My doctor is usually considerate of my needs and puts them first | **5-point Likert scale**  Strongly disagree  Strongly agree | Patient | 110 |
|  | **Sympathizing** | | When I receive prescriptions from my pharmacist, HCP shows concerns and attention to my medication needs | 1 = Never  5 = Always  (Or “Not applicable”) | Patient | 75 |
|  | **Respecting** | | Respecting the things in your life that are important to you | **11-point Likert scale**  The very worst  The very best | Patient | 15 |
|  |  | | Respecting your spiritual or religious beliefs | **11-point Likert scale**  The very worst  The very best | Patient | 15 |
| **Giving attention** | |  |  |  |  |  |
|  | **Showing interest** | | Interested in my ideas about my health | Poor  Fair  Good  Very good  Excellent | Proxy/Caregiver | 6 |
|  |  | | Was interested in what I wanted to know | **7-point Likert scale**  Very strongly disagree  Very strongly agree | Patient | 19 |
|  |  | | Was interested in what I wanted done | **7-point Likert scale**  Very strongly disagree  Very strongly agree | Patient | 19 |
|  |  | | Was interested in what treatment I wanted | **7-point Likert scale**  Very strongly disagree  Very strongly agree | Patient | 19 |
|  |  | | He/she was interested in what I want from care | **5-point Likert scale**  1 = Not at all  5 = Very much | Patient | 64 |
|  |  | | He/she was interested in what I expect from care | **5-point Likert scale**  1 = Not at all  5 = Very much | Patient | 64 |
|  |  | | HCP expresses interest to help me deal with my medication concerns and meet my healthcare needs | 1 = Never  5 = Always  (Or “Not applicable”) | Patient | 75 |
|  | **Listening** | | How much effort was made to listen to the things that matter most to you about your health issues? | 0 = No effort was made  9 = Every effort was made | Patient | 33 |
|  | **Noticing** | | She/he was attentive towards my needs | **10-point Likert scale**  Completely disagree  Completely agree | Patient | 108 |
|  |  | | She/he was attentive towards my needs | **10-point Likert scale**  Completely disagree  Completely agree | Patient | 108 |
| **Self-efficacy building** | |  |  |  |  |  |
|  | **Supporting** | | I received help when I needed it | **5-point Likert scale**  Totally disagree  Totally agree | Patient | 44 |
|  |  | | Helped to set specific goals to improve my eating or exercise | **5-point Likert scale**  Almost never  Generally not  Sometimes  Most of the time  Almost always | Patient | 14 |

**F3 Dimension 3: Patient lives**

|  |  | | **Item** | | | **Responses** | **Respondent** | **Instrument ID#** |
| --- | --- | --- | --- | --- | --- | --- | --- | --- |
| **Providing information** | | | |  |  |  |  |  |
|  | **Informing** | | Impact of treatment on activities of daily living | | | **4-point Likert scale**  Not at all  Yes | Observer | 16 |
|  |  | | Possibilities to do pleasant things during treatment period | | | **4-point Likert scale**  Not at all  Yes | Observer | 16 |
|  |  | | Managing fatigue | | | **4-point Likert scale**  Not at all  Yes | Observer | 16 |
|  |  | | Consequences of treatment for patients’ daily life | | | **4-point Likert scale**  Not at all  Not really  On the whole, yes  Yes | Observer | 16 |
|  |  | | Influence on sexuality | | | **4-point Likert scale**  Not at all  Not really  On the whole, yes  Yes | Observer | 16 |
|  |  | | Possibilities to continue work life or leisure during treatment | | | **4-point Likert scale**  Not at all  Not really  On the whole, yes  Yes | Observer | 16 |
|  |  | | Discussing how your problem or treatment impacts on your daily life | | | **5-point Likert scale**  Poor  Fair  Good  Very good  Excellent | Patient | 96 |
| **Gathering/having needed information** | |  |  | | |  |  |  |
|  | **Exploring** | | Did the doctors ask if you might have any problems actually doing the recommended treatment (for example taking the medication correctly)? | | | **5-point Likert scale** | Patient | 7 |
|  |  | | Asked how my chronic illness affects my life | | | **5-point Likert scale**  Almost never  Generally not  Sometimes  Most of the time  Almost always | Patient | 14 |
|  |  | | How much did your doctor ask whether your medical problem was interfering with the rest of your life? | | | **5-point Likert scale**  Lowest satisfaction  Highest satisfaction | Patient | 52 |
|  |  | | The doctor inquired about how my health was affecting my daily life | | | **6-point Likert scale**  Strongly disagree  Strongly agree | Patient or Observer | 57 |
|  |  | | The doctor asked about how my illness affects my everyday life | | | **5-point Likert scale**  I do not agree  I fully agree | Patient | 66 |
|  |  | | Explore “context”: How will the decision impact the patient’s daily life? | | | Absence  Presence | Observer | 102 |
|  |  | | Ask if there are things make it hard to take care of your health | | | No  Yes | Patient | 104 |
|  |  | | Asks about what is happening in my daily life | | | **7-point Likert scale**  1 = Strongly disagree  7 = Strongly agree | Patient | 117 |
|  | **Understanding** | | Did the doctors understand the kinds of problems you might have in doing the recommended treatment? | | | **5-point Likert scale** | Patient | 7 |
|  | **Checking** | | Checked to see if the treatment plan(s) was acceptable to me | | | **5-point Likert scale**  Strongly disagree  Strongly agree | Patient and Clinician | 47 |
|  |  | | Checked to see if the treatment plan(s) was acceptable to you | | | **5-point Likert scale**  1 = Strongly disagree  2 = Disagree  3 = Neither agree nor disagree  4 = Agree  5 = Strongly agree | Patient | 119 |
| **Making human connection** | |  |  | | |  |  |  |
|  | **Caring** | | How concerned was the doctor about your quality of life? | | | 0 = Never/Not at all  10 = Always/Completely | 43 | 43 |
|  |  | | Degree to which the medical staff cared about the medication's effects after I took the medication | | | **7-point Likert scale**  Very dissatisfied  Very satisfied | 87 | 87 |
|  |  | | Degree to which the medical staff cared about the side effects of drug therapy after I took the medication | | | **7-point Likert scale**  Very dissatisfied  Very satisfied | 87 | 87 |
| **Giving attention** | |  |  | | |  |  |  |
|  | **Showing interest** | | Was interested in the effect of the problem on everyday activities | | | **7-point Likert scale**  Very strongly disagree  Very strongly agree | Patient | 19 |
|  |  | | The consultation: Attention given to quality of life | | | **4-point Likert scale**  Very dissatisfied  Very satisfied  (and option “no opinion or not concerned”) | Patient | 81 |
|  | **Noticing** | | Did staff pay attention to any possible emotional impact of fertility problems? | | | Does not apply/I do not know  No, none at all  Some  Much  Yes, absolutely | Patient | 58 |
| **Self-Efficacy building** | |  |  | | |  |  |  |
|  | **Praising** | | How much did your doctor let you know if he/she was pleased with your efforts to cope with your health problems? | | | **5-point Likert scale**  Lowest satisfaction  Highest satisfaction | Patient | 52 |
|  | **Supporting** | | The doctor gave me some tips to help me work my treatment into my daily routine | | | No  Yes  N/A | Patient | 36 |
|  |  | | Helping you to feel well so that you can perform your normal daily activities? | | | **5-point Likert scale**  Poor  Excellent | Patient | 23 |
|  |  | | Helping you deal with emotional problems related to your health status? | | | **5-point Likert scale**  Poor  Excellent | Patient | 23 |
|  |  | | Helped to make a treatment plan that I could do in my daily life | | | **5-point Likert scale**  Almost never  Generally not  Sometimes  Most of the time  Almost always | Patient | 14 |

**F4 Dimension 4: Patients’ loved ones and social networks**

|  |  | | **Item** | | **Responses** | **Respondent** | **Instrument ID#** |
| --- | --- | --- | --- | --- | --- | --- | --- |
| **Facilitating patient involvement** | | |  |  |  |  |  |
|  | **Involving** | | Did staff also involve your partner? | | No, my partner never accompanied me  No, none at all  Some  Much  Yes, absolutely | Patient | 58 |
|  |  | | Including your loved ones in decisions about your illness and treatment | | **11-point Likert scale**  The very worst  The very best | Patient | 15 |
|  |  | | Spoke to my attendant when explaining the treatment to me | | **5-point Likert scale**  Strongly disagree  Strongly agree | Patient | 112 |
|  | **Encouraging** | | I was encouraged to use self-help/support groups | | **5-point Likert scale**  Strongly disagree  Strongly agree | Patient | 45 |
|  |  | | Encouraged to go to a specific group or class to help me cope with my chronic illness | | **5-point Likert scale**  Almost never  Generally not  Sometimes  Most of the time  Almost always | Patient | 14 |
|  |  | | Encouraged to attend programs in the community that could help me | | **5-point Likert scale**  Almost never  Generally not  Sometimes  Most of the time  Almost always | Patient | 14 |
|  | **Allowing** | | Provide opportunities for the entire family to obtain information | | **7-point Likert scale**  Not at all perceived  Perceived to a very great extent | Proxy/Caregiver | 25 |
| **Adjusting to individual patient** | |  |  | |  |  |  |
|  | **Tailoring (general)** | | Adapting to the needs and wishes of significant others | | **4-point Likert scale**  Not at all  Not really  On the whole, yes  Yes | Observer | 16 |
| **Providing information** | |  |  | |  |  |  |
|  | **Informing** | | I was given adequate information about support services in the community | | **5-point Likert scale**  Definitely not true  Completely true | Patient | 32 |
|  |  | | Give you information about the types of services offered at the organization or in your community | | **7-point Likert scale**  Not at all perceived  Perceived to a very great extent | Proxy/Caregiver | 25 |
|  |  | | Provide advice on how to get information or to contact other parents | | **7-point Likert scale**  Not at all perceived  Perceived to a very great extent | Proxy/Caregiver | 25 |
|  |  | | Did the doctors or nurses give your family or someone close to you all the information they needed to help you recover? | | 1 = Yes completely  2 = Yes to some extent  3 = No | Patient and Caregiver | 63 |
|  |  | | Family not given information needed to help recovery | | 1 = Yes completely  2 = Yes to some extent  3 = No | Patient and Caregiver | 63 |
|  |  | | Talking with your loved ones about what your dying might be like | | **11-point Likert scale**  The very worst  The very best | Patient | 15 |
|  |  | | Support from other patients or support groups | | **4-point Likert scale**  Not at all  Yes | Observer | 16 |
|  |  | | How to get emotional support from others | | **4-point Likert scale**  Not at all  Not really  On the whole, yes  Yes | Observer | 16 |
|  |  | | Discussing how significant others might provide emotional support | | **4-point Likert scale**  Not at all  Not really  On the whole, yes  Yes | Observer | 16 |
|  |  | | Were the topics below discussed with the radiotherapist?: social support | | 0 = Not applicable  0 = No  1 = More or less  2 = Yes | Patient | 88 |
|  | **Addressing** | | Answered family's questions about illness/treatment | | **11-point Likert scale**  The very worst I could imagine  The very best I could imagine  (Or “Doctor didn’t do” or “I don’t know”) | Clinician | 67 |
| **Gathering/having needed information** | |  |  | |  |  |  |
|  | **Exploring** | | How often did the doctor ask you how you were coping with [patient’s] illness? | | 0 = Never/Not at all  10 = Always/Completely | Proxy/Caregiver | 43 |
|  |  | | Exploring questions of significant others | | **4-point Likert scale**  Not at all  Not really  On the whole, yes  Yes | Observer | 16 |
|  |  | | Exploring support needs of significant others | | **4-point Likert scale**  Not at all  Not really  On the whole, yes  Yes | Observer | 16 |
|  |  | | Assess desire for other’s input: Who else would the patient like to consult? | | Absence  Presence | Observer | 102 |
|  | **Understanding** | | This doctor knows a lot about the rest of my family | | **5-point Likert scale**  Strongly disagree  Strongly agree | Patient | 21 |
| **Giving attention** | |  |  | |  |  |  |
|  | **Showing interest** | | Was interested in the effect of the problem on my family or personal life | | **7-point Likert scale**  Very strongly disagree  Very strongly agree | Patient | 19 |
|  | **Noticing** | | My family were given enough attention | | **5-point Likert scale**  Totally disagree  Totally agree | Patient | 44 |
|  |  | | Being attentive to significant others | | **4-point Likert scale**  Not at all  Yes | Observer | 16 |
| **Self-efficacy building** | |  |  | |  |  |  |
|  | **Praising** | | The doctor made me feel I have done a good job caring for my child | | **5-point Likert scale** | Caregiver | 139 |
|  | **Supporting** | | During this hospital stay, did doctors, nurses or other hospital staff talk with you about whether you would have the help you needed when you left the hospital? (Yes/No) | | No  Yes | Patient | 12 |
|  |  | | Helped family decide treatments the patient would want | | **11-point Likert scale**  The very worst I could imagine  The very best I could imagine  (Or “Doctor didn’t do” or “I don’t know”) | Clinician | 67 |
|  |  | | Discussing how patient and significant others can cope with treatment together | | **4-point Likert scale**  Not at all  Not really  On the whole, yes  Yes | Observer | 16 |
|  |  | | How well did this doctor help your family decide about the treatments your loved one would want? | | **11-point Likert scale**  Very worst  Very best | Proxy/Caregiver | 90 |

**F5 Dimension 5: Patient-clinician collaboration (content)**

|  |  | | **Item** | | **Responses** | **Respondent** | **Instrument ID#** |
| --- | --- | --- | --- | --- | --- | --- | --- |
| **Facilitating patient involvement** | | |  |  |  |  |  |
|  | **Involving** | | Discussing options with you; asking your opinion; offering choices and letting you help decide what to do; asking what you think before telling you what to do | | **5-point Likert scale**  Poor  Excellent | patient | 18 |
|  |  | | Practitioner and patient exchange ideas about how the patient could change current behavior | | **5-point Likert scale**  0 = Not at all  1 = Minimally  2 = To some extent  3 = A good deal  4 = A great extent | Observer | 31 |
|  |  | | The care professional talked to me about what I believed would be successful | | **4-point Likert scale**  No  Yes | Patient | 20 |
|  |  | | Doctor communication categories - Recommendations and directives: 2a Recommendations and directives; 2b Directing interaction | | Missing | Observer | 22 |
|  |  | | I was satisfied by the way the doctor/nurse reached the decision about my treatment | | **5-point Likert scale**  Strongly disagree  Strongly agree | Patient | 37 |
|  |  | | Involving you in decisions about your medical care? | | **5-point Likert scale**  Poor  Excellent | Patient | 23 |
|  |  | | Involving you in decisions about your care | | **5-point Likert scale**  Very poor  Very good | Patient | 41 |
|  |  | | I participated in planning my discharge | | **5-point Likert scale**  Strongly disagree  Strongly agree | Patient | 45 |
|  |  | | Both I and my doctor or therapist from the community were actively involved in my hospital treatment plan | | **5-point Likert scale**  Strongly disagree  Strongly agree | Patient | 45 |
|  |  | | Did the doctors try to involve you or include you in decisions about your treatment? | | **5-point Likert scale** | Patient | 7 |
|  |  | | Did the doctors ask how you felt about different treatments? | | **5-point Likert scale** | Patient | 7 |
|  |  | | Satisfied with involvement in decision making | | **5-point Likert scale**  Strongly disagree  Strongly agree | Patient | 13 |
|  |  | | satisfied with physician's involvement | | **5-point Likert scale**  Strongly disagree  Strongly agree | Patient | 13 |
|  |  | | Discussed treatment options with me | | **5-point Likert scale**  Strongly disagree  Strongly agree | Patient and Clinician | 47 |
|  |  | | My HCP doesn’t like to spend time talking about treatment options | | 1 = All the time  5 = Never | Patient | 49 |
|  |  | | Provide opportunities for you to make decisions about treatment | | **7-point Likert scale**  Not at all perceived  Perceived to a very great extent | Proxy/Caregiver | 25 |
|  |  | | Involving you in decisions about your care | | Doesn’t apply  Very poor  Poor  Neither good nor poor  Good  Very good | Patient | 50 |
|  |  | | Talking through the different options for your treatment helping you choose; not rushing ahead or telling you what to do | | Can’t say  Poor  Fair  Good  Very good  Excellent | Patient | 53 |
|  |  | | My doctor discusses options with me; asks my opinions; offers choices and lets me help decide what to do; asks me what I think before telling me what to do | | **4-point Likert scale**  Poor  Excellent | Patient | 54 |
|  |  | | The doctor gave me the responsibility for deciding on how to deal with my health problem | | **6-point Likert scale**  Strongly disagree  Strongly agree | Patient or Observer | 57 |
|  |  | | Was decision-making shared with you, if you preferred? | | No, none at all  Some  Much  Yes, absolutely | Patient | 58 |
|  |  | | Important decisions were made over my head | | Disagree completely  Disagree  So so  Agree  Agree completely | Patient | 61 |
|  |  | | The doctor asked me whether I agreed with his/her recommendations | | **5-point Likert scale**  Very strong disagreement with the statement  Very strong agreement with the statement | Patient | 11 |
|  |  | | Not sufficiently involved in decisions about treatment and care | | 1 = Yes completely  2 = Yes to some extent  3 = No | Patient and Caregiver | 63 |
|  |  | | The physician and I made all treatment decisions together | | **5-point Likert scale**  I do not agree  I fully agree | Patient | 66 |
|  |  | | My doctor and I thoroughly weighed the different treatment options | | **5-point Likert scale**  Completely disagree  Completely agree | Patient | 1 |
|  |  | | The physician involved me in making decisions about treatment | | Disagree  Agree | Patient | 73 |
|  |  | | I work with my HCP to manage my medication(s) | | 1 = Never  5 = Always  (Or “Not applicable”) | Patient | 75 |
|  |  | | Was the respondent offered a choice? | | No  Yes | Patient | 77 |
|  |  | | Involvement in decisions concerning tests and treatments | | **5-point Likert scale**  Negative  Positive | Patient | 79 |
|  |  | | Involving you in decisions about your care | | **5-point Likert scale**  Extremely bad  Extremely good | Patient | 86 |
|  |  | | Shared decision making | | **5-point Likert scale**  Not at all  To a great extent | Observer | 95 |
|  |  | | Letting you help decide what to do | | **5-point Likert scale**  Poor  Fair  Good  Very good  Excellent | Patient | 96 |
|  |  | | Does your HIV provider involve you in decisions about your care? | | A lot less than you wanted  Less than you wanted  Almost as much as you wanted  As much as you wanted | Patient | 100 |
|  |  | | If there were a choice between treatments, how often would this doctor ask you to help make the decision? | | 0 = Never  4 = Very often | Patient | 103 |
|  |  | | How often does this doctor give you some control over your treatment? | | 0 = Never  4 = Very often | Patient | 103 |
|  |  | | How often does this doctor ask you to take some of the responsibility for your treatment | | 0 = Never  4 = Very often | Patient | 103 |
|  |  | | The physician includes me in planning the treatment | | **4-point Likert scale**  Not at all  Very much | Patient and Clinician | 105 |
|  |  | | The doctor involved me in making decisions | | **5-point Likert scale**  I disagree completely  I agree completely | Patient | 113 |
|  |  | | Care provider’s efforts to include you in decisions about your treatment | | **5-point Likert scale** | Patient | 17 |
|  |  | | Offer choices in medical care | | 1-100  (Higher = Better) | Patient | 136 |
|  |  | | Physician included you in treatment decisions | | **5-point Likert scale** | Observer | 140 |
|  |  | | Physician asked you to take responsibility for your treatment | | **5-point Likert scale** | Observer | 141 |
|  |  | | Physician asked you to help make decisions | | **5-point Likert scale** | Observer | 141 |
|  |  | | Physician gives some control over treatment decisions | | **5-point Likert scale** | Observer | 141 |
|  |  | | Physician offered choices in your medical care | | **5-point Likert scale** | Observer | 142 |
|  | **Co-creating** | | Making a plan of action with you (discussing the options, involving you in decisions as much as you want to be involved; not ignoring your views) | | **5-point Likert scale**  Poor  Excellent | Patient | 4 |
|  |  | | Discussed and reached agreement with me on the plan of treatment | | **7-point Likert scale**  Very strongly disagree  Very strongly agree | Patient | 19 |
|  |  | | My doctor and I agreed about which treatment (or no treatment) was best for me | | **5-point Likert scale**  Strongly disagree  Strongly agree | Patient | 2 |
|  |  | | The care professional set goals and plans for the care process in collaboration with me | | **4-point Likert scale**  No  Yes | Patient | 20 |
|  |  | | I was able to participate in the planning of my care | | **5-point Likert scale**  Totally disagree  Totally agree | Patient | 44 |
|  |  | | Plan together so they are all working in the same direction | | **7-point Likert scale**  Not at all perceived  Perceived to a very great extent | Proxy/Caregiver | 25 |
|  |  | | He/she offered me the opportunity to discuss and decide together the “things to do” | | **5-point Likert scale**  1 = Not at all  5 = Very much | Patient | 64 |
|  |  | | My doctor and I selected a treatment option together | | **5-point Likert scale**  Completely disagree  Completely agree | Patient | 1 |
|  |  | | My doctor and I reached an agreement on how to proceed | | **5-point Likert scale**  Completely disagree  Completely agree | Patient | 1 |
|  |  | | I felt the health professional and I agreed on which option was the best one for me | | **5-point Likert scale**  Strongly disagree  Strongly agree | Patient | 69 |
|  | **Encouraging** | | Encouraging you to ask questions; answering them clearly; never avoiding your questions or lecturing you | | **5-point Likert scale**  Poor  Excellent | patient | 18 |
|  |  | | Practitioner invites the patient to talk about behavior change | | **5-point Likert scale**  0 = Not at all  1 = Minimally  2 = To some extent  3 = A good deal  4 = A great extent | Observer | 31 |
|  |  | | Practitioner encourages patient to talk about change | | **5-point Likert scale**  0 = Not at all  1 = Minimally  2 = To some extent  3 = A good deal  4 = A great extent | Observer | 31 |
|  |  | | Encouraged me to ask questions | | Poor  Fair  Good  Very good  Excellent | Proxy/Caregiver | 6 |
|  |  | | Encouraging the patient to ask questions | | **7-point Likert scale**  Strongly disagree  Strongly agree | Clinician | 46 |
|  |  | | Encouraged me to ask questions | | **5-point Likert scale**  Strongly disagree  Strongly agree | Patient and Clinician | 47 |
|  |  | | My HCP encourages me to give my opinion about my medical treatment | | 1 = All the time  5 = Never | Patient | 49 |
|  |  | | My doctor encouraged me to tell him/her everything I thought important | | **5-point Likert scale**  Lowest satisfaction  Highest satisfaction | Patient | 52 |
|  |  | | My provider encouraged me to tell him/her everything I thought important | | **5-point Likert scale**  1 = Very little agreement  5 = Very much in agreement | Patient | 56 |
|  |  | | My doctor encouraged me to give my opinion about treatment | | **5-point Likert scale**  Very strong disagreement with the statement  Very strong agreement with the statement | Patient | 11 |
|  |  | | Encouraging you to ask questions | | **5-point Likert scale**  Poor  Fair  Good  Very good  Excellent | Patient | 96 |
|  |  | | My doctor encouraged me to give my opinion about my medical treatment | | **5-point Likert scale**  Strongly disagree  Strongly agree | Patient | 110 |
|  |  | | Encouraged me to ask questions | | **5-point Likert scale**  Strongly disagree  Strongly agree | Patient | 112 |
|  |  | | Encouraged you to ask questions | | **5-point Likert scale**  1 = Strongly disagree  2 = Disagree  3 = Neither agree nor disagree  4 = Agree  5 = Strongly agree | Patient | 119 |
|  |  | | Sharing (how, and if, the surgeon promoted shared decision making and patient participation throughout the visit) | | 1-5  (Higher = Better) | Observer | 135 |
|  | **Allowing** | | Did the healthcare provider give you/your child a chance to ask questions about care? | | No  Yes somewhat  Yes completely | Patient or Caregiver | 30 |
|  |  | | Did the RTT give you the opportunity to talk about aspects related to your therapy? | | **5-point Likert scale**  Not at all  Very much | Patient | 34 |
|  |  | | The doctor gave me a chance to be involved in the decisions during the consultation | | **5-point Likert scale**  Strongly disagree  Strongly agree | Patient | 2 |
|  |  | | The clinician offers the patient explicit opportunities to ask questions during the decision making process | | 0 = The behaviour is not observed  4 = The behaviour is exhibited to a very high standard | Observer | 8 |
|  |  | | How much did your doctor ask questions that allowed more than a “yes” or “no” answer? | | **5-point Likert scale**  Lowest satisfaction  Highest satisfaction | Patient | 52 |
|  |  | | How often were you given the opportunity to ask your physician questions? | | Never  Sometimes  Usually  Always | Patient | 58 |
|  |  | | He/she asked questions that allowed me to express my view | | **5-point Likert scale**  1 = Not at all  5 = Very much | Patient | 64 |
|  |  | | I felt it would be OK to choose any option we talked about | | **5-point Likert scale**  Strongly disagree  Strongly agree | Patient | 69 |
|  |  | | Did you have the opportunity to ask for more information if you wanted to? | | No  Yes | Patient | 83 |
|  |  | | The doctor gave you appropriate opportunity to ask questions about your treatment | | **5-point Likert scale**  Strongly disagree  Strongly agree | Patient | 89 |
|  |  | | Opportunities for me to ask questions | | **7-point Likert scale**  Strongly disagree  Strongly agree | Patient | 107 |
|  |  | | Willing to let me ask questions during visit | | **7-point Likert scale**  Strongly disagree  Strongly agree | Patient | 107 |
|  |  | | My doctor gave me opportunity to ask questions | | **5-point Likert scale**  Strongly disagree  Strongly agree | Patient | 110 |
|  |  | | Are you satisfied that the family doctor allows you to give an opinion about your treatment? | | **5-point Likert scale**  Not satisfied  Completely satisfied | Patient | 111 |
|  |  | | The doctor gave me plenty of opportunities to ask questions | | **5-point Likert scale**  I disagree completely  I agree completely | Patient | 113 |
|  |  | | Your physician provided you the possibility to ask questions | | **6-point Likert scale**  1 = Strongly disagree  6 = Strongly agree | Patient and Clinician | 118 |
| **Adjusting to individual patient** | |  |  | |  |  |  |
|  | **Tailoring (general)** | | The staff relied on my own assessment of how I felt | | **5-point Likert scale**  Totally disagree  Totally agree | Patient | 44 |
| **Providing information** | |  |  | |  |  |  |
|  | **Informing** | | Explaining things clearly (fully answering your questions, explaining clearly, giving you adequate information; not being vague) | | **5-point Likert scale**  Poor  Excellent | Patient | 4 |
|  |  | | Was careful to explain the plan of treatment | | **7-point Likert scale**  Very strongly disagree  Very strongly agree | Patient | 19 |
|  |  | | Treatment choices were fully explained to me | | **5-point Likert scale**  Definitely not true  Completely true | Patient | 32 |
|  |  | | The doctor made me aware of the different treatments available | | **5-point Likert scale**  Strongly disagree  Strongly agree | Patient | 2 |
|  |  | | The doctor gave me enough information about the treatment choices available | | **5-point Likert scale**  Strongly disagree  Strongly agree | Patient | 2 |
|  |  | | The doctor gave enough explanation of the information about treatment choices | | **5-point Likert scale**  Strongly disagree  Strongly agree | Patient | 2 |
|  |  | | This doctor told me everything about my treatment | | **5-point Likert scale**  Strongly disagree  Strongly agree | Patient | 35 |
|  |  | | The care professional provided information about different possibilities for care | | **4-point Likert scale**  No  Yes | Patient | 20 |
|  |  | | The care professional provided information about other types of care or support | | **4-point Likert scale**  No  Yes | Patient | 20 |
|  |  | | This doctor always explains things to my satisfaction | | **5-point Likert scale**  Strongly disagree  Strongly agree | Patient | 21 |
|  |  | | The doctor discussed with me what might be the cause | | No  Yes  N/A | Patient | 36 |
|  |  | | The doctor gave me clear instructions about my treatment: what to do, when, how often, and for how long | | No  Yes  N/A | Patient | 36 |
|  |  | | The doctor told me what I might expect when taking my medication/treatment | | No  Yes  N/A | Patient | 36 |
|  |  | | The doctor told me how to monitor my problem to see if the treatment is working | | No  Yes  N/A | Patient | 36 |
|  |  | | Doctor communication categories - Information giving: Technical information | | Missing | Observer | 22 |
|  |  | | Patient communication categories - Offering additional information (NB where not coded as assertive response) | | Missing | Observer | 22 |
|  |  | | Adequacy of explanations to patient | | **5-point Likert scale**  1 = Poor  5 = Excellent | Patient | 5 |
|  |  | | Thoroughness? | | **5-point Likert scale**  Poor  Excellent | Patient | 23 |
|  |  | | Explaining the purpose of tests and treatments? | | **5-point Likert scale**  Poor  Excellent | Patient | 23 |
|  |  | | Preparing you for what to expect from specialist or hospital care? | | **5-point Likert scale**  Poor  Excellent | Patient | 23 |
|  |  | | During this visit (or previous visits) has a provider talked to you about nutrition or what is good for you to be eating during your pregnancy? | | Don’t know  No  Yes, previous visit only  Yes, this and previous visit  Yes, this visit only | Patient | 39 |
|  |  | | Amount of explanation you received about the problem or treatment | | No  Yes | Patient | 39 |
|  |  | | My doctor explains all the treatment options to me so I can make an informed choice | | **6-point Likert scale**  None  All of the time | Patient | 40 |
|  |  | | Explaining tests and treatments | | **5-point Likert scale**  Very poor  Very good | Patient | 41 |
|  |  | | Before giving you any new medications, how often did hospital staff tell you what the medications were for? | | **4-point Likert scale**  Never  Sometimes  Usually  Always | Patient | 12 |
|  |  | | I received enough information about my medication | | **5-point Likert scale**  Totally disagree  Totally agree | Patient | 44 |
|  |  | | I received enough information about my home care | | **5-point Likert scale**  Totally disagree  Totally agree | Patient | 44 |
|  |  | | I was given the clear instructions about home care | | **5-point Likert scale**  Totally disagree  Totally agree | Patient | 44 |
|  |  | | I was given information about how to manage my medication side effects | | **5-point Likert scale**  Strongly disagree  Strongly agree | Patient | 45 |
|  |  | | Did the doctors explain why a test was being done? | | **5-point Likert scale** | Patient | 7 |
|  |  | | Did the doctors explain how the test was done? | | **5-point Likert scale** | Patient | 7 |
|  |  | | Did the doctors tell you what you could do to take care of yourself at home? | | **5-point Likert scale** | Patient | 7 |
|  |  | | Did the doctors tell you how to pay attention to your symptoms and when to call the doctor? | | **5-point Likert scale** | Patient | 7 |
|  |  | | Did the doctors clearly explain how to take the medicine(that is when, how much and for how long)? | | **5-point Likert scale** | Patient | 7 |
|  |  | | Did the doctors give you written instruction about how to take the medicine (other than what was on the container)? | | **5-point Likert scale** | Patient | 7 |
|  |  | | Did the doctors tell you the reason for taking each medicine? | | **5-point Likert scale** | Patient | 7 |
|  |  | | Did the doctors tell you about side effects you might get from your medicine? | | **5-point Likert scale** | Patient | 7 |
|  |  | | The doctor’s explanations of things to me were | | **5-point Likert scale**  Poor  Excellent | Patient | 24 |
|  |  | | The treatment for his or her medical problem | | **7-point Likert scale**  Strongly disagree  Strongly agree | Clinician | 46 |
|  |  | | The advantages and disadvantages of treatment options | | **7-point Likert scale**  Strongly disagree  Strongly agree | Clinician | 46 |
|  |  | | The purpose of any tests that were needed | | **7-point Likert scale**  Strongly disagree  Strongly agree | Clinician | 46 |
|  |  | | How prescribed medication will help his or her problem | | **7-point Likert scale**  Strongly disagree  Strongly agree | Clinician | 46 |
|  |  | | How to take prescribed medication | | **7-point Likert scale**  Strongly disagree  Strongly agree | Clinician | 46 |
|  |  | | The possible side effects of the medication | | **7-point Likert scale**  Strongly disagree  Strongly agree | Clinician | 46 |
|  |  | | Reviewing, or repeating, important information for the patient | | **7-point Likert scale**  Strongly disagree  Strongly agree | Clinician | 46 |
|  |  | | Explained the lab tests needed (eg blood, x-rays, ultrasound, etc) | | **5-point Likert scale**  Strongly disagree  Strongly agree | Patient and Clinician | 47 |
|  |  | | Explained medications, if any, including possible side-effects | | **5-point Likert scale**  Strongly disagree  Strongly agree | Patient and Clinician | 47 |
|  |  | | The doctor gave me all the information I was expecting to receive about my health | | **5-point Likert scale**  Strongly disagree  Strongly agree | Patient | 48 |
|  |  | | The doctor told me how to care for my condition | | **5-point Likert scale**  Strongly disagree  Strongly agree | Patient | 48 |
|  |  | | My HCP gives me a complete explanation for my medical symptoms or treatment | | 1 = All the time  5 = Never | Patient | 49 |
|  |  | | I give my opinion about the type(s) or test(s) or treatment(s)that my HCP recommended | | 1 = All the time  5 = Never | Patient | 49 |
|  |  | | Provide you with written information about what your child is doing in therapy | | **7-point Likert scale**  Not at all perceived  Perceived to a very great extent | Proxy/Caregiver | 25 |
|  |  | | Fully explain treatment choices to you | | **7-point Likert scale**  Not at all perceived  Perceived to a very great extent | Proxy/Caregiver | 25 |
|  |  | | Give you information about your child that is consistent from person to person | | **7-point Likert scale**  Not at all perceived  Perceived to a very great extent | Proxy/Caregiver | 25 |
|  |  | | Provide you with written information about your child's progress | | **7-point Likert scale**  Not at all perceived  Perceived to a very great extent | Proxy/Caregiver | 25 |
|  |  | | Tell you about the results from assessments | | **7-point Likert scale**  Not at all perceived  Perceived to a very great extent | Proxy/Caregiver | 25 |
|  |  | | Have information available about your child's disability | | **7-point Likert scale**  Not at all perceived  Perceived to a very great extent | Proxy/Caregiver | 25 |
|  |  | | Explaining tests and treatments | | Doesn’t apply  Very poor  Poor  Neither good nor poor  Good  Very good | Patient | 50 |
|  |  | | The doctor/nurse/health visitor told me everything about my treatment/care/ explained the reasons for advice given | | **5-point Likert scale**  Strongly disagree  Strongly agree | Patient | 51 |
|  |  | | The clinician states that there is more than one way to deal with the identified problem (‘equipoise’) | | 0 = The behaviour is not observed  4 = The behaviour is exhibited to a very high standard | Observer | 8 |
|  |  | | The clinician lists ‘options’, which can include the choice of ‘no action’ | | 0 = The behaviour is not observed  4 = The behaviour is exhibited to a very high standard | Observer | 8 |
|  |  | | The clinician explains the pros and cons of options to the patient (taking ‘no action’ is an option) | | 0 = The behaviour is not observed  4 = The behaviour is exhibited to a very high standard | Observer | 8 |
|  |  | | The clinician indicates the need for a decision making (or deferring) stage | | 0 = The behaviour is not observed  4 = The behaviour is exhibited to a very high standard | Observer | 8 |
|  |  | | Given choices about treatment to think about | | **5-point Likert scale**  Almost never  Generally not  Sometimes  Most of the time  Almost always | Patient | 14 |
|  |  | | Given a written list of things I should do to improve my health | | **5-point Likert scale**  Almost never  Generally not  Sometimes  Most of the time  Almost always | Patient | 14 |
|  |  | | Shown how what I did to take care of my illness influenced my condition | | **5-point Likert scale**  Almost never  Generally not  Sometimes  Most of the time  Almost always | Patient | 14 |
|  |  | | Given a copy of my treatment plan | | **5-point Likert scale**  Almost never  Generally not  Sometimes  Most of the time  Almost always | Patient | 14 |
|  |  | | Told how my visits with other types of doctors, like the eye doctor or surgeon, helped my treatment | | **5-point Likert scale**  Almost never  Generally not  Sometimes  Most of the time  Almost always | Patient | 14 |
|  |  | | My doctor told me all I wanted to know about my condition and treatment | | **5-point Likert scale**  Lowest satisfaction  Highest satisfaction | Patient | 52 |
|  |  | | Explaining what he/she is going to do before starting to examine you | | Can’t say  Poor  Fair  Good  Very good  Excellent | Patient | 53 |
|  |  | | Advising you on how to look after your teeth and gums at home | | Can’t say  Poor  Fair  Good  Very good  Excellent | Patient | 53 |
|  |  | | My doctor explains to me during the physical exam about what he/she is about to do and why; tells me what he/she finds | | **4-point Likert scale**  Poor  Excellent | Patient | 54 |
|  |  | | My doctor explains me what I need to know about my problems, how and why they occurred, and what to expect next | | **4-point Likert scale**  Poor  Excellent | Patient | 54 |
|  |  | | The doctor did not fully explain everything to me | | **6-point Likert scale**  Strongly disagree  Strongly agree | Patient or Observer | 57 |
|  |  | | Were different treatment options discussed with you? | | No  Yes, but insufficiently  Yes, absolutely | Patient | 58 |
|  |  | | Were you informed of any possible side-effects of the medication prescribed to you? | | No  Yes, but insufficiently  Yes, absolutely  Does not apply: No medication was prescribed to me | Patient | 58 |
|  |  | | Did the staff inform you how to get support from a social worker or a psychologist? | | No  Yes, but insufficiently  Yes, absolutely | Patient | 58 |
|  |  | | Were the results of the investigations discussed with you? | | No  Yes, but insufficiently  Yes, absolutely | Patient | 58 |
|  |  | | How often did you get contradictory information or advice? | | Never  Sometimes  Usually  Always | Patient | 58 |
|  |  | | The AP explained the reason for treatment | | **5-point Likert scale**  Strongly disagree  Strongly agree | Patient | 59 |
|  |  | | My doctor gave me a complete explanation for medical symptoms and treatment | | **5-point Likert scale**  Very strong disagreement with the statement  Very strong agreement with the statement | Patient | 11 |
|  |  | | I gave my opinion about the types of treatment or procedures the doctor was recommending | | **5-point Likert scale**  Very strong disagreement with the statement  Very strong agreement with the statement | Patient | 11 |
|  |  | | Information exchange: The doctor explained the reason why the treatment was recommended for me | | **4-point Likert scale**  Does not apply  Strongly disagree  Disagree  Agree  Strongly agree | Patient | 62 |
|  |  | | Interpersonal skills: The doctor should have told me more about how to care for my condition | | **4-point Likert scale**  Does not apply  Strongly disagree  Disagree  Agree  Strongly agree | Patient | 62 |
|  |  | | Did a member of staff tell you about medication side effects to watch for when you went home? | | 1 = Yes completely  2 = Yes to some extent  3 = No | Patient and Caregiver | 63 |
|  |  | | Did someone tell you about danger signals regarding your illness or treatment to watch for after you went home? | | 1 = Yes completely  2 = Yes to some extent  3 = No | Patient and Caregiver | 63 |
|  |  | | Purpose of medicines not explained | | 1 = Yes completely  2 = Yes to some extent  3 = No | Patient and Caregiver | 63 |
|  |  | | Not told about medication side effects | | 1 = Yes completely  2 = Yes to some extent  3 = No | Patient and Caregiver | 63 |
|  |  | | Not told about danger signals to look for at home | | 1 = Yes completely  2 = Yes to some extent  3 = No | Patient and Caregiver | 63 |
|  |  | | He/she provided me with clear information | | **5-point Likert scale**  1 = Not at all  5 = Very much | Patient | 64 |
|  |  | | The physician gave me detailed information about the available treatment options | | **5-point Likert scale**  I do not agree  I fully agree | Patient | 66 |
|  |  | | The physician spoke to me in detail about the risks and side-effects of the proposed treatment | | **5-point Likert scale**  I do not agree  I fully agree | Patient | 66 |
|  |  | | The physician gave me detailed information about my illness | | **5-point Likert scale**  I do not agree  I fully agree | Patient | 66 |
|  |  | | Talking to you about the details concerning the possibility that you might get sicker | | **11-point Likert scale**  The very worst  The very best | Patient | 15 |
|  |  | | Talking to you about what dying might be like | | **11-point Likert scale**  The very worst  The very best | Patient | 15 |
|  |  | | Aim of treatment | | **4-point Likert scale**  Not at all  Yes | Observer | 16 |
|  |  | | Discussion of possible side effects of treatment | | **4-point Likert scale**  Not at all  Yes | Observer | 16 |
|  |  | | Preventing or reducing side effects at home | | **4-point Likert scale**  Not at all  Yes | Observer | 16 |
|  |  | | Risk of infections during treatment | | **4-point Likert scale**  Not at all  Yes | Observer | 16 |
|  |  | | How often you need to come to the hospital | | **4-point Likert scale**  Not at all  Yes | Observer | 16 |
|  |  | | What will happen if patient chooses not to start treatment | | **4-point Likert scale**  Not at all  Yes | Observer | 16 |
|  |  | | Necessity of home care during treatment | | **4-point Likert scale**  Not at all  Yes | Observer | 16 |
|  |  | | Expected survival | | **4-point Likert scale**  Not at all  Yes | Observer | 16 |
|  |  | | Order in which medicines are administered | | **4-point Likert scale**  Not at all  Yes | Observer | 16 |
|  |  | | Explaining emotional reactions on chemotherapy treatment | | **4-point Likert scale**  Not at all  Yes | Observer | 16 |
|  |  | | Dietary advice during treatment | | **4-point Likert scale**  Not at all  Yes | Observer | 16 |
|  |  | | Sufficient information about chemotherapy treatment | | **4-point Likert scale**  Not at all  Not really  On the whole, yes  Yes | Observer | 16 |
|  |  | | Length of chemotherapy treatment | | **4-point Likert scale**  Not at all  Not really  On the whole, yes  Yes | Observer | 16 |
|  |  | | Alternative or complementary therapies | | **4-point Likert scale**  Not at all  Not really  On the whole, yes  Yes | Observer | 16 |
|  |  | | How chemotherapy is administered | | **4-point Likert scale**  Not at all  Not really  On the whole, yes  Yes | Observer | 16 |
|  |  | | How to deal with urine, faeces and vomit | | **4-point Likert scale**  Not at all  Not really  On the whole, yes  Yes | Observer | 16 |
|  |  | | Treatments that can reduce side effects | | **4-point Likert scale**  Not at all  Not really  On the whole, yes  Yes | Observer | 16 |
|  |  | | Discussion of all possible side effects of treatment | | **4-point Likert scale**  Not at all  Not really  On the whole, yes  Yes | Observer | 16 |
|  |  | | Explanation of what was done for you | | **5-point Likert scale**  Poor  Excellent | Patient | 28 |
|  |  | | My doctor made clear that a decision needs to be made | | **5-point Likert scale**  Completely disagree  Completely agree | Patient | 1 |
|  |  | | My doctor told me there are different options for treating my medical condition | | **5-point Likert scale**  Completely disagree  Completely agree | Patient | 1 |
|  |  | | My doctor precisely explained the advantages and disadvantages of the treatment options | | **5-point Likert scale**  Completely disagree  Completely agree | Patient | 1 |
|  |  | | The health professionals talked about other options from the one we chose | | **5-point Likert scale**  Strongly disagree  Strongly agree | Patient | 69 |
|  |  | | The health professionals talked about whether or not there was a most effective option for this health problem | | **5-point Likert scale**  Strongly disagree  Strongly agree | Patient | 69 |
|  |  | | The health professionals gave their views about the options | | **5-point Likert scale**  Strongly disagree  Strongly agree | Patient | 69 |
|  |  | | I talked about the risks and benefits of the options for me and my health | | **5-point Likert scale**  Strongly disagree  Strongly agree | Patient | 69 |
|  |  | | Did the dermatologist clearly explain to you the potential side effect of available topical therapies? | | **5-point Likert scale**  1 = Not at all  5 = Yes, fully | Patient | 74 |
|  |  | | Did the dermatologist explain how the treatment works? | | **5-point Likert scale**  1 = Not at all  5 = Yes, fully | Patient | 74 |
|  |  | | Did the dermatologists explain how your health might change after taking your therapy for its full course? | | **5-point Likert scale**  1 = Not at all  5 = Yes, fully | Patient | 74 |
|  |  | | Did the dermatologist explain how to manage your skin disease? | | **5-point Likert scale**  1 = Not at all  5 = Yes, fully | Patient | 74 |
|  |  | | HCP advises me about how to monitor myself for medication side effects | | 1 = Never  5 = Always  (Or “Not applicable”) | Patient | 75 |
|  |  | | HCP advises me about my medication(s) even if I do not have medication questions | | 1 = Never  5 = Always  (Or “Not applicable”) | Patient | 75 |
|  |  | | HCP advises me about whether or not it is okay for me to take my medication(s) with over-the-counter products | | 1 = Never  5 = Always  (Or “Not applicable”) | Patient | 75 |
|  |  | | The health care provider told me that without using a contraceptive method I could get pregnant again | | **4-point Likert scale**  1 = Strongly disagree  4 = Strongly agree | Patient | 76 |
|  |  | | Health care provider has not given me enough information about the care so that I didn’t know what to expect | | **4-point Likert scale**  1 = Strongly disagree  4 = Strongly agree | Patient | 76 |
|  |  | | Were the pros of surgery discussed? | | Not at all  A little  Some  A lot | Patient | 77 |
|  |  | | Were the cons of surgery discussed? | | Not at all  A little  Some  A lot | Patient | 77 |
|  |  | | Clarification of the tests to be taken | | **5-point Likert scale**  Negative  Positive | Patient | 79 |
|  |  | | Clarification of the treatment chosen | | **5-point Likert scale**  Negative  Positive | Patient | 79 |
|  |  | | The treatment: Advices given | | **4-point Likert scale**  Very dissatisfied  Very satisfied  (and option “no opinion or not concerned”) | Patient | 81 |
|  |  | | The treatment: Explanation about treatment | | **4-point Likert scale**  Very dissatisfied  Very satisfied  (and option “no opinion or not concerned”) | Patient | 81 |
|  |  | | Were you satisfied with the way the status of the disease and its treatment was reported? | | No  Yes | Patient | 83 |
|  |  | | Information about follow-up care is provided to me | | No  Yes | Patient | 84 |
|  |  | | Reviewing and explaining tests results | | **5-point Likert scale**  Extremely bad  Extremely good | Patient | 86 |
|  |  | | Instructions on the use and dosage of drugs after discharge | | **7-point Likert scale**  Very dissatisfied  Very satisfied | Patient | 87 |
|  |  | | Instructions on how to take drugs correctly after discharge | | **7-point Likert scale**  Very dissatisfied  Very satisfied | Patient | 87 |
|  |  | | Instructions on what type of drugs should be taken after discharge | | **7-point Likert scale**  Very dissatisfied  Very satisfied | Patient | 87 |
|  |  | | Instructions on matters requiring attention to diet when taking drugs after discharge | | **7-point Likert scale**  Very dissatisfied  Very satisfied | Patient | 87 |
|  |  | | Instructions on how to take medications or use devices (e.g. inhaler) | | **7-point Likert scale**  Very dissatisfied  Very satisfied | Patient | 87 |
|  |  | | Explanation of the purpose of drug therapy provided by the medical staff during the hospital stay | | **7-point Likert scale**  Very dissatisfied  Very satisfied | Patient | 87 |
|  |  | | Explanation I received about the medications' possible side effects | | **7-point Likert scale**  Very dissatisfied  Very satisfied | Patient | 87 |
|  |  | | Explanation provided by the medical staff about drug therapy dose changes during the hospital stay | | **7-point Likert scale**  Very dissatisfied  Very satisfied | Patient | 87 |
|  |  | | Instructions on how to deal with side effects after discharge | | **7-point Likert scale**  Very dissatisfied  Very satisfied | Patient | 87 |
|  |  | | Instructions on matters involving taking two or more types of drugs simultaneously after discharge | | **7-point Likert scale**  Very dissatisfied  Very satisfied | Patient | 87 |
|  |  | | You are satisfied with the explanation about your disease and treatment given by your doctors | | **5-point Likert scale**  Strongly disagree  Strongly agree | Patient | 89 |
|  |  | | The need of giving a medicine and its possible side effects were explained to you well | | **5-point Likert scale**  Strongly disagree  Strongly agree | Patient | 89 |
|  |  | | You were given enough information and instructions for home before undergoing any diagnostic test (e.g., Fasting, drinking lots of or no water, etc | | **5-point Likert scale**  Strongly disagree  Strongly agree | Patient | 89 |
|  |  | | How well did this conference help you understand the choices and decisions that may need to be made? | | **11-point Likert scale**  Very worst  Very best | Proxy/Caregiver | 90 |
|  |  | | The doctor was happy to explain what I didn't understand | | **4-point Likert scale**  Not at all  Very much so | Patient | 91 |
|  |  | | My doctor told me all I wanted to know about my condition and treatment | | Never  Occasionally  Sometimes  Often  Always | Patient | 92 |
|  |  | | Need for procedure explained clearly by resident | | **5-point Likert scale**  Strongly disagree  Strongly agree | Patient and Clinician | 93 |
|  |  | | Resident explained risks and benefits of procedure | | **5-point Likert scale**  Strongly disagree  Strongly agree | Patient and Clinician | 93 |
|  |  | | Resident discussed alternative procedures | | **5-point Likert scale**  Strongly disagree  Strongly agree | Patient and Clinician | 93 |
|  |  | | Discussing options with you and offering choices | | **5-point Likert scale**  Poor  Fair  Good  Very good  Excellent | Patient | 96 |
|  |  | | How well did the doctor explain to you what he or she was doing and why? | | **9-point Likert scale**  Did not explain at all  Explained very well | Patient | 97 |
|  |  | | How clearly did he/she tell you what the plan was? | | **6-point Likert scale**  1 = Poor  6 = Excellent | Patient | 99 |
|  |  | | How clear was the explanation of the goals and procedure of the treatment? | | **6-point Likert scale**  1 = Poor  6 = Excellent | Patient | 99 |
|  |  | | Describe alternatives: What are the clinically reasonable choices? | | Absence  Presence | Observer | 102 |
|  |  | | Discuss pros/cons: What are the pros and cons of relevance to this patient? | | Absence  Presence | Observer | 102 |
|  |  | | Discuss uncertainty: How can I convey the likelihood of success? | | Absence  Presence | Observer | 102 |
|  |  | | Provider talked about reasons to take a medicine | | Not at all  A little  Some  A lot | Patient | 104 |
|  |  | | Provider talked about reasons not to take a medicine | | Not at all  A little  Some  A lot | Patient | 104 |
|  |  | | The physician explains the planned treatment | | **4-point Likert scale**  Not at all  Very much | Patient and Clinician | 105 |
|  |  | | The physician explains the illness and treatment to my family | | **4-point Likert scale**  Not at all  Very much | Patient and Clinician | 105 |
|  |  | | Some information about IBS | | **7-point Likert scale**  Strongly disagree  Strongly agree | Patient | 107 |
|  |  | | In-depth IBS information | | **7-point Likert scale**  Strongly disagree  Strongly agree | Patient | 107 |
|  |  | | Willing to tell me where to get more info | | **7-point Likert scale**  Strongly disagree  Strongly agree | Patient | 107 |
|  |  | | Willing to tell me about IBS studies/drugs | | **7-point Likert scale**  Strongly disagree  Strongly agree | Patient | 107 |
|  |  | | She/he explained the process of labor and delivery | | **10-point Likert scale**  Completely disagree  Completely agree | Patient | 108 |
|  |  | | The CP told me how to take my prescriptions | | Strongly disagree  Strongly agree | Patient | 109 |
|  |  | | The CP told me what to do if I missed a dose | | Strongly disagree  Strongly agree | Patient | 109 |
|  |  | | The CP told me about possible side effects of my prescriptions | | Strongly disagree  Strongly agree | Patient | 109 |
|  |  | | The CP gave me information about my health as well as my prescription medicines | | Strongly disagree  Strongly agree | Patient | 109 |
|  |  | | Are you satisfied with the information the family doctor provides to you about possible drug reactions that can arise during your treatment? | | **5-point Likert scale**  Not satisfied  Completely satisfied | Patient | 111 |
|  |  | | Gave me instructions about the prescribed medications | | **5-point Likert scale**  Strongly disagree  Strongly agree | Patient | 112 |
|  |  | | Told you about the importance of continuing this treatment for glaucoma? | | **5-point Likert scale**  Very dissatisfied  Very satisfied | Patient | 112 |
|  |  | | The doctor gave me the right amount of information about my illness and its treatment | | **5-point Likert scale**  I disagree completely  I agree completely | Patient | 113 |
|  |  | | The doctor explained my condition clearly | | **5-point Likert scale**  I disagree completely  I agree completely | Patient | 113 |
|  |  | | Explanation of treatment | | Very poor  Best | Patient | 114 |
|  |  | | Advice about ways to avoid illness and stay healthy | | Very poor  Best | Patient | 114 |
|  |  | | My doctor(s) and staff explained the benefits of treating my heart disease by taking medicines alone | | **4-point Likert scale**  Not at all  A little  Some  A lot | Patient | 115 |
|  |  | | My doctor(s) and staff explained the risks of treating my heart disease by taking medicines alone | | **4-point Likert scale**  Not at all  A little  Some  A lot | Patient | 115 |
|  |  | | My doctor(s) and staff explained the benefits of treating my heart disease by having an angioplasty (stent procedure) | | **4-point Likert scale**  Not at all  A little  Some  A lot | Patient | 115 |
|  |  | | My doctor(s) and staff explained the risks of treating my heart disease by having an angioplasty (stent procedure) | | **4-point Likert scale**  Not at all  A little  Some  A lot | Patient | 115 |
|  |  | | My doctor(s) and staff explained the benefits of treating my heart disease by having bypass surgery | | **4-point Likert scale**  Not at all  A little  Some  A lot | Patient | 115 |
|  |  | | My doctor(s) and staff explained the risks of treating my heart disease by having bypass surgery | | **4-point Likert scale**  Not at all  A little  Some  A lot | Patient | 115 |
|  |  | | For the health issue being discussed, the clinician draws attention to or confirms that alternate treatment or management options exist or that the need for a decision exists. If the patient rather than the clinician draws attention to the availability of options, the clinician responds by agreeing that the options need deliberation | | 0 = No effort  1 = Minimal effort  2 = Moderate effort  3 = Skilled effort  4 = Exemplary effort | Observer | 116 |
|  |  | | The clinician gives information or checks understanding about the options that are considered reasonable (this can include taking no action), to support the patient in comparing alternatives. If the patient requests clarification, the clinician supports the process | | 0 = No effort  1 = Minimal effort  2 = Moderate effort  3 = Skilled effort  4 = Exemplary effort | Observer | 116 |
|  |  | | Explained the lab tests needed (e.g., blood, X-rays, ultrasound, etc.) | | **5-point Likert scale**  1 = Strongly disagree  2 = Disagree  3 = Neither agree nor disagree  4 = Agree  5 = Strongly agree | Patient | 119 |
|  |  | | Discussed treatment options with you | | **5-point Likert scale**  1 = Strongly disagree  2 = Disagree  3 = Neither agree nor disagree  4 = Agree  5 = Strongly agree | Patient | 119 |
|  |  | | Explained medications, if any, including possible side effects | | **5-point Likert scale**  1 = Strongly disagree  2 = Disagree  3 = Neither agree nor disagree  4 = Agree  5 = Strongly agree | Patient | 119 |
|  |  | | During your last visit/meeting, were you told about other methods of FP that you could use (methods other than the one you received)? | | No  Yes | Patient | 122 |
|  |  | | At that time, were you told about side effects or problems you might have with the method? | | No  Yes | Patient | 122 |
|  |  | | Were you told what to do if you experienced side effects or problems? | | No  Yes | Patient | 122 |
|  |  | | For the method you have just accepted, were you told how to use your method? | | No  Yes | Patient | 122 |
|  |  | | Did the FWA [family welfare assistant] talk about warning signs associated with the method you selected? | | No  Yes | Patient | 122 |
|  |  | | I was explained the benefits of taking my medicines | | **5-point Likert scale**  Strongly disagree  Strongly agree | Patient | 126 |
|  |  | | I was explained the risks of taking my medicines | | **5-point Likert scale**  Strongly disagree  Strongly agree | Patient | 126 |
|  |  | | I was explained the alternative treatments | | **5-point Likert scale**  Strongly disagree  Strongly agree | Patient | 126 |
|  |  | | I was explained what to expect if I do nothing | | **5-point Likert scale**  Strongly disagree  Strongly agree | Patient | 126 |
|  |  | | Pharmacist emphasized the important points | | **4-point Likert scale**  1 = Strongly disagree or Very poor  2 = Disagree or Poor  3 = Agree or Good  4 = Strongly agree or Excellent | Patient | 128 |
|  |  | | The procedure was explained to me | | **5-point Likert scale**  1 = Strongly disagree  2 = Disagree  3 = Neutral  4 = Agree  5 = Strongly agree | Patient | 129 |
|  |  | | Explanations the care provider gave you about your problem or condition | | **5-point Likert scale** | Patient | 17 |
|  |  | | Information the care provider gave you about medications (if any) | | **5-point Likert scale** | Patient | 17 |
|  |  | | Physician informs patients of medication side effects | | Missing | Patient | 131 |
|  |  | | Physician discusses how to live a healthy lifestyle | | Missing | Patient | 131 |
|  |  | | The doctor was good about explaining the reason for medical tests | | Missing | Patient | 132 |
|  |  | | staff ’s explanation of care | | **5-point Likert scale**  Very dissatisfied/unwilling  Very satisfied/willing | Patient | 134 |
|  |  | | Discuss pros and cons of each choice with you | | 1-100  (Higher = Better) | Patient | 136 |
|  |  | | Explain treatment alternatives | | 1-100  (Higher = Better) | Patient | 137 |
|  |  | | Explain side‐effects of medications | | 1-100  (Higher = Better) | Patient | 137 |
|  |  | | Tell you what to expect from your disease or treatment | | 1-100  (Higher = Better) | Patient | 137 |
|  |  | | Explanation of what was done for you | | **5-point Likert scale** | Patient | 138 |
|  |  | | The doctor gave me a poor explanation of my child's illness/health | | **5-point Likert scale** | Caregiver | 139 |
|  |  | | Physician told you everything | | **5-point Likert scale** | Observer | 140 |
|  |  | | Physician let you know test results | | **5-point Likert scale** | Observer | 140 |
|  |  | | Physician explained treatment alternatives | | **5-point Likert scale** | Observer | 140 |
|  |  | | Physician explained side effects of medications | | **5-point Likert scale** | Observer | 140 |
|  |  | | Physician told you what to expect | | **5-point Likert scale** | Observer | 140 |
|  |  | | Physician discussed the pros and cons | | **5-point Likert scale** | Observer | 142 |
|  | **Being transparent** | | Telling you everything; being truthful, upfront and frank; not keeping things from you that you should know | | **5-point Likert scale**  Poor  Excellent | patient | 18 |
|  |  | | If a physical examination was required for your health concerns, the doctor fully explained what was done and why | | **5-point Likert scale**  Strongly disagree  Strongly agree | Patient and Clinician | 47 |
|  |  | | Letting you know what he/she finds after examining you; not keeping you in the dark or confusing you | | Can’t say  Poor  Fair  Good  Very good  Excellent | Patient | 53 |
|  |  | | Indicating the likely cost of the treatment at the outset; never waiting until you are presented with the bill | | Can’t say  Poor  Fair  Good  Very good  Excellent | Patient | 53 |
|  |  | | Forewarning you of any likely pain involved and offering ways of reducing your pain | | Can’t say  Poor  Fair  Good  Very good  Excellent | Patient | 53 |
|  |  | | Were caregivers honest and clear about what to expect from the fertility care service? | | No, none at all  Some  Much  Yes, absolutely | Patient | 58 |
|  |  | | Was staff willing to talk to you about errors or incidents? | | Does not apply; nothing went wrong  No  Yes | Patient | 58 |
|  |  | | Information exchange: I felt the doctor was being honest with me | | **4-point Likert scale**  Does not apply  Strongly disagree  Disagree  Agree  Strongly agree | Patient | 62 |
|  |  | | Interpersonal skills: The doctor did not give me all the information I thought I should have been given | | **4-point Likert scale**  Does not apply  Strongly disagree  Disagree  Agree  Strongly agree | Patient | 62 |
|  |  | | Talking to you about how long you might have to live | | **11-point Likert scale**  The very worst  The very best | Patient | 15 |
|  |  | | The doctor spoke honestly about my illness and its treatment | | **5-point Likert scale**  I disagree completely  I agree completely | Patient | 113 |
|  |  | | Your physician informed you openly and directly of things concerning your illness that could be stressful (eg side effects of a treatment) | | **6-point Likert scale**  1 = Strongly disagree  6 = Strongly agree | Patient and Clinician | 118 |
|  |  | | Tell you everything | | 1-100  (Higher = Better) | Patient | 137 |
|  | **Addressing** | | Patient communication categories - Additional responses: Response to request for information; Agree; Register | | Missing | Observer | 22 |
|  |  | | Physician answered all questions | | **5-point Likert scale**  Strongly disagree  Strongly agree | Patient | 13 |
|  |  | | The psychiatrist answers all of my questions | | **5-point Likert scale**  Strongly disagree  Strongly agree | Patient | 9 |
|  |  | | Answering all your questions about your illness and treatment | | **11-point Likert scale**  The very worst  The very best | Patient | 15 |
|  |  | | Had the dermatologist answered all your questions and doubts? | | **5-point Likert scale**  1 = Not at all  5 = Yes, fully | Patient | 74 |
|  |  | | The dentist answers a question from the patient | | **5-point Likert scale**  Strongly disagree  Strongly agree | Patient and Clinician | 78 |
|  |  | | The nurse answers to the patient's questions | | 0 = Not done  1 = Done incorrectly  2 = Done correctly | Observer | 85 |
|  |  | | Prompt responses to questions about my drug therapy | | **7-point Likert scale**  Very dissatisfied  Very satisfied | Patient | 87 |
|  |  | | How well did this doctor answer your questions about your loved one's illness and treatment? | | **11-point Likert scale**  Very worst  Very best | Proxy/Caregiver | 90 |
|  |  | | The doctor was willing to answer my questions | | **4-point Likert scale**  Not at all  Very much so | Patient | 91 |
|  |  | | My doctor answered all my questions | | Never  Occasionally  Sometimes  Often  Always | Patient | 92 |
|  |  | | Resident addressed questions or concerns to your satisfaction | | **5-point Likert scale**  Strongly disagree  Strongly agree | Patient and Clinician | 93 |
|  |  | | Answering your questions clearly | | **5-point Likert scale**  Poor  Fair  Good  Very good  Excellent | Patient | 96 |
|  |  | | Dermatologist's answers to patient's questions | | **5-point Likert scale**  Poor  Fair  Good  Very good  Excellent | Patient | 98 |
|  |  | | She/he responded to my questions | | **10-point Likert scale**  Completely disagree  Completely agree | Patient | 108 |
|  |  | | How often does your family doctor clarify your questions regarding your treatment? | | **5-point Likert scale**  Not satisfied  Completely satisfied | Patient | 111 |
|  |  | | The doctors’ willingness to answer your questions | | Very poor  Best | Patient | 114 |
|  |  | | Did the FWA answer your questions clearly? | | No  Yes | Patient | 123 |
|  |  | | The staff answered my questions | | **5-point Likert scale**  1 = Strongly disagree  2 = Disagree  3 = Neutral  4 = Agree  5 = Strongly agree | Patient | 130 |
|  |  | | Answering your questions | | **5-point Likert scale**  Very dissatisfied/unwilling  Very satisfied/willing | Patient | 134 |
| **Gathering/having needed information** | |  |  | |  |  |  |
|  | **Exploring** | | The doctor/nurse gave me a thorough interview | | **5-point Likert scale**  Strongly disagree  Strongly agree | Patient | 37 |
|  |  | | When prescribing a new medication, my doctors asks if I have any questions about the medication(s) and possible side effects | | **6-point Likert scale**  None  All of the time | Patient | 40 |
|  |  | | I ask my HCP to explain the treatment or procedure in greater detail | | 1 = All the time  5 = Never | Patient | 49 |
|  |  | | I ask my HCP for recommendations about my medical symptoms | | 1 = All the time  5 = Never | Patient | 49 |
|  |  | | I ask my HCP a lot of questions about my medical symptoms | | 1 = All the time  5 = Never | Patient | 49 |
|  |  | | I ask questions regardless of my HCP’s reaction to them | | 1 = All the time  5 = Never | Patient | 49 |
|  |  | | The clinician explores the patient’s concerns (fears) about how problem(s) are to be managed | | 0 = The behaviour is not observed  4 = The behaviour is exhibited to a very high standard | Observer | 8 |
|  |  | | Asked for my ideas when we made a treatment plan | | **5-point Likert scale**  Almost never  Generally not  Sometimes  Most of the time  Almost always | Patient | 14 |
|  |  | | Asked to talk about any problems with my medicines or their effects | | **5-point Likert scale**  Almost never  Generally not  Sometimes  Most of the time  Almost always | Patient | 14 |
|  |  | | I asked my doctor to explain the treatment or condition in greater detail | | **5-point Likert scale**  Very strong disagreement with the statement  Very strong agreement with the statement | Patient | 11 |
|  |  | | I asked the doctor to give me his or her recommendations | | **5-point Likert scale**  Very strong disagreement with the statement  Very strong agreement with the statement | Patient | 11 |
|  |  | | Did you ask questions about things you want to know and things you don't understand about your treatment? | | No  Somewhat  Yes  Yes definitely | Patient | 70 |
|  |  | | For patients, occurrence of: Patient question asking | | Coding scheme of utterances | Observer | 71 |
|  |  | | If I do not understand something about my medication therapy, I ask my HCP to address my concerns | | 1 = Never  5 = Always  (Or “Not applicable”) | Patient | 75 |
|  |  | | The health care provider has asked me if I have questions and concerns about the procedure | | **4-point Likert scale**  1 = Strongly disagree  4 = Strongly agree | Patient | 76 |
|  |  | | Discuss patient’s role: What role should the patient play in making the decision? Patients should be offered a role in decision making, even if some will decline, preferring to defer to the physician | | Absence  Presence | Observer | 102 |
|  |  | | Explore patient’s preferences: Based on the information given, which way is the patient leaning | | Absence  Presence | Observer | 102 |
|  |  | | Patient asked questions | | **7-point Likert scale**  1 = Strongly disagree  7 = Strongly agree | Observer | 125 |
|  | **Understanding** | | Understood what I had to say | | **5-point Likert scale**  Strongly disagree  Strongly agree | Patient and Clinician | 47 |
|  |  | | This patient understood my explanations of the medical problem and treatment | | **5-point Likert scale**  Strongly disagree  Strongly agree | Clinician | 29 |
|  |  | | Understood what you had to say | | **5-point Likert scale**  1 = Strongly disagree  2 = Disagree  3 = Neither agree nor disagree  4 = Agree  5 = Strongly agree | Patient | 119 |
|  | **Checking** | | Practitioner uses summaries to bring together what the patient says about the topic | | **5-point Likert scale**  0 = Not at all  1 = Minimally  2 = To some extent  3 = A good deal  4 = A great extent | Observer | 31 |
|  |  | | Checked to be sure I understood everything | | Poor  Fair  Good  Very good  Excellent | Proxy/Caregiver | 6 |
|  |  | | Making sure the patient understood my explanations | | **7-point Likert scale**  Strongly disagree  Strongly agree | Clinician | 46 |
|  |  | | Making sure the patient understood my directions | | **7-point Likert scale**  Strongly disagree  Strongly agree | Clinician | 46 |
|  |  | | Checking my understanding of information the patient provided | | **7-point Likert scale**  Strongly disagree  Strongly agree | Clinician | 46 |
|  |  | | Checked to be sure I understood everything | | **5-point Likert scale**  Strongly disagree  Strongly agree | Patient and Clinician | 47 |
|  |  | | My HCP asks me whether I agree with his/her decisions | | 1 = All the time  5 = Never | Patient | 49 |
|  |  | | The clinician checks that the patient has understood the information | | 0 = The behaviour is not observed  4 = The behaviour is exhibited to a very high standard | Observer | 8 |
|  |  | | Checking what information the patient already knows | | **4-point Likert scale**  Not at all  Yes | Observer | 16 |
|  |  | | Checking whether the patient still wants to start CT after being educated | | **4-point Likert scale**  Not at all  Yes | Observer | 16 |
|  |  | | Checking the patients’ knowledge about chemotherapy | | **4-point Likert scale**  Not at all  Yes | Observer | 16 |
|  |  | | Checking the patients’ understanding of information | | **4-point Likert scale**  Not at all  Not really  On the whole, yes  Yes | Observer | 16 |
|  |  | | HCP questions me to be sure I understand how to properly use my medication(s) before I leave the pharmacy | | 1 = Never  5 = Always  (Or “Not applicable”) | Patient | 75 |
|  |  | | The dentist confirms patient understanding | | **5-point Likert scale**  Strongly disagree  Strongly agree | Patient and Clinician | 78 |
|  |  | | My doctor asked me whether I understood what he/she had told me about my condition or treatment | | Never  Occasionally  Sometimes  Often  Always | Patient | 92 |
|  |  | | Assess understanding: Is the patient now an “informed participant” with a working understanding of the decision? | | Absence  Presence | Observer | 102 |
|  |  | | The pharmacist made sure that I understood how to take my medicines | | Strongly disagree  Strongly agree | Patient | 109 |
|  |  | | My doctor checked to ensure that I understood everything | | **5-point Likert scale**  Strongly disagree  Strongly agree | Patient | 110 |
|  |  | | The doctor made sure I understood everything | | **5-point Likert scale**  I disagree completely  I agree completely | Patient | 113 |
|  |  | | Checked to be sure you understood | | **5-point Likert scale**  1 = Strongly disagree  2 = Disagree  3 = Neither agree nor disagree  4 = Agree  5 = Strongly agree | Patient | 119 |
|  |  | | Pharmacist made sure you understood | | **4-point Likert scale**  1 = Strongly disagree or Very poor  2 = Disagree or Poor  3 = Agree or Good  4 = Strongly agree or Excellent | Patient | 128 |
| **Making human connection** | |  |  | |  |  |  |
|  | **Connecting** | | I felt comfortable asking questions about my treatment and medications | | **5-point Likert scale**  Strongly disagree  Strongly agree | Patient | 45 |
| **Giving attention** | |  |  | |  |  |  |
|  | **Ignoring** | | My doctor ignores my opinion about treatment options | | **6-point Likert scale**  None  All of the time | Patient | 40 |
| **Self-efficacy building** | |  |  | |  |  |  |
|  | **Reassuring** | | Made me feel my eye condition can be correctly treated | | **5-point Likert scale**  Strongly disagree  Strongly agree | Patient | 112 |
|  |  | | The clinician reassures the patient or re-affirms that the clinician will support the patient to become informed or deliberate about the options. If the patient states that they have sought or obtained information prior to the encounter, the clinician supports such a deliberation process | | 0 = No effort  1 = Minimal effort  2 = Moderate effort  3 = Skilled effort  4 = Exemplary effort | Observer | 116 |
|  | **Supporting** | | Helping you to take control… (exploring with you what you can do to improve your health yourself; encouraging rather than "lecturing" you) | | **5-point Likert scale**  Poor  Excellent | Patient | 4 |
|  |  | | Physician helped me understand results | | **5-point Likert scale**  Strongly disagree  Strongly agree | Patient | 13 |
|  |  | | My doctor helped me understand all the information | | **5-point Likert scale**  Completely disagree  Completely agree | Patient | 1 |
|  |  | | Doctor's advice and help in making decisions about your care | | Missing | Patient | 133 |
| **OTHER** | |  |  | |  |  |  |
|  |  | | Did doctors make you feel that following your treatment plan would make a difference in your health? | | **5-point Likert scale** | Patient | 7 |
|  |  | | Did the doctors make you feel that your everyday activities such as your diet and lifestyle would make a difference in your health? | | **5-point Likert scale** | Patient | 7 |
|  |  | | I suggest a certain kind of medical treatment to HCP | | 1 = All the time  5 = Never | Patient | 49 |
|  |  | | I insist on a particular kind of test or treatment for my symptoms | | 1 = All the time  5 = Never | Patient | 49 |
|  |  | | It’s up to me to bring up the topic of pain during my medical appointment | | 1 = All the time  5 = Never | Patient | 49 |
|  |  | | Technical skills (thoroughness, carefulness, competence) of the physician you saw | | **5-point Likert scale**  Poor  Excellent | Patient | 28 |
|  |  | | The health workers were well coordinated | | **10-point Likert scale**  Completely disagree  Completely agree | Patient | 108 |

**F6 Dimension 6: Patient-clinician collaboration (manner)**

|  |  | | **Item** | | **Responses** | **Respondent** | **Instrument ID#** |
| --- | --- | --- | --- | --- | --- | --- | --- |
| **Facilitating patient involvement** | | |  |  |  |  |  |
|  | **Involving** | | Patient communication categories - Active involvement: 1a Asking questions; 1b Concern; 1c Assertive responses; 1d Positive affect | | Missing | Observer | 22 |
|  | **Encouraging** | | My doctor discourages my questions | | **6-point Likert scale**  None  All of the time | Patient | 40 |
|  |  | | My doctor strongly encourages me to express all of my concerns about the prescribed treatment | | **6-point Likert scale**  None  All of the time | Patient | 40 |
|  |  | | My doctor encourages me to ask questions; answers them clearly; never avoiding my question or lecturing me | | **4-point Likert scale**  Poor  Excellent | Patient | 54 |
|  |  | | The nurse encourages the patient to continue talking | | 0 = Not done  1 = Done incorrectly  2 = Done correctly | Observer | 85 |
|  |  | | The physician encourages me | | **4-point Likert scale**  Not at all  Very much | Patient and Clinician | 105 |
|  |  | | The doctor gave me hope | | **5-point Likert scale**  I disagree completely  I agree completely | Patient | 113 |
|  | **Allowing** | | Let me talk without interruptions | | Poor  Fair  Good  Very good  Excellent | Proxy/Caregiver | 6 |
|  |  | | My doctor makes it difficult for me to communicate my concerns about treatment decisions | | **6-point Likert scale**  None  All of the time | Patient | 40 |
|  |  | | My doctor lets me tell my story; listening carefully; asking thoughtful questions; and never interrupting me while I am talking | | **4-point Likert scale**  Poor  Excellent | Patient | 54 |
|  |  | | Being open | | **4-point Likert scale**  Not at all  Not really  On the whole, yes  Yes | Observer | 16 |
|  |  | | It is an easy atmosphere in which to ask the dentist a question | | **5-point Likert scale**  Strongly disagree  Strongly agree | Patient and Clinician | 78 |
|  |  | | Showing openness | | **5-point Likert scale**  Not at all  To a great extent | Observer | 95 |
|  |  | | The doctor sometimes interrupted me | | **5-point Likert scale**  I disagree completely  I agree completely | Patient | 113 |
|  |  | | Doctor let patient choose language | | **7-point Likert scale**  1 = Strongly disagree  7 = Strongly agree | Observer | 125 |
|  |  | | Doctor welcomed non-medical talk | | **7-point Likert scale**  1 = Strongly disagree  7 = Strongly agree | Observer | 125 |
| **Adjusting to individual patient** | |  |  | |  |  |  |
|  | **Tailoring (general)** | | Practitioner demonstrates sensitivity to talking about other issues | | **5-point Likert scale**  0 = Not at all  1 = Minimally  2 = To some extent  3 = A good deal  4 = A great extent | Observer | 31 |
|  | **Tailoring language** | | Using words you can understand when explaining your problems and treatment; explaining any technical medical terms in plain language | | **5-point Likert scale**  Poor  Excellent | patient | 18 |
|  |  | | When you asked questions, did you get answers you could understand? | | No  Yes sometimes  Yes always | Patient or Caregiver | 30 |
|  |  | | When practitioner provides information it is sensitive to patient concerns and understanding | | **5-point Likert scale**  0 = Not at all  1 = Minimally  2 = To some extent  3 = A good deal  4 = A great extent | Observer | 31 |
|  |  | | Did this doctor explain things in a way that was easy to understand? | | No  Somewhat  Yes  Yes definitely | Patient | 3 |
|  |  | | Talked in terms I could understand | | Poor  Fair  Good  Very good  Excellent | Proxy/Caregiver | 6 |
|  |  | | My therapy program was explained to me in a way that I could understand | | **5-point Likert scale**  Definitely not true  Completely true | Patient | 32 |
|  |  | | During this hospital stay, how often did nurses: explain things in a way you could understand? | | **4-point Likert scale**  Never  Sometimes  Usually  Always | Patient | 12 |
|  |  | | During this hospital stay, how often did doctors: explain things in a way you could understand? | | **4-point Likert scale**  Never  Sometimes  Usually  Always | Patient | 12 |
|  |  | | Before giving you any new medications, how often did hospital staff describe possible side effects in a way you could understand? | | **4-point Likert scale**  Never  Sometimes  Usually  Always | Patient | 12 |
|  |  | | Restrictions relating to my illness were explained to me in a way that I could understand | | **5-point Likert scale**  Totally disagree  Totally agree | Patient | 44 |
|  |  | | I was addressed in clear and intelligible language | | **5-point Likert scale**  Totally disagree  Totally agree | Patient | 44 |
|  |  | | 2I was given guidance about pain treatment in a way that I could understand | | **5-point Likert scale**  Totally disagree  Totally agree | Patient | 44 |
|  |  | | Did the doctors use medical words you did not understand? | | **5-point Likert scale** | Patient | 7 |
|  |  | | Did you feel confused about what was going on with your medical care because doctors did not explain things well? | | **5-point Likert scale** | Patient | 7 |
|  |  | | The doctor did not use any words that I did not understand | | **5-point Likert scale**  Strongly disagree  Strongly agree | Patient | 48 |
|  |  | | Talking in plain language, using words you can understand; never being too technical or complicated | | Can’t say  Poor  Fair  Good  Very good  Excellent | Patient | 53 |
|  |  | | My doctor uses words I can understand when explaining my problems and treatment; explains any technical terms | | **4-point Likert scale**  Poor  Excellent | Patient | 54 |
|  |  | | How often did staff use difficult words without explaining them to you? | | Never  Sometimes  Usually  Always | Patient | 58 |
|  |  | | The AP used words I understood | | **5-point Likert scale**  Strongly disagree  Strongly agree | Patient | 59 |
|  |  | | Quality of time: The doctor used words I did not understand | | **4-point Likert scale**  Does not apply  Strongly disagree  Disagree  Agree  Strongly agree | Patient | 62 |
|  |  | | When you had important questions to ask a doctor, did you get answers that you could understand? | | 1 = Yes completely  2 = Yes to some extent  3 = No | Patient and Caregiver | 63 |
|  |  | | When you had important questions to ask a nurse, did you get answers that you could understand? | | 1 = Yes completely  2 = Yes to some extent  3 = No | Patient and Caregiver | 63 |
|  |  | | Did a member of staff explain the purpose of the medicines you were to take at home in a way you could understand? | | 1 = Yes completely  2 = Yes to some extent  3 = No | Patient and Caregiver | 63 |
|  |  | | The physician’s explanations were easy to understand | | **5-point Likert scale**  I do not agree  I fully agree | Patient | 66 |
|  |  | | Using words you can understand | | **11-point Likert scale**  The very worst  The very best | Patient | 15 |
|  |  | | I couldn't really understand what the physician was trying to explain | | Disagree  Agree | Patient | 73 |
|  |  | | The health care provider explained my procedure/care to me in a way that I easily understood | | **4-point Likert scale**  1 = Strongly disagree  4 = Strongly agree | Patient | 76 |
|  |  | | The dentist explains to the patient using plain words | | **5-point Likert scale**  Strongly disagree  Strongly agree | Patient and Clinician | 78 |
|  |  | | Was the language used by the doctor clear? | | No  Yes | Patient | 83 |
|  |  | | The information given to me is clear and understandable | | No  Yes | Patient | 84 |
|  |  | | The nurse communicates with the patient with simple and understandable words | | 0 = Not done  1 = Done incorrectly  2 = Done correctly | Observer | 85 |
|  |  | | Clarity of information about drug therapy provided by the medical staff | | **7-point Likert scale**  Very dissatisfied  Very satisfied | Patient | 87 |
|  |  | | The doctor explained my condition in language I could understand | | **4-point Likert scale**  Not at all  Very much so | Patient | 91 |
|  |  | | Information communicated by resident was easy to understand | | **5-point Likert scale**  Strongly disagree  Strongly agree | Patient and Clinician | 93 |
|  |  | | Using language you can understand when explaining your problems and treatments | | **5-point Likert scale**  Poor  Fair  Good  Very good  Excellent | Patient | 96 |
|  |  | | Did the doctor use words that were easy to understand? | | **9-point Likert scale**  Used very hard words  Used very easy words | Patient | 97 |
|  |  | | My HIV provider explains things in a way I can understand | | Never  Sometimes  Usually  Always | Patient | 100 |
|  |  | | She/he talked in a way that helped me understand my condition | | **10-point Likert scale**  Completely disagree  Completely agree | Patient | 108 |
|  |  | | My doctor uses too many technical terms that confuse me | | **5-point Likert scale**  Strongly disagree  Strongly agree | Patient | 110 |
|  |  | | Explained the treatment to you in a way that made you feel that you better understood your eye condition | | **5-point Likert scale**  Very dissatisfied  Very satisfied | Patient | 112 |
|  |  | | The doctor used medical terms without explaining their meaning | | **5-point Likert scale**  I disagree completely  I agree completely | Patient | 113 |
|  |  | | Was the information given to you by the FWA easy to understand? | | No  Yes | Patient | 123 |
|  |  | | Pharmacist used easy-to-understand language | | **4-point Likert scale**  1 = Strongly disagree or Very poor  2 = Disagree or Poor  3 = Agree or Good  4 = Strongly agree or Excellent | Patient | 128 |
|  |  | | Degree to which care provider talked with you using words you could understand | | **5-point Likert scale** | Patient | 17 |
| **Providing information** | |  |  | |  |  |  |
|  | **Informing** | | Psychosocial care | | **4-point Likert scale**  Not at all  Not really  On the whole, yes  Yes | Observer | 16 |
|  |  | | For patients, occurrence of: Assertive responses | | Coding scheme of utterances | Observer | 71 |
|  |  | | All of the options available to me are clearly explained | | No  Yes | Patient | 84 |
|  |  | | During this visit, how often did the physician explain things in a way that could be easily understood? | | Never  Sometimes  Always | Patient | 120 |
|  | **Being transparent** | | My doctor tells me everything; is truthful, up-front and frank; does not keep things from me | | **4-point Likert scale**  Poor  Excellent | Patient | 54 |
|  | **Addressing** | | If you had any anxieties or fears about your condition or treatment, did a doctor discuss them with you? | | 1 = Yes completely  2 = Yes to some extent  3 = No | Patient and Caregiver | 63 |
|  |  | | If you had any anxieties or fears about your condition or treatment, did a nurse discuss them with you? | | 1 = Yes completely  2 = Yes to some extent  3 = No | Patient and Caregiver | 63 |
|  |  | | Lessening fears and anxieties | | **5-point Likert scale**  Negative  Positive | Patient | 79 |
|  |  | | The doctor was willing to discuss my worries and fears | | **5-point Likert scale**  I disagree completely  I agree completely | Patient | 113 |
| **Gathering/having needed information** | |  |  | |  |  |  |
|  | **Exploring** | | Doctor communication categories - Closed questions | | Missing | Observer | 22 |
|  |  | | Doctor communication categories - Open questions (NB that do not fall within patient-centred category) | | Missing | Observer | 22 |
|  |  | | Talking with you about your feelings concerning the possibility that you might getting sicker | | **11-point Likert scale**  The very worst  The very best | Patient | 15 |
|  |  | | Exploring the patients’ feelings about treatment | | **4-point Likert scale**  Not at all  Yes | Observer | 16 |
|  | **Understanding** | | I felt this doctor really knew what I was thinking | | **5-point Likert scale**  Strongly disagree  Strongly agree | Patient | 35 |
|  |  | | I really felt understood by my doctor | | **7-point Likert scale**  1 = Very strongly disagree  2 = Strongly disagree  3 = Disagree  4 = Unsure  5 = Agree  6 = Strongly agree  7 = Very strongly agree | Patient | 10 |
|  |  | | I felt this doctor/nurse/health visitor really knew what I was thinking | | **5-point Likert scale**  Strongly disagree  Strongly agree | Patient | 51 |
|  |  | | My doctor understands me | | **5-point Likert scale**  Not at all appropriate  Totally appropriate | Patient | 26 |
|  |  | | The doctor understood what was on my mind | | Disagree completely  Disagree  So so  Agree  Agree completely | Patient | 61 |
|  |  | | Information exchange: I really felt understood by my doctor | | **4-point Likert scale**  Does not apply  Strongly disagree  Disagree  Agree  Strongly agree | Patient | 62 |
|  |  | | He/she understood my emotions | | **5-point Likert scale**  1 = Not at all  5 = Very much | Patient | 64 |
|  |  | | My psychiatrist understands what I tell him or her | | **5-point Likert scale**  Strongly disagree  Strongly agree | Patient | 9 |
|  |  | | I could NOT understand all that this patient wanted to tell me | | **5-point Likert scale**  Strongly disagree  Strongly agree | Clinician | 29 |
|  |  | | I really felt understood by my child's doctor | | **5-point Likert scale** | Caregiver | 139 |
| **Making human connection** | |  |  | |  |  |  |
|  | **Caring** | | Concern for patient as a person | | **5-point Likert scale**  1 = Poor  5 = Excellent | Patient | 5 |
|  |  | | How much did you feel the doctor cared about you? | | 0 = Never/Not at all  10 = Always/Completely | Proxy/Caregiver | 43 |
|  |  | | The doctor's concern for me as a person in this visit was | | **5-point Likert scale**  Poor  Excellent | Patient | 24 |
|  |  | | Showed care and concern about me as a person | | **5-point Likert scale**  Strongly disagree  Strongly agree | Patient and Clinician | 47 |
|  |  | | Provide a caring atmosphere rather than just give you information | | **7-point Likert scale**  Not at all perceived  Perceived to a very great extent | Proxy/Caregiver | 25 |
|  |  | | Look at the needs of your ‘whole’ child instead of just physical needs | | **7-point Likert scale**  Not at all perceived  Perceived to a very great extent | Proxy/Caregiver | 25 |
|  |  | | My doctor really seemed to care about my health problems and me | | **5-point Likert scale**  Lowest satisfaction  Highest satisfaction | Patient | 52 |
|  |  | | I felt I was taken care of | | Disagree completely  Disagree  So so  Agree  Agree completely | Patient | 61 |
|  |  | | The physician did all he/she could to put me at ease | | **5-point Likert scale**  I do not agree  I fully agree | Patient | 66 |
|  |  | | Caring about you as a person | | **11-point Likert scale**  The very worst  The very best | Patient | 15 |
|  |  | | Health care provider really cares about me as a person | | **4-point Likert scale**  1 = Strongly disagree  4 = Strongly agree | Patient | 76 |
|  |  | | To care | | **7-point Likert scale**  Strongly disagree  Strongly agree | Patient | 107 |
|  |  | | 2Overall, the health workers were sensitive | | **10-point Likert scale**  Completely disagree  Completely agree | Patient | 108 |
|  |  | | The doctor cares about my health as much as I do | | **5-point Likert scale**  Strongly disagree  Strongly agree | Patient | 110 |
|  |  | | Was caring | | **5-point Likert scale**  Strongly disagree  Strongly agree | Patient | 112 |
|  |  | | Seems concerned about me and my family | | **7-point Likert scale**  1 = Strongly disagree  7 = Strongly agree | Patient | 117 |
|  |  | | Showed concern about you as a person | | **5-point Likert scale**  1 = Strongly disagree  2 = Disagree  3 = Neither agree nor disagree  4 = Agree  5 = Strongly agree | Patient | 119 |
|  |  | | The doctor seemed to care about my child’s feelings | | **5-point Likert scale** | Caregiver | 139 |
|  | **Connecting** | | I felt comfortable expressing my feelings to program staff | | **5-point Likert scale**  Definitely not true  Completely true | Patient | 32 |
|  |  | | Program staff tried to ensure my comfort | | **5-point Likert scale**  Definitely not true  Completely true | Patient | 32 |
|  |  | | I felt able to tell this doctor about very personal things | | **5-point Likert scale**  Strongly disagree  Strongly agree | Patient | 35 |
|  |  | | I can easily talk about personal things with this doctor | | **5-point Likert scale**  Strongly disagree  Strongly agree | Patient | 21 |
|  |  | | Doctor communication categories - Patient-centred: 1a Partnership-building; 1b Supportive talk | | Missing | Observer | 22 |
|  |  | | I felt really understood by my doctor/nurse | | **5-point Likert scale**  Strongly disagree  Strongly agree | Patient | 37 |
|  |  | | I felt free to talk about my private thoughts | | **5-point Likert scale**  Strongly disagree  Strongly agree | Patient | 37 |
|  |  | | The comfort and support they gave you? | | **5-point Likert scale**  Poor  Excellent | Patient | 38 |
|  |  | | I was appreciated | | **5-point Likert scale**  Totally disagree  Totally agree | Patient | 44 |
|  |  | | I was accepted for what I was | | **5-point Likert scale**  Totally disagree  Totally agree | Patient | 44 |
|  |  | | I was able to discuss issues with the staff in confidence if necessary | | **5-point Likert scale**  Totally disagree  Totally agree | Patient | 44 |
|  |  | | I felt free to complain without fear of retaliation | | **5-point Likert scale**  Strongly disagree  Strongly agree | Patient | 45 |
|  |  | | I felt safe to refuse medication or treatment during my hospital stay | | **5-point Likert scale**  Strongly disagree  Strongly agree | Patient | 45 |
|  |  | | I felt free to talk to this doctor about private matters | | **7-point Likert scale**  1 = Very strongly disagree  2 = Strongly disagree  3 = Disagree  4 = Unsure  5 = Agree  6 = Strongly agree  7 = Very strongly agree | Patient | 10 |
|  |  | | I could freely talk to the doctor about my private issues | | **5-point Likert scale**  Strongly disagree  Strongly agree | Patient | 48 |
|  |  | | I felt able to tell this doctor/nurse/health visitor about very personal things | | **5-point Likert scale**  Strongly disagree  Strongly agree | Patient | 51 |
|  |  | | I could talk to the specialist easily and openly | | **5-point Likert scale**  1 = Strong disagreement  3 = Agreement  5 = Strong agreement | Patient | 55 |
|  |  | | I felt I could ask my specialist questions | | **5-point Likert scale**  1 = Strong disagreement  3 = Agreement  5 = Strong agreement | Patient | 55 |
|  |  | | I was able to develop a friendly relationship with my specialist | | **5-point Likert scale**  1 = Strong disagreement  3 = Agreement  5 = Strong agreement | Patient | 55 |
|  |  | | I felt comfortable discussing my problems with the provider | | **5-point Likert scale**  1 = Very little agreement  5 = Very much in agreement | Patient | 56 |
|  |  | | The doctor made me feel completely at ease in the consultation | | **6-point Likert scale**  Strongly disagree  Strongly agree | Patient or Observer | 57 |
|  |  | | I could talk to the AP | | **5-point Likert scale**  Strongly disagree  Strongly agree | Patient | 59 |
|  |  | | I can talk to my doctor | | **5-point Likert scale**  Not at all appropriate  Totally appropriate | Patient | 26 |
|  |  | | We had a good talk | | Disagree completely  Disagree  So so  Agree  Agree completely | Patient | 61 |
|  |  | | It was a bit difficult to connect with the doctor | | Disagree completely  Disagree  So so  Agree  Agree completely | Patient | 61 |
|  |  | | I felt like one of the crowd | | Disagree completely  Disagree  So so  Agree  Agree completely | Patient | 61 |
|  |  | | He/she inspired confidence and security when touching me and being nearby | | **5-point Likert scale**  1 = Not at all  5 = Very much | Patient | 64 |
|  |  | | The computer gets in the way of the psychiatrist | | **5-point Likert scale**  Strongly disagree  Strongly agree | Patient | 9 |
|  |  | | For clinician, occurrence of:Partnership building | | Coding scheme of utterances | Observer | 71 |
|  |  | | Have you felt comfortable discussing personal and sensitive issues with the dermatologist? | | **5-point Likert scale**  1 = Not at all  5 = Yes, fully | Patient | 74 |
|  |  | | When I receive prescriptions from my pharmacist, HCP is easily approachable to discuss my medication(s) | | 1 = Never  5 = Always  (Or “Not applicable”) | Patient | 75 |
|  |  | | This patient is very personable | | **5-point Likert scale**  Strongly disagree  Strongly agree | Clinician | 29 |
|  |  | | I established effective rapport with this patient | | **5-point Likert scale**  Strongly disagree  Strongly agree | Clinician | 29 |
|  |  | | If I am sitting, the person giving me information is too | | No  Yes | Patient | 84 |
|  |  | | The nurse builds up comfort for the patient during communication | | 0 = Not done  1 = Done incorrectly  2 = Done correctly | Observer | 85 |
|  |  | | The nurse uses relaxing words indicating affection during the conversation with the patient | | 0 = Not done  1 = Done incorrectly  2 = Done correctly | Observer | 85 |
|  |  | | The nurse speaks with a calm tone and measured words during the conversation with the patient | | 0 = Not done  1 = Done incorrectly  2 = Done correctly | Observer | 85 |
|  |  | | The nurse uses humor in communicating with patients | | 0 = Not done  1 = Done incorrectly  2 = Done correctly | Observer | 85 |
|  |  | | I felt the doctor disapproved of me | | **4-point Likert scale**  Not at all  Very much so | Patient | 91 |
|  |  | | The doctor was a warm and friendly person | | **4-point Likert scale**  Not at all  Very much so | Patient | 91 |
|  |  | | The doctor was someone I could trust | | **4-point Likert scale**  Not at all  Very much so | Patient | 91 |
|  |  | | My doctor made me feel important today | | Never  Occasionally  Sometimes  Often  Always | Patient | 92 |
|  |  | | I can speak freely with the physician | | **4-point Likert scale**  Not at all  Very much | Patient and Clinician | 105 |
|  |  | | The health worker made a good impression on me | | **10-point Likert scale**  Completely disagree  Completely agree | Patient | 108 |
|  |  | | The doctor made me feel I could ask or say anything | | **5-point Likert scale**  I disagree completely  I agree completely | Patient | 113 |
|  |  | | The doctor made me feel important | | **5-point Likert scale**  I disagree completely  I agree completely | Patient | 113 |
|  |  | | The doctor kept me at a distance | | **5-point Likert scale**  I disagree completely  I agree completely | Patient | 113 |
|  |  | | Doctor connected to patient | | **7-point Likert scale**  1 = Strongly disagree  7 = Strongly agree | Observer | 125 |
|  | **Sympathizing** | | Showing care and compassion (seeming genuinely concerned, connecting with you on a human level; not being indifferent or "detached") | | **5-point Likert scale**  Poor  Excellent | Patient | 4 |
|  |  | | Showed care and concern | | Poor  Fair  Good  Very good  Excellent | Proxy/Caregiver | 6 |
|  |  | | Was sympathetic | | **7-point Likert scale**  Very strongly disagree  Very strongly agree | Patient | 19 |
|  |  | | Treating you with care and concern | | **5-point Likert scale**  Very poor  Very good | Patient | 41 |
|  |  | | Treating you with care and concern | | Doesn’t apply  Very poor  Poor  Neither good nor poor  Good  Very good | Patient | 50 |
|  |  | | Being sensitive, understanding and patient with you; never rough, unsympathetic or impatient | | Can’t say  Poor  Fair  Good  Very good  Excellent | Patient | 53 |
|  |  | | Health care providers show compassion and support for patients | | **6-point Likert scale**  Poor  Very good | Patients | 60 |
|  |  | | He/she was able to put him/herself in “my shoes” | | **5-point Likert scale**  1 = Not at all  5 = Very much | Patient | 64 |
|  |  | | Showing empathy | | **4-point Likert scale**  Not at all  Not really  On the whole, yes  Yes | Observer | 16 |
|  |  | | The person giving me the news seems to be sympathetic | | No  Yes | Patient | 84 |
|  |  | | Treating you with care and concern | | **5-point Likert scale**  Extremely bad  Extremely good | Patient | 86 |
|  |  | | The nursing staff showed utmost overall courtesy, concern and care | | **5-point Likert scale**  Strongly disagree  Strongly agree | Patient | 89 |
|  |  | | Hope | | **7-point Likert scale**  Strongly disagree  Strongly agree | Patient | 107 |
|  |  | | Sympathy | | **7-point Likert scale**  Strongly disagree  Strongly agree | Patient | 107 |
|  |  | | She/he was considerate | | **10-point Likert scale**  Completely disagree  Completely agree | Patient | 108 |
|  |  | | Can view things from my perspective (see things as I see them) | | **7-point Likert scale**  1 = Strongly disagree  7 = Strongly agree | Patient | 117 |
|  | **Respecting** | | Treating you like you’re on the same level; never “talking down” to you or treating you like a child | | **5-point Likert scale**  Poor  Excellent | patient | 18 |
|  |  | | Practitioner actively conveys respect for patient choice about behavior change | | **5-point Likert scale**  0 = Not at all  1 = Minimally  2 = To some extent  3 = A good deal  4 = A great extent | Observer | 31 |
|  |  | | Did doctor show respect for what you had to say? | | No  Somewhat  Yes  Yes definitely | Patient | 3 |
|  |  | | Treated me with respect | | Poor  Fair  Good  Very good  Excellent | Proxy/Caregiver | 6 |
|  |  | | The program staff treated me as a person instead of just another case | | **5-point Likert scale**  Definitely not true  Completely true | Patient | 32 |
|  |  | | Was the RTT respectful towards you? | | **5-point Likert scale**  Not at all  Very much | Patient | 34 |
|  |  | | Respect shown to patient | | **5-point Likert scale**  1 = Poor  5 = Excellent | Patient | 5 |
|  |  | | During this hospital stay, how often did nurses: treat you with respect and courtesy? | | **4-point Likert scale**  Never  Sometimes  Usually  Always | Patient | 12 |
|  |  | | During this hospital stay, how often did doctors: treat you with respect and courtesy? | | **4-point Likert scale**  Never  Sometimes  Usually  Always | Patient | 12 |
|  |  | | My provider(s) treats me with dignity and respect | | **4-point Likert scale**  Strongly disagree  Strongly agree | Patient | 42 |
|  |  | | My provider(s) treats me as a whole person | | **4-point Likert scale**  Strongly disagree  Strongly agree | Patient | 42 |
|  |  | | I was treated with respect | | **5-point Likert scale**  Totally disagree  Totally agree | Patient | 44 |
|  |  | | I was treated with dignity and respect | | **5-point Likert scale**  Strongly disagree  Strongly agree | Patient | 45 |
|  |  | | The respect shown to me by this doctor was | | **5-point Likert scale**  Poor  Excellent | Patient | 24 |
|  |  | | The practice’s respect of your right to seek a second opinion was | | **5-point Likert scale**  Poor  Excellent | Patient | 24 |
|  |  | | Treat you as an equal rather than just as the parent of a patient | | **7-point Likert scale**  Not at all perceived  Perceived to a very great extent | Proxy/Caregiver | 25 |
|  |  | | Treat you as an individual rather than as a 'typical' parent of a child with a disability | | **7-point Likert scale**  Not at all perceived  Perceived to a very great extent | Proxy/Caregiver | 25 |
|  |  | | My doctor treats me like I am on the same level, never talking down to me or treating me like a child | | **4-point Likert scale**  Poor  Excellent | Patient | 54 |
|  |  | | The doctor did not treat me as an equal in the consultation | | **6-point Likert scale**  Strongly disagree  Strongly agree | Patient or Observer | 57 |
|  |  | | How often did the physician take you seriously? | | Never  Sometimes  Usually  Always | Patient | 58 |
|  |  | | Health care providers are respectful to patients | | **6-point Likert scale**  Poor  Very good | Patients | 60 |
|  |  | | Interpersonal skills: The doctor did not take my problems very seriously | | **4-point Likert scale**  Does not apply  Strongly disagree  Disagree  Agree  Strongly agree | Patient | 62 |
|  |  | | Overall, did you feel you were treated with respect and dignity while you were in hospital? | | 1 = Yes completely  2 = Yes to some extent  3 = No | Patient and Caregiver | 63 |
|  |  | | Not always treated with respect and dignity | | 1 = Yes completely  2 = Yes to some extent  3 = No | Patient and Caregiver | 63 |
|  |  | | He/she respected me as a person | | **5-point Likert scale**  1 = Not at all  5 = Very much | Patient | 64 |
|  |  | | The physician respects the fact that I may have a different opinion regarding treatment | | **5-point Likert scale**  I do not agree  I fully agree | Patient | 66 |
|  |  | | During the visit, have you been treated with regard and consideration? | | **5-point Likert scale**  1 = Not at all  5 = Yes, fully | Patient | 74 |
|  |  | | Did you feel respected by the dermatologists during the visit? | | **5-point Likert scale**  1 = Not at all  5 = Yes, fully | Patient | 74 |
|  |  | | The health care provider didn’t show respect to what I have to say | | **4-point Likert scale**  1 = Strongly disagree  4 = Strongly agree | Patient | 76 |
|  |  | | The dentist respects patient privacy | | **5-point Likert scale**  Strongly disagree  Strongly agree | Patient and Clinician | 78 |
|  |  | | Respect shown to the patient | | **5-point Likert scale**  Negative  Positive | Patient | 79 |
|  |  | | Respect of privacy | | **5-point Likert scale**  Negative  Positive | Patient | 79 |
|  |  | | The nurse respects the privacy of the patient | | 0 = Not done  1 = Done incorrectly  2 = Done correctly | Observer | 85 |
|  |  | | The doctor gave you appropriate courtesy and respect | | **5-point Likert scale**  Strongly disagree  Strongly agree | Patient | 89 |
|  |  | | The doctor treated me with respect | | **4-point Likert scale**  Not at all  Very much so | Patient | 91 |
|  |  | | Treating you like you’re on the same level; not “talking down” to you | | **5-point Likert scale**  Poor  Fair  Good  Very good  Excellent | Patient | 96 |
|  |  | | How courteous and respectful was the doctor? | | **9-point Likert scale**  Not at all courteous  Very courteous | Patient | 97 |
|  |  | | Does your HIV provider treat you with (a great deal) of respect and dignity? | | None at all  Not too much  A fair amount  A great deal | Patient | 100 |
|  |  | | The doctor treated me with respect and dignity | | **5-point Likert scale**  Strongly disagree  Strongly agree | Patient | 101 |
|  |  | | Respectful | | **7-point Likert scale**  Strongly disagree  Strongly agree | Patient | 107 |
|  |  | | Overall, the health workers had respect | | **10-point Likert scale**  Completely disagree  Completely agree | Patient | 108 |
|  |  | | The doctor made me feel like a statistic, rather than a person | | **5-point Likert scale**  I disagree completely  I agree completely | Patient | 113 |
|  |  | | The doctor respected my need for privacy and dignity | | **5-point Likert scale**  I disagree completely  I agree completely | Patient | 113 |
|  |  | | Courtesy, politeness, and respect shown by the doctor | | Very poor  Best | Patient | 114 |
|  |  | | During this visit, how often did the physician treat you with courtesy and respect? | | Never  Sometimes  Always | Patient | 120 |
|  |  | | The doctor should have given me more respect | | Missing | Patient | 132 |
|  |  | | Respectfulness (tone of voice and attitudes expressed toward the patient) | | 1-5  (Higher = Better) | Observer | 135 |
|  | **Being courteous** | | Greeting you warmly; calling you by the name you prefer; being friendly, never crabby or rude | | **5-point Likert scale**  Poor  Excellent | patient | 18 |
|  |  | | How would you rate the courtesy of your/your child's healthcare provider? | | Poor  Fair  Good  Very good  Excellent | Patient or Caregiver | 30 |
|  |  | | Making you feel at ease… (being friendly and warm towards you, treating you with respect; not cold or abrupt) | | **5-point Likert scale**  Poor  Excellent | Patient | 4 |
|  |  | | Greeted me in a way that made me feel comfortable | | Poor  Fair  Good  Very good  Excellent | Proxy/Caregiver | 6 |
|  |  | | Warmth of provider's greeting | | **5-point Likert scale**  1 = Poor  5 = Excellent | Patient | 5 |
|  |  | | I was treated in a friendly way | | **5-point Likert scale**  Totally disagree  Totally agree | Patient | 44 |
|  |  | | The warmth of the doctor’s greeting to me was | | **5-point Likert scale**  Poor  Excellent | Patient | 24 |
|  |  | | Greeted me in a way that made me feel comfortable | | **5-point Likert scale**  Strongly disagree  Strongly agree | Patient and Clinician | 47 |
|  |  | | The doctor seemed warm and friendly to me | | **7-point Likert scale**  1 = Very strongly disagree  2 = Strongly disagree  3 = Disagree  4 = Unsure  5 = Agree  6 = Strongly agree  7 = Very strongly agree | Patient | 10 |
|  |  | | The doctor spoke politely to me | | **5-point Likert scale**  Strongly disagree  Strongly agree | Patient | 48 |
|  |  | | The doctor greeted me before addressing my complaints | | **5-point Likert scale**  Strongly disagree  Strongly agree | Patient | 48 |
|  |  | | The doctor was not friendly to me | | **5-point Likert scale**  Strongly disagree  Strongly agree | Patient | 48 |
|  |  | | How much did your doctor act warm and open to you, for example by smiling or looking you in the eye? | | **5-point Likert scale**  Lowest satisfaction  Highest satisfaction | Patient | 52 |
|  |  | | My doctor acted as though he/she were doing me a favor by talking to me | | **5-point Likert scale**  Lowest satisfaction  Highest satisfaction | Patient | 52 |
|  |  | | Greeting you in a friendly way; not being grumpy or rude to you | | Can’t say  Poor  Fair  Good  Very good  Excellent | Patient | 53 |
|  |  | | Treating you with courtesy, respect and as an equal; never belittling you or making you feel stupid | | Can’t say  Poor  Fair  Good  Very good  Excellent | Patient | 53 |
|  |  | | My doctor greets me warmly, calls me by the name I prefer and is never crabby or rude | | **4-point Likert scale**  Poor  Excellent | Patient | 54 |
|  |  | | How much did your provider act warm and open to you, for example, by smiling or looking you in the eye? | | **5-point Likert scale**  1 = Very little agreement  5 = Very much in agreement | Patient | 56 |
|  |  | | How often did you have the impression that staff was talking “about” you instead of talking to you? | | Never  Sometimes  Usually  Always | Patient | 58 |
|  |  | | Health care providers are welcoming during consultations | | **6-point Likert scale**  Poor  Very good | Patients | 60 |
|  |  | | My doctor treated me in a friendly and courteous manner | | **5-point Likert scale**  1 = Strongly agree  2 = Agree  3 = Uncertain  4 = Disagree  5 = Strongly disagree | Patient | 27 |
|  |  | | Interpersonal skills: The doctor was not friendly to me | | **4-point Likert scale**  Does not apply  Strongly disagree  Disagree  Agree  Strongly agree | Patient | 62 |
|  |  | | Quality of time: The doctor went straight to my medical problem without first greeting me | | **4-point Likert scale**  Does not apply  Strongly disagree  Disagree  Agree  Strongly agree | Patient | 62 |
|  |  | | Did doctors talk in front of you as if you weren't there? | | 1 = Yes completely  2 = Yes to some extent  3 = No | Patient and Caregiver | 63 |
|  |  | | Doctors sometimes talked as if I wasn't there | | 1 = Yes completely  2 = Yes to some extent  3 = No | Patient and Caregiver | 63 |
|  |  | | He/she turned to me in a calm and quiet tone | | **5-point Likert scale**  1 = Not at all  5 = Very much | Patient | 64 |
|  |  | | Psychiatrists act too businesslike and impersonal toward me | | **5-point Likert scale**  Strongly disagree  Strongly agree | Patient | 9 |
|  |  | | My psychiatrist treats me in a very friendly and courteous manner | | **5-point Likert scale**  Strongly disagree  Strongly agree | Patient | 9 |
|  |  | | Looking you in eye | | **11-point Likert scale**  The very worst  The very best | Patient | 15 |
|  |  | | Being kind | | **4-point Likert scale**  Not at all  Not really  On the whole, yes  Yes | Observer | 16 |
|  |  | | The personal manner (courtesy, respect, sensitivity, friendliness) of the person you saw | | **5-point Likert scale**  Poor  Excellent | Patient | 28 |
|  |  | | The physician was polite | | Disagree  Agree | Patient | 73 |
|  |  | | The physician hardly looked at me when we talked | | Disagree  Agree | Patient | 73 |
|  |  | | The health care provider treated me in a very friendly and courteous manner | | **4-point Likert scale**  1 = Strongly disagree  4 = Strongly agree | Patient | 76 |
|  |  | | Manner of receiving patients | | **5-point Likert scale**  Negative  Positive | Patient | 79 |
|  |  | | How did you consider the physician’s interpersonal manner? | | Very bad  Bad  Neither good nor bad  Good  Very good | Patient | 80 |
|  |  | | How did you consider the nurse’s interpersonal manner? | | Very bad  Bad  Neither good nor bad  Good  Very good | Patient | 80 |
|  |  | | If I am sitting, everyone in the room is also sitting | | No  Yes | Patient | 84 |
|  |  | | The nurse says hello to the patient at the beginning of the communication | | 0 = Not done  1 = Done incorrectly  2 = Done correctly | Observer | 85 |
|  |  | | The nurse responds to the greetings of the patient at the beginning of the communication | | 0 = Not done  1 = Done incorrectly  2 = Done correctly | Observer | 85 |
|  |  | | The nurse greets the patient at the beginning of the communication | | 0 = Not done  1 = Done incorrectly  2 = Done correctly | Observer | 85 |
|  |  | | The nurse uses a polite and friendly tone during communication | | 0 = Not done  1 = Done incorrectly  2 = Done correctly | Observer | 85 |
|  |  | | Maintaining eye contact | | **5-point Likert scale**  Extremely bad  Extremely good | Patient | 86 |
|  |  | | My doctor acted bossy and domineering at times during my visit today | | Never  Occasionally  Sometimes  Often  Always | Patient | 92 |
|  |  | | Resident identified self and role clearly | | **5-point Likert scale**  Strongly disagree  Strongly agree | Patient and Clinician | 93 |
|  |  | | You were addressed in an appropriate manner by resident | | **5-point Likert scale**  Strongly disagree  Strongly agree | Patient and Clinician | 93 |
|  |  | | Did you feel you were treated with less courtesy than other people? | | No  Yes | Patient | 101 |
|  |  | | Did you feel your doctor acted as if he/she thinks you are not smart? | | No  Yes | Patient | 101 |
|  |  | | Did you feel your doctor acted as if he or she is better than you? | | No  Yes | Patient | 101 |
|  |  | | She/he behaved in a gentle manner | | **10-point Likert scale**  Completely disagree  Completely agree | Patient | 108 |
|  |  | | She/he spoke in a gentle manner | | **10-point Likert scale**  Completely disagree  Completely agree | Patient | 108 |
|  |  | | Overall, the health workers were nice to me | | **10-point Likert scale**  Completely disagree  Completely agree | Patient | 108 |
|  |  | | How do you rate the kindness of the family doctor? | | **5-point Likert scale**  Not satisfied  Completely satisfied | Patient | 111 |
|  |  | | Recognised me | | No  Yes | Patient | 112 |
|  |  | | Greeted me pleasantly | | No  Yes | Patient | 112 |
|  |  | | Addressed me by name | | No  Yes | Patient | 112 |
|  |  | | Looked at me when speaking | | No  Yes | Patient | 112 |
|  |  | | Spoke to me in my language | | No  Yes | Patient | 112 |
|  |  | | Friendliness, warmth, and personal manner of the doctor that treated you | | Very poor  Best | Patient | 114 |
|  |  | | Your doctor greeted you in a way that made you feel comfortable | | **5-point Likert scale**  1 = Strongly disagree  2 = Disagree  3 = Neither agree nor disagree  4 = Agree  5 = Strongly agree | Patient | 119 |
|  |  | | Did the FWA greet you in a friendly way? | | No  Yes | Patient | 123 |
|  |  | | Did the FWA introduce her/himself? (if 1st meeting) | | No  Yes | Patient | 123 |
|  |  | | How would you rate the friendliness and warmth of your physician? | | **5-point Likert scale**  Poor  Fair  Good  Very good  Excellent | Patient | 127 |
|  |  | | Pharmacist was friendly | | **4-point Likert scale**  1 = Strongly disagree or Very poor  2 = Disagree or Poor  3 = Agree or Good  4 = Strongly agree or Excellent | Patient | 128 |
|  |  | | Staff didn’t respond when I greeted him/her | | **5-point Likert scale**  1 = Strongly disagree  2 = Disagree  3 = Neutral  4 = Agree  5 = Strongly agree | Patient | 130 |
|  |  | | Staff smiled during our interaction | | **5-point Likert scale**  1 = Strongly disagree  2 = Disagree  3 = Neutral  4 = Agree  5 = Strongly agree | Patient | 130 |
|  |  | | Staff did not shout at me | | **5-point Likert scale**  1 = Strongly disagree  2 = Disagree  3 = Neutral  4 = Agree  5 = Strongly agree | Patient | 130 |
|  |  | | Friendliness/courtesy of the care provider | | **5-point Likert scale** | Patient | 17 |
|  |  | | The personal manner (courtesy, respect, sensitivity, friendliness) of the person you saw | | **5-point Likert scale** | Patient | 138 |
|  | **Comforting** | | The doctor put me at ease | | **4-point Likert scale**  Not at all  Very much so | Patient | 91 |
|  |  | | My doctor treated me in a way that made me feel comfortable | | **5-point Likert scale**  Strongly disagree  Strongly agree | Patient | 110 |
|  |  | | Ability of the doctor to put you at ease | | Very poor  Best | Patient | 114 |
| **Giving attention** | |  |  | |  |  |  |
|  | **Showing interest** | | Showing interest in you as a person; not acting bored or ignoring what you have to say | | **5-point Likert scale**  Poor  Excellent | patient | 18 |
|  |  | | Being interested in you as a whole person (asking/knowing relevant details about your life, your situation; not treating you as "just a number") | | **5-point Likert scale**  Poor  Excellent | Patient | 4 |
|  |  | | Paid attention to me | | Poor  Fair  Good  Very good  Excellent | Proxy/Caregiver | 6 |
|  |  | | This doctor was interested in me as a person, and not just my illness | | **5-point Likert scale**  Strongly disagree  Strongly agree | Patient | 35 |
|  |  | | The interest they showed in you personally? | | **5-point Likert scale**  Poor  Excellent | Patient | 38 |
|  |  | | The staff showed just the right level of interest | | **5-point Likert scale**  Totally disagree  Totally agree | Patient | 44 |
|  |  | | The doctor seemed interested in me as a person | | **7-point Likert scale**  1 = Very strongly disagree  2 = Strongly disagree  3 = Disagree  4 = Unsure  5 = Agree  6 = Strongly agree  7 = Very strongly agree | Patient | 10 |
|  |  | | The doctor seemed interested in me as a person and not just my illness | | **5-point Likert scale**  Strongly disagree  Strongly agree | Patient | 48 |
|  |  | | This doctor/nurse/health visitor was interested in me as a person not just my illness/interested in the health of my whole family | | **5-point Likert scale**  Strongly disagree  Strongly agree | Patient | 51 |
|  |  | | How much did your doctor seem interested in what you had to say? | | **5-point Likert scale**  Lowest satisfaction  Highest satisfaction | Patient | 52 |
|  |  | | My doctor acted bored at times during the visit today | | **5-point Likert scale**  Lowest satisfaction  Highest satisfaction | Patient | 52 |
|  |  | | My doctor shows interest in me as a person; does not act bored or ignore what I have to say | | **4-point Likert scale**  Poor  Excellent | Patient | 54 |
|  |  | | Information exchange: This doctor was interested in me as a person and not just my illness | | **4-point Likert scale**  Does not apply  Strongly disagree  Disagree  Agree  Strongly agree | Patient | 62 |
|  |  | | Interpersonal skills: It seemed to me that the doctor wasn’t really interested in my physical well-being | | **4-point Likert scale**  Does not apply  Strongly disagree  Disagree  Agree  Strongly agree | Patient | 62 |
|  |  | | Empathy: It seemed to me that the doctor wasn’t really interested in my emotional well-being | | **4-point Likert scale**  Does not apply  Strongly disagree  Disagree  Agree  Strongly agree | Patient | 62 |
|  |  | | Empathy: The doctor should have shown more interest | | **4-point Likert scale**  Does not apply  Strongly disagree  Disagree  Agree  Strongly agree | Patient | 62 |
|  |  | | Do you believe the dermatologist was interested in you as a person? | | **5-point Likert scale**  1 = Not at all  5 = Yes, fully | Patient | 74 |
|  |  | | Interest shown in the patient | | **5-point Likert scale**  Negative  Positive | Patient | 79 |
|  |  | | The dermatologist: Attention of dermatologis during consultation | | **4-point Likert scale**  Very dissatisfied  Very satisfied  (and option “no opinion or not concerned”) | Patient | 81 |
|  |  | | The doctor seemed interested in me | | **4-point Likert scale**  Not at all  Very much so | Patient | 91 |
|  |  | | Showing interest in you as a person | | **5-point Likert scale**  Poor  Fair  Good  Very good  Excellent | Patient | 96 |
|  |  | | The community pharmacist (CP) seemed to take a genuine interest in me as a person | | Strongly disagree  Strongly agree | Patient | 109 |
|  |  | | Interest shown in you as a person | | Very poor  Best | Patient | 114 |
|  |  | | Attention doctor gives to what you have to say | | Missing | Patient | 133 |
|  |  | | The doctor seemed to have other things on his mind | | **5-point Likert scale** | Caregiver | 139 |
|  | **Listening** | | Practitioner uses empathic listening statements when the patient talks about the topic | | **5-point Likert scale**  0 = Not at all  1 = Minimally  2 = To some extent  3 = A good deal  4 = A great extent | Observer | 31 |
|  |  | | Did this doctor listen carefully to you? | | No  Somewhat  Yes  Yes definitely | Patient | 3 |
|  |  | | Really listening… (Paying close attention to what you were saying; not looking at the notes or computer as you were talking) | | **5-point Likert scale**  Poor  Excellent | Patient | 4 |
|  |  | | Did the RTT listen to you? | | **5-point Likert scale**  Not at all  Very much | Patient | 34 |
|  |  | | Sometimes this doctor does not listen to me | | **5-point Likert scale**  Strongly disagree  Strongly agree | Patient | 21 |
|  |  | | Ability to listen to the patient | | **5-point Likert scale**  1 = Poor  5 = Excellent | Patient | 5 |
|  |  | | Listening to you? | | **5-point Likert scale**  Poor  Excellent | Patient | 23 |
|  |  | | Listening to you | | **5-point Likert scale**  Very poor  Very good | Patient | 41 |
|  |  | | During this hospital stay, how often did nurses: listen carefully to you? | | **4-point Likert scale**  Never  Sometimes  Usually  Always | Patient | 12 |
|  |  | | During this hospital stay, how often did doctors: listen carefully to you? | | **4-point Likert scale**  Never  Sometimes  Usually  Always | Patient | 12 |
|  |  | | My provider(s) listens carefully to me | | **4-point Likert scale**  Strongly disagree  Strongly agree | Patient | 42 |
|  |  | | I was listened to when I had worries | | **5-point Likert scale**  Totally disagree  Totally agree | Patient | 44 |
|  |  | | Did the doctors listen carefully to what you had to say? | | **5-point Likert scale** | Patient | 7 |
|  |  | | On this visit I would rate the doctor's ability to really listen to me as | | **5-point Likert scale**  Poor  Excellent | Patient | 24 |
|  |  | | Listened carefully to what I had to say | | **5-point Likert scale**  Strongly disagree  Strongly agree | Patient and Clinician | 47 |
|  |  | | The doctor listened patiently to me | | **5-point Likert scale**  Strongly disagree  Strongly agree | Patient | 48 |
|  |  | | The doctor/nurse/health visitor listened very carefully to what I had to say | | **5-point Likert scale**  Strongly disagree  Strongly agree | Patient | 51 |
|  |  | | Listening to any questions you have and answering clearly; not avoiding or ignoring your questions | | Can’t say  Poor  Fair  Good  Very good  Excellent | Patient | 53 |
|  |  | | How often did the physician listen to you carefully? | | Never  Sometimes  Usually  Always | Patient | 58 |
|  |  | | He/she was able to listen | | **5-point Likert scale**  1 = Not at all  5 = Very much | Patient | 64 |
|  |  | | He/she paid attention to what I was saying | | **5-point Likert scale**  1 = Not at all  5 = Very much | Patient | 64 |
|  |  | | Listened to what the family had to say | | **11-point Likert scale**  The very worst I could imagine  The very best I could imagine  (Or “Doctor didn’t do” or “I don’t know”) | Clinician | 67 |
|  |  | | Listening to what you have to say | | **11-point Likert scale**  The very worst  The very best | Patient | 15 |
|  |  | | Listening carefully to questions | | **4-point Likert scale**  Not at all  Yes | Observer | 16 |
|  |  | | Lending a listening ear | | **4-point Likert scale**  Not at all  Not really  On the whole, yes  Yes | Observer | 16 |
|  |  | | My doctor listens carefully to what I have to say | | **5-point Likert scale**  Strongly disagree  Strongly agree | Patient | 68 |
|  |  | | The physician listened carefully | | Disagree  Agree | Patient | 73 |
|  |  | | When I receive prescriptions from my pharmacist, HCP listens to me when I have a medication question(s) | | 1 = Never  5 = Always  (Or “Not applicable”) | Patient | 75 |
|  |  | | Listening of your symptoms | | **4-point Likert scale**  Very dissatisfied  Very satisfied  (and option “no opinion or not concerned”) | Patient | 81 |
|  |  | | While talking with the patient, the nurse responds to the patient by shaking his/her head and facial expression to express attention to the patient | | 0 = Not done  1 = Done incorrectly  2 = Done correctly | Observer | 85 |
|  |  | | How well did this doctor listen to what you had to say? | | **11-point Likert scale**  Very worst  Very best | Proxy/Caregiver | 90 |
|  |  | | Resident demonstrated good listening skills | | **5-point Likert scale**  Strongly disagree  Strongly agree | Patient and Clinician | 93 |
|  |  | | Letting you tell your story and listening | | **5-point Likert scale**  Poor  Fair  Good  Very good  Excellent | Patient | 96 |
|  |  | | Dermatologist's ability to listen to patient | | **5-point Likert scale**  Poor  Fair  Good  Very good  Excellent | Patient | 98 |
|  |  | | My HIV provider listens carefully to me | | Never  Sometimes  Usually  Always | Patient | 100 |
|  |  | | Did you feel your doctor was not listening to what you were saying? | | No  Yes | Patient | 101 |
|  |  | | She/he listened to me | | **10-point Likert scale**  Completely disagree  Completely agree | Patient | 108 |
|  |  | | The doctor listened to what I said | | **5-point Likert scale**  I disagree completely  I agree completely | Patient | 113 |
|  |  | | Willingness to listen to what you had to say | | Very poor  Best | Patient | 114 |
|  |  | | Your physician listened carefully when you wanted to say something | | **6-point Likert scale**  1 = Strongly disagree  6 = Strongly agree | Patient and Clinician | 118 |
|  |  | | Listened carefully to you | | **5-point Likert scale**  1 = Strongly disagree  2 = Disagree  3 = Neither agree nor disagree  4 = Agree  5 = Strongly agree | Patient | 119 |
|  |  | | During this visit, how often did the physician let you tell your story and listen carefully? | | Never  Sometimes  Always | Patient | 120 |
|  |  | | Physician listens carefully | | Missing | Patient | 131 |
|  |  | | The doctor listened carefully to what I said | | **5-point Likert scale** | Caregiver | 139 |
|  | **Noticing** | | I thought this doctor took notice of me as a person | | **5-point Likert scale**  Strongly disagree  Strongly agree | Patient | 35 |
|  |  | | The doctor paid enough attention to my privacy | | **5-point Likert scale**  Strongly disagree  Strongly agree | Patient | 48 |
|  |  | | I thought this doctor/nurse/health visitor took notice of me as a person | | **5-point Likert scale**  Strongly disagree  Strongly agree | Patient | 51 |
|  |  | | How much did your doctor notice your feelings, especially any sad or worried feelings you might have had? | | **5-point Likert scale**  Lowest satisfaction  Highest satisfaction | Patient | 52 |
|  |  | | The AP was attentive to me | | **5-point Likert scale**  Strongly disagree  Strongly agree | Patient | 59 |
|  |  | | Giving you his/her full attention | | **11-point Likert scale**  The very worst  The very best | Patient | 15 |
|  |  | | Paying attention to the patient | | **4-point Likert scale**  Not at all  Yes | Observer | 16 |
|  |  | | The nurse pays attention to nonverbal communication of the patient (eye contact, tone and intonation, mode of dress, body movements) | | 0 = Not done  1 = Done incorrectly  2 = Done correctly | Observer | 85 |
|  | **Ignoring** | | The RTTs were available towards you? | | **5-point Likert scale**  Not at all  Very much | Patient | 34 |
|  |  | | Did the doctors ignore what you told them? | | **5-point Likert scale** | Patient | 7 |
|  |  | | The doctor ignored some of the things I said | | **5-point Likert scale**  Strongly disagree  Strongly agree | Patient | 48 |
|  |  | | Interpersonal skills: The doctor seemed to brush off my questions | | **4-point Likert scale**  Does not apply  Strongly disagree  Disagree  Agree  Strongly agree | Patient | 62 |
|  |  | | Psychiatrists sometimes ignore what I tell them | | **5-point Likert scale**  Strongly disagree  Strongly agree | Patient | 9 |
|  |  | | My doctor sometimes ignores what I tell him/her | | **5-point Likert scale**  Strongly disagree  Strongly agree | Patient | 68 |
| **Self-efficacy building** | |  |  | |  |  |  |
|  | **Praising** | | The nurse encourages the patient when s/he sees positive points (Personal hygiene, taking medicine, compliance with the regulations of the ward) in the patient | | 0 = Not done  1 = Done incorrectly  2 = Done correctly | Observer | 85 |
|  | **Reassuring** | | Extent of reassurance provided to patient | | **5-point Likert scale**  1 = Poor  5 = Excellent | Patient | 5 |
|  |  | | The extent to which I felt reassured by this doctor was | | **5-point Likert scale**  Poor  Excellent | Patient | 24 |
|  |  | | Reassuring attitude of the MD | | **5-point Likert scale**  Negative  Positive | Patient | 79 |
|  | **Supporting** | | Being positive… (having a positive approach and a positive attitude; being honest but not negative about your problems) | | **5-point Likert scale**  Poor  Excellent | Patient | 4 |
|  |  | | Help you to feel competent as a parent | | **7-point Likert scale**  Not at all perceived  Perceived to a very great extent | Proxy/Caregiver | 25 |
|  |  | | How much did your doctor act supportive and give you the feeling that she/he is a partner with you? | | **5-point Likert scale**  Lowest satisfaction  Highest satisfaction | Patient | 52 |
|  |  | | How much did your provider act supportive and give you the feeling that she/he is partnered with you? | | **5-point Likert scale**  1 = Very little agreement  5 = Very much in agreement | Patient | 56 |
|  |  | | My doctor helps me | | **5-point Likert scale**  Not at all appropriate  Totally appropriate | Patient | 26 |
|  |  | | My doctor is dedicated to helping me | | **5-point Likert scale**  Not at all appropriate  Totally appropriate | Patient | 26 |
|  |  | | He/she gave me encouragement and transmitted optimism | | **5-point Likert scale**  1 = Not at all  5 = Very much | Patient | 64 |
|  |  | | For clinician, occurrence of: Supportive talk | | Coding scheme of utterances | Observer | 71 |
|  |  | | HCP expresses a desire to help me manage my medications | | 1 = Never  5 = Always  (Or “Not applicable”) | Patient | 75 |
|  |  | | Support | | **7-point Likert scale**  Strongly disagree  Strongly agree | Patient | 107 |
| **OTHER** | |  |  | |  |  |  |
|  |  | | Did you feel pressured by doctors in the hospital to have a treatment you were not sure you wanted? | | **5-point Likert scale** | Patient | 7 |
|  |  | | My HCP doesn’t like it when I ask questions | | 1 = All the time  5 = Never | Patient | 49 |
|  |  | | My doctor seemed nervous today | | **5-point Likert scale**  Lowest satisfaction  Highest satisfaction | Patient | 52 |
|  |  | | Inspiring your trust and confidence; never appearing nervous or unsure of himself/herself | | Can’t say  Poor  Fair  Good  Very good  Excellent | Patient | 53 |
|  |  | | I felt my privacy and confidentiality were preserved during my first visit with my doctor | | **5-point Likert scale**  1 = Strong disagreement  3 = Agreement  5 = Strong agreement | Patient | 55 |
|  |  | | I find my doctor easily accessible | | **5-point Likert scale**  Not at all appropriate  Totally appropriate | Patient | 26 |
|  |  | | My psychiatrist is too quiet | | **5-point Likert scale**  Strongly disagree  Strongly agree | Patient | 9 |
|  |  | | The consultation: Use of a computer | | **4-point Likert scale**  Very dissatisfied  Very satisfied  (and option “No opinion or not concerned”) | Patient | 81 |
|  |  | | Whether I was satisfied with the way my doctor and I communicated today | | Never  Occasionally  Sometimes  Often  Always | Patient | 92 |
|  |  | | During my visit today did I feel there were times when my doctor and I miscommunicated | | Never  Occasionally  Sometimes  Often  Always | Patient | 92 |
|  |  | | The doctor treated me fairly | | **5-point Likert scale**  Strongly disagree  Strongly agree | Patient | 101 |
|  |  | | Willing to say do not know | | **7-point Likert scale**  Strongly disagree  Strongly agree | Patient | 107 |
|  |  | | She/he was available for me | | **10-point Likert scale**  Completely disagree  Completely agree | Patient | 108 |
|  |  | | Doctor frustrated with patient communication | | **7-point Likert scale**  1 = Strongly disagree  7 = Strongly agree | Observer | 125 |
|  |  | | Doctor caring | | **7-point Likert scale**  1 = Strongly disagree  7 = Strongly agree | Observer | 125 |
|  |  | | Doctor dominant | | **7-point Likert scale**  1 = Strongly disagree  7 = Strongly agree | Observer | 125 |
|  |  | | Doctor unfriendly | | **7-point Likert scale**  1 = Strongly disagree  7 = Strongly agree | Observer | 125 |
|  |  | | Doctor warm | | **7-point Likert scale**  1 = Strongly disagree  7 = Strongly agree | Observer | 125 |
|  |  | | Doctor enthusiastic | | **7-point Likert scale**  1 = Strongly disagree  7 = Strongly agree | Observer | 125 |
|  |  | | Doctor genuine | | **7-point Likert scale**  1 = Strongly disagree  7 = Strongly agree | Observer | 125 |
|  |  | | Doctor empathic | | **7-point Likert scale**  1 = Strongly disagree  7 = Strongly agree | Observer | 125 |
|  |  | | Patient enthusiastic | | **7-point Likert scale**  1 = Strongly disagree  7 = Strongly agree | Observer | 125 |
|  |  | | Patient dominant | | **7-point Likert scale**  1 = Strongly disagree  7 = Strongly agree | Observer | 125 |

**F7 Dimension 7: Ongoing and iterative process**

|  |  | | **Item** | | **Responses** | **Respondent** | **Instrument ID#** |
| --- | --- | --- | --- | --- | --- | --- | --- |
| **Facilitating patient involvement** | | |  |  |  |  |  |
|  | Co-creating | | The program staff and I discussed my progress together and made changes as necessary | | **5-point Likert scale**  Definitely not true  Completely true | Patient | 32 |
|  | Encouraging | |  | |  |  |  |
|  | Allowing | | Willing to let me ask questions via email/phone | | **7-point Likert scale**  Strongly disagree  Strongly agree | Patient | 107 |
| **Providing information** | |  |  | |  |  |  |
|  | Informing | | Discussed next steps, including any follow-up plans | | Poor  Fair  Good  Very good  Excellent | Proxy/Caregiver | 6 |
|  |  | | Talked about ways to lower the risk of future illness | | **7-point Likert scale**  Very strongly disagree  Very strongly agree | Patient | 19 |
|  |  | | Advised me how to prevent future health problems | | **7-point Likert scale**  Very strongly disagree  Very strongly agree | Patient | 19 |
|  |  | | Discussed next steps including any follow-up plans | | **5-point Likert scale**  Strongly disagree  Strongly agree | Patient and Clinician | 47 |
|  |  | | The clinician indicates the need to review the decision (or deferment) | | 0 = The behaviour is not observed  4 = The behaviour is exhibited to a very high standard | Observer | 8 |
|  |  | | Discussing the future | | **4-point Likert scale**  Not at all  Not really  On the whole, yes  Yes | Observer | 16 |
|  |  | | The health care provider didn’t tell me about follow-up care for when I get home | | **4-point Likert scale**  1 = Strongly disagree  4 = Strongly agree | Patient | 76 |
|  |  | | The doctor (s) explained well to you the future plan of treatment and follow up | | **5-point Likert scale**  Strongly disagree  Strongly agree | Patient | 89 |
|  |  | | Your physician informed you at the end of treatment about the further treatment of your illness | | **6-point Likert scale**  1 = Strongly disagree  6 = Strongly agree | Patient and Clinician | 118 |
|  |  | | Discussed next steps including any follow-up plans | | **5-point Likert scale**  1 = Strongly disagree  2 = Disagree  3 = Neither agree nor disagree  4 = Agree  5 = Strongly agree | Patient | 119 |
|  |  | | Were you told that you could change method or switch to another method if you have any issue with the method you just received? | | No  Yes | Patient | 122 |
|  |  | | Instructions the care provider gave you about follow-up care (if any) | | **5-point Likert scale** | Patient | 17 |
|  |  | | Doctor's instructions about symptoms to report when to seek further care | | Missing | Patient | 133 |
| **Gathering/having needed information** | |  |  | |  |  |  |
|  | Following up | | Did someone from this doctor’s office follow-up to give you results? | | No  Somewhat  Yes  Yes definitely | Patient | 3 |
|  |  | | Information exchange: The doctor told me to call back if I had any questions or problems | | **4-point Likert scale**  Does not apply  Strongly disagree  Disagree  Agree  Strongly agree | Patient | 62 |
|  |  | | Follow-up treatment | | **7-point Likert scale**  Strongly disagree  Strongly agree | Patient | 107 |
| **Self-efficacy building** | |  |  | |  |  |  |
|  | Supporting | | Offering you services for preventing diseases (screening, health checks, immunizations, ) | | **5-point Likert scale**  Poor  Excellent | Patient | 23 |
|  |  | | Helped to plan ahead so I could take care of my illness even in hard times | | **5-point Likert scale**  Almost never  Generally not  Sometimes  Most of the time  Almost always | Patient | 14 |
|  |  | | I am asked if I need help getting home | | No  Yes | Patient | 84 |
|  |  | | Willing to help me find more help | | **7-point Likert scale**  Strongly disagree  Strongly agree | Patient | 107 |
| **OTHER** | |  |  | |  |  |  |
|  |  | | Willing to refer me to a specialist | | **7-point Likert scale**  Strongly disagree  Strongly agree | Patient | 107 |

**F8 Dimension 8: Consequences**

(no items identified)

**F.9 Extra identified dimension: Time and pace**

|  | **Item** | **Responses** | **Respondent** | **Instrument ID#** |
| --- | --- | --- | --- | --- |
|  | Did doctor spend enough time with you? | No  Somewhat  Yes  Yes, definitely | Patient | 3 |
|  | Spent the right amount of time with me | Poor  Fair  Good  Very good  Excellent | Proxy/Caregiver | 6 |
|  | Did the RTT dedicate sufficient time to you? | **5-point Likert scale**  Not at all  Very much | Patient | 34 |
|  | The doctor/nurse seemed rushed when talking to me | **5-point Likert scale**  Strongly disagree  Strongly agree | Patient | 37 |
|  | The doctor/nurse gave directions too fast | **5-point Likert scale**  Strongly disagree  Strongly agree | Patient | 37 |
|  | The doctor/nurse was too abrupt with questions | **5-point Likert scale**  Strongly disagree  Strongly agree | Patient | 37 |
|  | The doctor/nurse did not spend enough time with me | **5-point Likert scale**  Strongly disagree  Strongly agree | Patient | 37 |
|  | Making you feel you had time during consultation? | **5-point Likert**  Poor  Excellent | Patient | 23 |
|  | Giving you enough time | **5-point Likert scale**  Very poor  Very good | Patient | 41 |
|  | My provider(s) spends enough time with me | **4-point Likert scale**  Strongly disagree  Strongly agree | Patient | 42 |
|  | Did you have trouble understanding your doctors because they spoke too fast? | **5-point Likert scale** | Patient | 7 |
|  | Did the doctors give you enough time to say what you thought was important? | **5-point Likert scale** | Patient | 7 |
|  | The amount of time given to me for this visit was | **5-point Likert scale**  Poor  Excellent | Patient | 24 |
|  | Spent the right amount of time with me | **5-point Likert scale**  Strongly disagree  Strongly agree | Patient and Clinician | 47 |
|  | I find it hard to talk with my HCP because he/she is always in such a hurry | 1 = All the time  5 = Never | Patient | 49 |
|  | My HCP spends little time explaining treatment options to me | 1 = All the time  5 = Never | Patient | 49 |
|  | Provide enough time to talk so you don't feel rushed | **7-point Likert scale**  Not at all perceived  Perceived to a very great extent | Proxy/Caregiver | 25 |
|  | Giving you enough time | Doesn’t apply  Very poor  Poor  Neither good nor poor  Good  Very good | Patient | 50 |
|  | The time I was able to spend with this doctor/nurse/health visitor was not long enough to deal with everything I wanted | **5-point Likert scale**  Strongly disagree  Strongly agree | Patient | 51 |
|  | My doctor seemed to be in a hurry | **5-point Likert scale**  Lowest satisfaction  Highest satisfaction | Patient | 52 |
|  | How often did the physician take the time for you? | Never  Sometimes  Usually  Always | Patient | 58 |
|  | The AP was not in a rush | **5-point Likert scale**  Strongly disagree  Strongly agree | Patient | 59 |
|  | My doctor has enough time for me | **5-point Likert scale**  Not at all appropriate  Totally appropriate | Patient | 26 |
|  | Too much time was spent on small talk | Disagree completely  Disagree  So so  Agree  Agree completely | Patient | 61 |
|  | Empathy: The doctor seemed rushed today | **4-point Likert scale**  Does not apply  Strongly disagree  Disagree  Agree  Strongly agree | Patient | 62 |
|  | Quality of time: I feel the doctor did not spend enough time with me | **4-point Likert scale**  Does not apply  Strongly disagree  Disagree  Agree  Strongly agree | Patient | 62 |
|  | He/she gave me time to ask and to talk about the disease | **5-point Likert scale**  1 = Not at all  5 = Very much | Patient | 64 |
|  | Those who provide my psychiatric care sometimes hurry too much when they treat me | **5-point Likert scale**  Strongly disagree  Strongly agree | Patient | 9 |
|  | Psychiatrists usually spend plenty of time with me | **5-point Likert scale**  Strongly disagree  Strongly agree | Patient | 9 |
|  | The physician spent sufficient time on my consultation | **5-point Likert scale**  I do not agree  I fully agree | Patient | 66 |
|  | Taking time | **4-point Likert scale**  Not at all  Not really  On the whole, yes  Yes | Observer | 16 |
|  | Those who provide my medical care sometimes hurry too much when they treat me | **5-point Likert scale**  Strongly disagree  Strongly agree | Patient | 68 |
|  | The physician took sufficient time | Disagree  Agree | Patient | 73 |
|  | Health care provider seemed to want get rid of me as soon as possible | **4-point Likert scale**  1 = Strongly disagree  4 = Strongly agree | Patient | 76 |
|  | My time was NOT well spent in the visit | **5-point Likert scale**  Strongly disagree  Strongly agree | Clinician | 29 |
|  | The dentist takes sufficient time for explanation | **5-point Likert scale**  Strongly disagree  Strongly agree | Patient and Clinician | 78 |
|  | Time spent with the patient | **5-point Likert scale**  Negative  Positive | Patient | 79 |
|  | I am given the news as quickly as possible, even if that means getting the news from a doctor I don’t know well | No  Yes | Patient | 84 |
|  | The nurse does not stop the communication suddenly while the patient is talking | 0 = Not done  1 = Done incorrectly  2 = Done correctly | Observer | 85 |
|  | Giving you enough time | **5-point Likert scale**  Extremely bad  Extremely good | Patient | 86 |
|  | Medical staff's patience when explaining the expected effects of drug therapy before I took the medication | **7-point Likert scale**  Very dissatisfied  Very satisfied | Patient | 87 |
|  | Medical staff's patience when explaining the medication's possible side effects before I took the medication | **7-point Likert scale**  Very dissatisfied  Very satisfied | Patient | 87 |
|  | My doctor seemed to be in a hurry | Never  Occasionally  Sometimes  Often  Always | Patient | 92 |
|  | Did the doctor spend enough time with you? | **9-point Likert scale**  Spent very little time  Spent as much time as required | Patient | 97 |
|  | Overall, the health workers were patient | **10-point Likert scale**  Completely disagree  Completely agree | Patient | 108 |
|  | She/he was efficient | **10-point Likert scale**  Completely disagree  Completely agree | Patient | 108 |
|  | Are you satisfied with the time that the family doctor spends on the consultation? | **5-point Likert scale**  Not satisfied  Completely satisfied | Patient | 111 |
|  | The doctor seemed hurried | **5-point Likert scale**  I disagree completely  I agree completely | Patient | 113 |
|  | The doctor let me set the pace (how fast or slow he went)during the consultation | **5-point Likert scale**  I disagree completely  I agree completely | Patient | 113 |
|  | Spent the right amount of time with you | **5-point Likert scale**  1 = Strongly disagree  2 = Disagree  3 = Neither agree nor disagree  4 = Agree  5 = Strongly agree | Patient | 119 |
|  | I had enough time with the physician | **5-point Likert scale**  Strongly disagree  Disagree  Uncertain  Agree  Strongly agree | Patient | 121 |
|  | Pharmacist spent enough time with you | **4-point Likert scale**  1 = Strongly disagree or Very poor  2 = Disagree or Poor  3 = Agree or Good  4 = Strongly agree or Excellent | Patient | 128 |
|  | Amount of time the care provider spent with you | **5-point Likert scale** | Patient | 17 |
|  | Physician devotes enough time to the visit | Missing | Patient | 131 |
|  | Attitude and time with the physician | **5-point Likert scale**  Very dissatisfied/unwilling  Very satisfied/willing | Patient | 134 |

**F10 ‘Other’ dimension**

|  |  | | **Item** | | | | **Responses** | **Respondent** | **Instrument ID#** |
| --- | --- | --- | --- | --- | --- | --- | --- | --- | --- |
| **Facilitating patient involvement** | | | | |  |  |  |  |  |
|  | **Allowing** | | Did the RTT give you the chance to say everything that was on your mind? | | | | **5-point Likert scale**  Not at all  Very much | Patient | 34 |
|  |  | | The doctor/nurse gave me a chance to say what was on my mind | | | | **5-point Likert scale**  Strongly disagree  Strongly agree | Patient | 37 |
|  |  | | The doctor gave me a chance to say what was really on my mind | | | | **7-point Likert scale**  1 = Very strongly disagree  2 = Strongly disagree  3 = Disagree  4 = Unsure  5 = Agree  6 = Strongly agree  7 = Very strongly agree | Patient | 10 |
|  |  | | The doctor gave me a chance to say or ask all I wanted to | | | | **5-point Likert scale**  Strongly disagree  Strongly agree | Patient | 48 |
|  |  | | Interpersonal skills: I didn’t have a chance to say everything I wanted or to ask all my questions | | | | **4-point Likert scale**  Does not apply  Strongly disagree  Disagree  Agree  Strongly agree | Patient | 62 |
|  |  | | The physician gave me a chance to say or ask all I wanted to | | | | **5-point Likert scale**  Strongly disagree  Disagree  Uncertain  Agree  Strongly agree | Patient | 121 |
|  |  | | The doctor did not really give me a chance to say what was on my mind | | | | **5-point Likert scale** | Caregiver | 139 |
| **Providing information** | | |  |  | | |  |  |  |
|  | **Informing** | | I am given the information by my own doctor, and not by a doctor at the clinic where the ultrasound is done, even if that means a delay of a few days to receive the news | | | | No  Yes | Patient | 84 |
| **Self-efficacy building** | |  |  | | | |  |  |  |
|  | **Supporting** | | Helping you understand the importance of following his or her advice? | | | | **5-point Likert**  Poor  Excellent | Patient | 23 |
